# CONSORT 2025 explanation and elaboration: updated guideline for reporting randomised trials

**DOI:** 10.1136/bmj-2024-081124

**Published:** 2025-04-14

**Authors:** Sally Hopewell, An-Wen Chan, Gary S Collins, Asbjørn Hróbjartsson, David Moher, Kenneth F Schulz, Ruth Tunn, Rakesh Aggarwal, Michael Berkwits, Jesse A Berlin, Nita Bhandari, Nancy J Butcher, Marion K Campbell, Runcie C W Chidebe, Diana Elbourne, Andrew Farmer, Dean A Fergusson, Robert M Golub, Steven N Goodman, Tammy C Hoffmann, John P A Ioannidis, Brennan C Kahan, Rachel L Knowles, Sarah E Lamb, Steff Lewis, Elizabeth Loder, Martin Offringa, Philippe Ravaud, Dawn P Richards, Frank W Rockhold, David L Schriger, Nandi L Siegfried, Sophie Staniszewska, Rod S Taylor, Lehana Thabane, David Torgerson, Sunita Vohra, Ian R White, Isabelle Boutron

**Affiliations:** 1 Oxford Clinical Trials Research Unit, Centre for Statistics in Medicine, University of Oxford, Oxford OX3 7LD, UK; 2 Department of Medicine, Women’s College Research Institute, University of Toronto, Toronto, ON, Canada; 3 UK EQUATOR Centre, Centre for Statistics in Medicine, University of Oxford, Oxford, UK; 4 Centre for Evidence-Based Medicine Odense and Cochrane Denmark, Department of Clinical Research, University of Southern Denmark, Odense, Denmark; 5 Open Patient data Explorative Network, Odense University Hospital, Odense, Denmark; 6 Centre for Journalology, Clinical Epidemiology Programme, Ottawa Hospital Research Institute, Ottawa, ON, Canada; 7 Department of Obstetrics and Gynecology, School of Medicine, University of North Carolina at Chapel Hill, Chapel Hill, NC, USA; 8 Jawaharlal Institute of Postgraduate Medical Education and Research, Puducherry, India; 9 Office of Science Dissemination, Centers for Disease Control and Prevention, Atlanta, GA, USA; 10 Department of Biostatistics and Epidemiology, School of Public Health, Center for Pharmacoepidemiology and Treatment Science, Rutgers University, New Brunswick, NJ, USA; 11 *JAMA Network Open*, Chicago, IL, USA; 12 Centre for Health Research and Development, Society for Applied Studies, New Delhi, India; 13 Child Health Evaluation Services, The Hospital for Sick Children Research Institute, Toronto, ON, Canada; 14 Department of Psychiatry, University of Toronto, Toronto, ON, Canada; 15 Aberdeen Centre for Evaluation, University of Aberdeen, Aberdeen, UK; 16 Project PINK BLUE - Health & Psychological Trust Centre, Utako, Abuja, Nigeria; 17 Department of Sociology and Gerontology, Miami University, OH, USA; 18 Department of Medical Statistics, London School of Hygiene and Tropical Medicine, London, UK; 19 Nuffield Department of Primary Care Health Sciences, University of Oxford, Oxford, UK; 20 Ottawa Hospital Research Institute, Ottawa, ON, Canada; 21 Department of Medicine, Northwestern University Feinberg School of Medicine, Chicago, IL, USA; 22 Department of Epidemiology and Population Health, Stanford University, Palo Alto, CA, USA; 23 Institute for Evidence-Based Healthcare, Faculty of Health Sciences and Medicine, Bond University, University Drive, Robina, QLD, Australia; 24 Departments of Medicine, of Epidemiology and Population Health, of Biomedical Data Science, and of Statistics, and Meta-Research Innovation Center at Stanford (METRICS), Stanford University, Stanford, CA, USA; 25 MRC Clinical Trials Unit at University College London, London, UK; 26 University College London, UCL Great Ormond Street Institute of Child Health, London, UK; 27 NIHR Exeter Biomedical Research Centre, Faculty of Health and Life Sciences, University of Exeter, Exeter, UK; 28 Edinburgh Clinical Trials Unit, Usher Institute-University of Edinburgh, Edinburgh BioQuarter, Edinburgh, UK; 29 *The BMJ*, BMA House, London, UK; 30 Harvard Medical School, Boston, MA, USA; 31 Université Paris Cité, Inserm, INRAE, Centre de Recherche Epidémiologie et Statistiques, Université Paris Cité, Paris, France; 32 Clinical Trials Ontario, MaRS Centre, Toronto, ON, Canada; 33 Duke Clinical Research Institute, Duke University Medical Center, Durham, NC, USA; 34 Department of Emergency Medicine, University of California, Los Angeles, CA, USA; 35 South African Medical Research Council, Cape Town, South Africa; 36 Warwick Research in Nursing, Warwick Medical School, University of Warwick, Coventry, UK; 37 MRC/CSO Social and Public Health Sciences Unit & Robertson Centre for Biostatistics, Institute of Health and Wellbeing, University of Glasgow, Glasgow, UK; 38 Department of Health Research Methods Evidence and Impact, McMaster University, Hamilton, ON, Canada; 39 St Joseph’s Healthcare Hamilton, Hamilton, ON, Canada; 40 York Trials Unit, Department of Health Sciences, University of York, York, UK; 41 Faculty of Medicine and Dentistry, University of Alberta, Edmonton, AB, Canada; 42 Université Paris Cité and Université Sorbonne Paris Nord, Inserm, INRAE, Centre for Research in Epidemiology and Statistics (CRESS), Paris, France; 43 Centre d’Epidémiologie Clinique, Hôpital Hôtel Dieu, AP-HP, Paris, France

## Abstract

Critical appraisal of the quality of randomised trials is possible only if their design, conduct, analysis, and results are completely and accurately reported. Without transparent reporting of the methods and results, readers will not be able to fully evaluate the reliability and validity of trial findings. The CONSORT (Consolidated Standards of Reporting Trials) statement aims to improve the quality of reporting and provides a minimum set of items to be included in a report of a randomised trial. CONSORT was first published in 1996 and was updated in 2001 and 2010. CONSORT comprises a checklist of essential items that should be included in reports of randomised trials and a diagram for documenting the flow of participants through a trial. The CONSORT statement has been updated (CONSORT 2025) to reflect recent methodological advancements and feedback from end users, ensuring that it remains fit for purpose. Here, we present the updated CONSORT explanation and elaboration document, which has been extensively revised and describes the rationale and scientific background for each CONSORT 2025 checklist item and provides published examples of good reporting. The objective is to enhance the use, understanding, and dissemination of CONSORT 2025 and provide guidance to authors about how to improve the reporting of their trials and ensure trial reports are complete, and transparent.

“Readers should not have to infer what was probably done; they should be told explicitly.” Douglas G Altman^1^

Well designed and properly executed randomised trials provide the most reliable evidence on the benefits of healthcare interventions. Biased results from poorly designed and poorly reported trials are wasteful^2^ and can mislead decision making in healthcare at all levels, from treatment decisions for the individual patient to formulation of national public health policies.

Critical appraisal of the quality of randomised trials is possible only if their design, conduct, analysis, and results are completely and accurately reported. However, there is overwhelming evidence that the quality of reporting of randomised trials is not optimal.^3^ Without transparent reporting of methods and results, readers cannot evaluate the reliability and validity of trial findings or extract information for systematic reviews. Trials with inadequate methods are also associated with bias, especially exaggerated treatment effects.^4^ Having a transparent trial protocol is also important because it prespecifies the design and methods used in the trial, such as the primary outcome, thereby reducing the likelihood of undeclared post hoc changes to the trial.^5^

**Summary points**

The CONSORT (Consolidated Standards of Reporting Trials) 2025 statement consists of a 30-item checklist of essential items for reporting the results of randomised trials
This updated explanation and elaboration article describes the rationale and scientific background for each checklist item and provide published examples of good reporting
This explanation and elaboration article provides detailed guidance to enhance the use, understanding, and dissemination of CONSORT 2025, helping to ensure that trial reports are complete and transparent

## Improving the reporting of randomised trials: the CONSORT statement

Issues around poor reporting of research are arguably one of the aspects of research waste that is easiest to fix, as highlighted by the late Doug Altman in 1996.^1^ Efforts to improve the reporting of randomised trials gathered impetus in the mid-1990s and resulted in the Standardized Reporting of Trials (SORT) statement 6 and Asilomar initiative^7^ in 1994. Those initiatives then led to publication of the CONSORT (Consolidated Standards of Reporting Trials) statement in 1996,^8^ which was revised in 2001^9^ and published alongside an accompanying explanation and elaboration document.^10^ CONSORT and the explanation and elaboration document were then updated again in 2010.^11^ ^12^ Similar problems related to the lack of transparent reporting of trial protocols led to the subsequent development of the SPIRIT (Standard Protocol Items: Recommendations for Interventional Trials) statement, published in 2013,^13^ and its accompanying explanation and elaboration document.^14^ In January 2020, the SPIRIT and CONSORT executive groups met in Oxford, UK. As the SPIRIT and CONSORT reporting guidelines are conceptually linked, with overlapping of content and similar dissemination and implementation strategies, the two groups decided that it was more effective to work together.

The CONSORT statement (or simply CONSORT) comprises a checklist of a minimal set of essential items that should be included in reports of randomised trials and a diagram for documenting the flow of participants through a trial. The objective of CONSORT is to provide guidance and recommendations to authors about how to improve the reporting of their trials and ensure their trial reports are complete, and transparent.^11^ ^12^ Readers, clinicians, guideline writers, peer reviewers, and editors can also use CONSORT to help them critically appraise and interpret reports of randomised trials. However, CONSORT is not meant to be used as a quality assessment instrument; it does not specify correct methods to be applied universally. Rather, it reminds the reader of key elements of trial design and conduct related to internal and external validity; when appraising a trial, the reader should critically evaluate the decisions made by the investigators for each of these elements.

Since its publication in 1996, CONSORT has been endorsed by numerous journals worldwide and received global endorsement by prominent editorial organisations, including the World Association of Medical Editors (WAME), International Committee of Medical Journal Editors (ICMJE), and Council of Science Editors (CSE). The endorsement of CONSORT by journals has also been shown to be associated with improved quality of reports of randomised trials.^3^

## Updating the CONSORT statement

CONSORT and SPIRIT are living guidelines, and it is vital that the statements are periodically updated to reflect new evidence, methodological advances, and feedback from users; otherwise, their value and usefulness will diminish over time, rendering them no longer fit for purpose. Updating the SPIRIT 2013 and CONSORT 2010 statements and accompanying explanation and elaboration documents together was also an opportunity to align both checklists and to provide users with consistent guidance in the reporting of trial design, conduct, analysis, and results from trial protocol to final publication. Harmonising the reporting recommendations will improve usability and adherence, leading to more complete and accurate reporting.^15^

The methods used to update the CONSORT statement followed the EQUATOR Network guidance for developers of health research reporting guidelines^16^ and have been described in detail elsewhere.^17^ In brief, we first conducted a scoping review of the literature related to randomised trials to identify suggestions for changes or additions to CONSORT 2010.^18^ We also developed a project specific database (SCEBdb) for empirical and theoretical evidence related to CONSORT and risk of bias in randomised trials.^19^ This evidence was combined with evidence from existing CONSORT extensions (Harms,^20^ Outcomes,^21^ Non-pharmacological Treatment^22^), other related reporting guidelines (Template for Intervention Description and Replication (TIDieR)^23^), and evidence and recommendations from other sources. From these sources, we generated a list of potential modifications or additions to CONSORT, which we presented to end users for feedback in a large international online Delphi survey involving over 300 participants. The Delphi survey results were discussed at a two-day online expert consensus meeting attended by 30 invited international experts. We then drafted the updated CONSORT checklist and revised it based on further feedback from meeting attendees.

The CONSORT 2025 statement is published elsewhere,^24^ where full details of the changes to the checklists and rationale for the changes are described. To help facilitate implementation of CONSORT 2025, we have also developed an expanded version of the CONSORT 2025 checklist, with bullet points. The expanded checklist comprises an abridged version of elements presented in this CONSORT 2025 explanation and elaboration document, with examples and references removed (appendix 1).

## Purpose and main changes to the CONSORT 2025 explanation and elaboration

We modelled our approach for developing the CONSORT 2025 explanation and elaboration document on the procedures used for the CONSORT 2010 explanation and elaboration document.^12^ We present each CONSORT 2025 checklist item (table 1) with at least one recent published example of good reporting, followed by an explanation of the rationale for including the item and main issues to address, and a list selected references to current empirical and theoretical evidence. Members of the core writing group (SH, A-WC, GSC, AH, DM, KFS, IB) developed a draft of individual checklist items, which was then comprehensively revised based on comments from all authors.

**Table 1:** CONSORT 2025 checklist of information to include when reporting a randomised trial

Section/topic	No	CONSORT 2025 checklist item description
**Title and abstract**		
Title and structured abstract	1a	Identification as a randomised trial
	1b	Structured summary of the trial design, methods, results, and conclusions
**Open science**		
Trial registration	2	Name of trial registry, identifying number (with URL) and date of registration
Protocol and statistical analysis plan	3	Where the trial protocol and statistical analysis plan can be accessed
Data sharing	4	Where and how the individual de-identified participant data (including data dictionary), statistical code and any other materials can be accessed
Funding and conflicts of interest	5a	Sources of funding and other support (eg, supply of drugs), and role of funders in the design, conduct, analysis, and reporting of the trial
	5b	Financial and other conflicts of interest of the manuscript authors
**Introduction**		
Background and rationale	6	Scientific background and rationale
Objectives	7	Specific objectives related to benefits and harms
**Methods**		
Patient and public involvement	8	Details of patient or public involvement in the design, conduct and reporting of the trial
Trial design	9	Description of trial design including type of trial (eg, parallel group, crossover), allocation ratio, and framework (eg, superiority, equivalence, non-inferiority, exploratory)
Changes to trial protocol	10	Important changes to the trial after it commenced including any outcomes or analyses that were not prespecified, with reason
Trial setting	11	Settings (eg, community, hospital) and locations (eg, countries, sites) where the trial was conducted
Eligibility criteria	12a	Eligibility criteria for participants
	12b	If applicable, eligibility criteria for sites and for individuals delivering the interventions (eg, surgeons, physiotherapists)
Intervention and comparator	13	Intervention and comparator with sufficient details to allow replication. If relevant, where additional materials describing the intervention and comparator (eg, intervention manual) can be accessed
Outcomes	14	Prespecified primary and secondary outcomes, including the specific measurement variable (eg, systolic blood pressure), analysis metric (eg, change from baseline, final value, time to event), method of aggregation (eg, median, proportion), and time point for each outcome
Harms	15	How harms were defined and assessed (eg, systematically, non-systematically)
Sample size	16a	How sample size was determined, including all assumptions supporting the sample size calculation
	16b	Explanation of any interim analyses and stopping guidelines
Randomisation:		
Sequence generation	17a	Who generated the random allocation sequence and the method used
	17b	Type of randomisation and details of any restriction (eg, stratification, blocking, and block size)
Allocation concealment mechanism	18	Mechanism used to implement the random allocation sequence (eg, central computer/telephone; sequentially numbered, opaque, sealed containers), describing any steps to conceal the sequence until interventions were assigned
Implementation	19	Whether the personnel who enrolled and those who assigned participants to the interventions had access to the random allocation sequence
Blinding	20a	Who was blinded after assignment to interventions (eg, participants, care providers, outcome assessors, data analysts)
	20b	If blinded, how blinding was achieved and description of the similarity of interventions
Statistical methods	21a	Statistical methods used to compare groups for primary and secondary outcomes, including harms
	21b	Definition of who is included in each analysis (eg, all randomised participants), and in which group
	21c	How missing data were handled in the analysis
	21d	Methods for any additional analyses (eg, subgroup and sensitivity analyses), distinguishing prespecified from post hoc
**Results**		
Participant flow, including flow diagram	22a	For each group, the numbers of participants who were randomly assigned, received intended intervention, and were analysed for the primary outcome
	22b	For each group, losses and exclusions after randomisation, together with reasons
Recruitment	23a	Dates defining the periods of recruitment and follow-up for outcomes of benefits and harms
	23b	If relevant, why the trial ended or was stopped
Intervention and comparator delivery	24a	Intervention and comparator as they were actually administered (eg, where appropriate, who delivered the intervention/comparator, whether participants adhered, whether they were delivered as intended (fidelity))
	24b	Concomitant care received during the trial for each group
Baseline data	25	A table showing baseline demographic and clinical characteristics for each group
Numbers analysed, outcomes, and estimation	26	For each primary and secondary outcome, by group: • the number of participants included in the analysis • the number of participants with available data at the outcome time point • result for each group, and the estimated effect size and its precision (such as 95% confidence interval) • for binary outcomes, presentation of both absolute and relative effect size
Harms	27	All harms or unintended events in each group
Ancillary analyses	28	Any other analyses performed, including subgroup and sensitivity analyses, distinguishing prespecified from post hoc
**Discussion**		
Interpretation	29	Interpretation consistent with results, balancing benefits and harms, and considering other relevant evidence
Limitations	30	Trial limitations, addressing sources of potential bias, imprecision, generalisability, and, if relevant, multiplicity of analyses

We have made a number of substantive changes to the CONSORT 2025 explanation and elaboration document. These reflect changes to the CONSORT 2025 checklist: for example, the addition of new items related to data sharing (item 4); how patients and the public were involved in the design, conduct, and reporting of the trial (item 8); how harms were assessed (item 15); how missing data were handled (item 21c); and details of how the intervention and comparator were actually administered (item 24a). We deleted the checklist item on generalisability of trial findings, which is now incorporated under trial limitations (item 30).

Some changes to the CONSORT 2025 checklist also reflect the integration of aspects of the CONSORT extensions related to harms,^20^ outcomes,^21^ and non-pharmacological treatments^22^; and TIDieR.^23^ We have reorganised the structure of the CONSORT checklist, including a new section on open science, which includes items that are conceptually linked such as trial registration (item 2); where the trial protocol and statistical analysis plan can be accessed (item 3); sharing of de-identified participant level data and statistical code (item 4); and funding and conflicts of interest (item 5). We have aligned the item wording between the CONSORT and SPIRIT 2025 checklists. We have retained some of the same language in the explanation and elaboration text from the previous CONSORT 2010 explanation and elaboration,^12^ where it was felt that no changes were required and no new evidence existed to modify our discussion of the issues. The following sections list checklist items (table 1) with examples and explanations.

## CONSORT 2025: Title and abstract 

### Item 1a: Identification as a randomised trial

#### Example

“Efficacy and Safety of Early Administration of 4-Factor Prothrombin Complex Concentrate in Patients With Trauma at Risk of Massive Transfusion: The PROCOAG Randomized Clinical Trial.”^25^

#### Explanation

The ability to identify a report of a randomised trial in a bibliographic database depends to a large extent on how it was indexed. Indexers might not classify a report as a randomised trial if the authors do not explicitly report this information.^26^ To help ensure that a study is appropriately indexed and easily identified, authors should use the word “randomised” in the title to indicate that the participants were randomly assigned to their comparison groups.

### Item 1b: Structured summary of the trial design, methods, results, and conclusions

#### Explanation

Transparent and sufficiently detailed abstracts are important because readers often base their assessment of a trial on such information. Some readers use an abstract as a screening tool to decide whether to read the full article. However, not all trials are freely available and some health professionals and other users do not have access to the full trial reports.^27^

A journal abstract should contain sufficient information about a trial to serve as an accurate record of its conduct and findings, providing optimal information about the trial within the space constraints and format of a journal. A properly constructed and written abstract helps individuals to assess quickly the relevance of the findings and aids the retrieval of relevant reports from electronic databases.^28^ The abstract should accurately reflect what is included in the full journal article and should not include information that does not appear in the body of the paper. In addition, abstracts should not be a distorted representation of the trial results. Studies comparing information reported in a journal abstract with that reported in the text of the full publication have found claims that are inconsistent with, or missing from, the body of the full article.^29^ ^30^ ^31^ ^32^ Abstracts are also frequently reported with spin defined as a distorted representation of the study results.^33^ ^34^ ^35^ Authors should avoid selectively reporting only statistically significant secondary outcomes or subgroup analyses. Conversely, omitting important harms from the abstract could seriously mislead interpretation of the trial findings and benefit-to-harms balance that is critical for decision making.^36^ ^37^

An extension to CONSORT 2001 provided a list of essential items that authors should include in a journal (or conference) abstract when reporting the main results of a randomised trial.^38^ A systematic review of 10 meta-research studies examined the reporting quality of abstracts of randomised trials and found improvements in reporting following publication of this extension.^39^ Table 2 provides a list of essential items to include in an abstract; it is based on the CONSORT for Abstracts extension^40^ and has been updated to reflect changes made to the main CONSORT checklist. We strongly recommend the use of structured abstracts for reporting randomised trials. They provide readers with information about the trial under a series of headings pertaining to the design, conduct, analysis and interpretation.^41^ Some studies have found that structured abstracts offer greater value and information coverage than the more traditional descriptive abstracts^42^ ^43^ and allow readers to find information more easily.^44^ We recognise that journals have their own structure for reporting abstracts. It is not our intention to suggest changes to these formats, but to recommend what information should be reported.

**Table 2:** Items to include when reporting a randomised trial in a journal abstract

Item	Description
Objectives	Specific objectives
Trial design	Description of the trial design (eg, parallel group, cluster) and framework (eg, superiority, equivalence, non-inferiority, exploratory)
Methods	
Participants	Eligibility criteria for participants and settings where the trial was conducted
Interventions	Intervention(s) and comparator(s) intended for each group
Outcome	Primary outcome(s)
Randomisation	How participants were allocated to interventions (eg, centralised computer generated randomisation)
Blinding	Who was blinded after assignment to interventions (eg, participants, care providers, outcome assessors)
Results	
Numbers randomised	Number of participants randomised to each group
Numbers analysed	For the primary outcome, number of participants analysed in each group
Outcome	For the primary outcome, a result for each group and the estimated effect size and its precision
Harms	Important harms or unintended events for each group
Conclusions	General interpretation of the results
Trial registration	Name of trial register and identifying number
Funding	Sources of funding

## CONSORT 2025: Open science

Open science practices are increasingly endorsed worldwide. There are many definitions of open science and many different goals of openness may be surmised under this term. The United Nations Educational, Scientific and Cultural Organisation defines open science as “an inclusive construct that combines various movements and practices aiming to make multilingual scientific knowledge openly available, accessible and reusable for everyone, to increase scientific collaborations and sharing of information for the benefits of science and society, and to open the processes of scientific knowledge creation, evaluation and communication to societal actors beyond the traditional scientific community.”^45^ Open science practices apply to the design, conduct, documentation, and reporting of randomised trial protocols and completed trials.

Some of the checklist items in this section, such as trial registration, were previously included in both SPIRIT and CONSORT. To give them more prominence they are now included under the Open Science heading. However, data sharing is a new item for CONSORT. Furthermore, while no item is dedicated to authorship, we recommend that authors are transparent on whether and how artificial intelligence tools may have been involved in the writing of the manuscript.^46^

### Item 2: Name of trial registry, identifying number (with URL) and date of registration

#### Example

“This study was registered in the Iranian Registry of Clinical Trials under the code IRCT20150531022498N30: https://en.irct.ir/trial/41031. Registered on July 26, 2019.”^47^

#### Explanation

The consequences of non-publication of entire trials (ie, publication bias),^48^ ^49^ ^50^ and of selective reporting of outcomes and analyses within trials, have been well documented.^51^ ^52^ ^53^ For almost 40 years, there have been growing calls to address these practices. Today, a ubiquitous view recommends trial registration as the best practice to achieve this goal, and inform policymakers and potential participants about ongoing trials. Registering clinical trials, before any assignment of participants, with unique trial identification numbers and other basic information about the trial so that essential details are made publicly available, is a minimum best practice.^54^ ^55^ ^56^ ^57^ Serious problems of withholding data^58^ led to renewed efforts to ensure registration of randomised trials. The World Health Organization (WHO) states that “the registration of all interventional trials is a scientific, ethical and moral responsibility.”^59^

In September 2004, the ICMJE established a policy that it would only consider trials for publication if they had been registered before the enrolment of the first participant.^60^ This policy resulted in a substantial increase in the number of trials being registered.^61^ However, some trials are still not registered.^49^ The ICMJE gives guidance on acceptable registries (https://www.icmje.org/about-icmje/faqs/clinical-trials-registration/ ) and also accepts registration in WHO primary registries (https://www.who.int/clinical-trials-registry-platform/network/primary-registries) and ClinicalTrials.gov. Registers charging a fee to view their content should be avoided to ensure equity of access for everyone, including patients and the public.

The Transparency and Openness Promotion (TOP) guidelines, endorsed and used by thousands of journals, recommends trial registration.^62^ In a survey of 168 high impact factor medical journals’ “Instructions to authors” in 2014, 78 journals stated that all recent clinical trials must be registered as a requirement of submission to that journal.^63^ A more recent survey in 2019 of surgical journals publishing randomised trials found that 53 of 82 journals mandated prospective registration.^64^

Despite recommendations, and mandates in some jurisdictions for clinical trialists to register their trial and evidence that registration deters selective reporting, this is still not happening universally.^65^ ^66^ Authors should provide the name of the registry, the trial’s associated registration number, date of registration and, where possible, the URL for the trial’s registration. We recommend that authors also report whether (or when) the trial results are available on the associated trial register.

Despite the considerable increase in clinical trial registration, there is a strong body of evidence showing the lack of access to trial results.^67^ ^68^ ^69^ ^70^ The latest version of the Declaration of Helsinki states that “Researchers have a duty to make publicly available the results of their research on human participants and are accountable for the timeliness, completeness, and accuracy of their reports . . . Negative and inconclusive as well as positive results must be published or otherwise made publicly available.” In 2015, WHO published a new statement on the public disclosure of trial results, which requests that “the key outcomes are to be made publicly available within 12 months of study completion by posting to the results section of the primary clinical trial registry. Where a registry is used without a results database available, the results should be posted on a free-to-access, publicly available, searchable institutional website of the Regulatory Sponsor, Funder or Principal Investigator.” Some legislations are also in place in the US, UK, and Europe requesting the posting of trial results on clinical trials registry within 12 months after study completion.^71^ ^72^ ^73^

Authors should indicate whether the trial results are publicly posted to the trial registry, as a preprint (with URL citation) or as published articles (with citations).

### Item 3: Where the trial protocol and statistical analysis plan can be accessed

#### Example

“The full trial protocol and the Statistical Analysis Plan can be accessed in the Supplementary Material”.^74^ This article and supplementary material are open access.

#### Explanation

A protocol for the complete trial (rather than a protocol of a specific procedure within a trial, such as for the intervention) is important because it prespecifies the methods of the randomised trial, for example, the primary outcome (item 14). Having a protocol provides important context to interpret a trial, implement its findings, and facilitate replication and appraisal of risk of bias. It can also help to restrict the likelihood of undeclared post hoc changes to the trial methods and selective outcome reporting (item 10).^75^ ^76^ ^77^ Elements that are important to address in the protocol for a randomised trial are described in the SPIRIT 2025 statement.^78^

A protocol may either include the full statistical analysis plan or may include a section outlining the main principles while referencing and reporting the full statistical analysis plan as a separate, more detailed document. The statistical analysis plan typically includes details about several aspects of the clinical trial, such as the data analysis plan for the primary outcome(s) and all secondary outcomes. Details about what to include in a statistical analysis plan can be found elsewhere.^79^

The protocol should be signed off by the trial steering committee before the allocation of any participants and data collection. Similarly, the full statistical analysis plan should be signed off by the trial steering committee before the dataset is closed for analysis. This allows transparent documentation of any subsequent changes to either document. In many trials, changes to the protocol and statistical analysis plan may happen after the trial onset for legitimate reasons (eg, in response to challenges that were not anticipated or new evidence). In these cases, each iteration of the protocol and statistical analysis plan should record the changes along with their rationale and timing.

There are several options for authors to consider to ensure their trial protocol, and full statistical analysis plan where applicable, are accessible to interested readers. The protocol and full statistical analysis plan (and their various iterations) can be stored in a repository, such as the Open Science Framework, which is free to use and access for all readers. Openness and accessibility are core elements of open science (see Open science section). Trial protocols and statistical analysis plans can also be published in journals such as *Trials* and *BMJ Open*. Open access publication would ensure that any reader, including patients and the public, can access the document. Trial registration (item 2) will also ensure that a minimum set of trial protocol details are available as part of the trial’s registration (https://www.who.int/clinical-trials-registry-platform), but often a registration record leaves large ambiguity about key protocol issues and statistical analyses. The more detailed trial protocol can also be posted on most clinical trial registries.

Ideally, the authors should give access to the protocol signed off by the trial steering committee before the allocation of any participants and data collection with any subsequent changes with their rationale and timing.

When submitting a completed trial report, trial authors can include their protocol as a supplemental document or provide a URL to its location. Such documentation can facilitate peer review and help identify reporting biases.

### Item 4: Where and how the individual de-identified participant data (including data dictionary), statistical code, and any other materials can be accessed

#### Examples

“All data requests should be submitted to the corresponding author (AR) for consideration as agreed in our publication plan. Access to anonymised data may be granted following review with the Trial Management Group and agreement of the chief investigator (AR).”^80^

“Deidentified data collected and presented in this study, including individual participant data and a data dictionary defining each field in the set, will be made available upon reasonable request after publication of this Article, following approval by regulatory authorities. Data can be requested by contacting the corresponding author.”^81^

#### Explanation

Data and code sharing can take the transparency of trial reporting to a different, more desirable level. Sharing individual de-identified participant data would be helpful in many ways: verifying results and increasing trust; using data more extensively for secondary analyses; and using data for individual patient data meta-analysis (IPD MA). Data sharing is also associated with increased citations^82^ (ie, broader dissemination). Some trial groups have worked collaboratively to conduct IPD MA.^83^ However, for most randomised trials, data sharing does not happen.^84^ ^85^ ^86^ ^87^ ^88^ During the covid-19 pandemic, there were many examples of authors’ intentions to share data that then did not transpire (ie, they did not share their data).^87^ ^89^ There is increasing concern that some trials are fraudulent or considered to be so-called zombie trials, which becomes evident only on inspection of the raw data.^90^ ^91^ However, even if zombie trials are not as prevalent as feared, genuine trials can have such an important role and high value that it is important to maximise their utility by making them more open. Detailed documentation of sharing plans may help in this direction.^92^

All data sharing should abide by the principle of being as open as possible and as closed as necessary throughout a randomised trial’s life cycle (from SPIRIT to CONSORT). It is important to ensure that all the appropriate permissions are included on the patient consent forms. Trials cannot share data that are not fully anonymised without the appropriate patient consent, and full anonymisation can be difficult. Care must be taken to share participant data appropriately to maintain confidentiality. Suitable mechanisms must be in place to appropriately de-identify participant data, and data should only be shared in a safe and secure manner that fits with the consent obtained from participants.

Data sharing typically involves sharing: the underlying data generated from the trial’s conduct; a data dictionary (ie, structure, content, and meaning of each data variable); and other relevant material(s) used as part of the trial’s analysis such as the trial protocol, data management plan, statistical analysis plan, and code used to analyse the data. A trial’s data can be shared in a variety of ways, such as via an institutional repository (eg, belonging to the university associated with the trial’s coordinating centre) and/or a public-facing repository, or by having a bespoke process to provide data. Often, a data use agreement is necessary, which will, at a minimum: prohibit attempts to reidentify or contact trial participants; address any requirements regarding planned outputs of the proposed research (eg, publication and acknowledgment requirements); and prohibit non-approved uses or further distribution of the data.^93^

In a growing number of jurisdictions, funders such as the National Institute for Health (NIH),^94^ in the US and the National Institute for Health and Care Research (NIHR) in the UK, alongside other funders such as the Gate’s Foundation, now require researchers to share their data and make the results publicly available for anyone to read. Similarly, some journals are also requiring authors to include a data sharing statement as part of the article submission process (eg, *Annals of Internal Medicine*, *The BMJ*, *JAMA Network* journals, *PLoS Medicine*).

The process of signalling how data sharing will be achieved is often contained in a data management plan but may also be found in the trial protocol or statistical analysis plan. More complete details regarding developing a data management plan are beyond the scope of this paper. Such details can be found elsewhere.^95^ Authors should provide some description of where these details can be found (eg, name of repository and URL to data, code, and materials). Sharing may also entail embargo periods, and if so, the choice of an embargo should be justified and its length should be stated.^96^ If data (or some parts thereof) cannot be shared, the reasons for this should be reported and should be sensible and following ethical principles.

For more complex trials (eg, types of talking therapies, physiotherapy), additional materials to share might include a handbook and/or video to detail the intervention.^93^ Often these can be shared much more freely than the data, as there are fewer issues with confidentiality.

### Item 5a: Sources of funding and other support (eg, supply of drugs), and role of funders in the design, conduct, analysis, and reporting of the trial

#### Examples

“Grant support was received for the intervention from Plan International and for the research from the Wellcome Trust and Joint United Nations Programme on HIV/AIDS (UNAIDS). The funders had no role in study design, data collection and analysis, decision to publish, or preparation of the manuscript.”^97^

“Funding: Merck Sharp and Dohme . . . The study funder had a role in the study design, data collection, data analysis, data interpretation, and writing of the report.”^98^

The article also states that “Merck employees LY, SB, and PB were involved in the conceptualisation of the study, formal analysis, the investigation process, development of the methodology, project administration, drafting the manuscript, and had critically reviewed and edited the manuscript.”^98^

#### Explanation

Reporting the funding source(s), and the exact roles of the trial funders, provides important context for readers of a trial report when ascertaining overall methodological rigor (eg, relevance of the type of comparator intervention and eligibility criteria for patients) and risk of bias (eg, selective reporting of favourable results). The trial report should therefore describe details of all funders and the types of funding, as well as the role of the funder in trial design (ie, protocol development), conduct, data analysis, and reporting (ie, interpretation, manuscript writing, and dissemination of results). This should include whether the funder controlled the final decision regarding any of these aspects of the trial, and any mechanisms introduced to minimise funder influence. If the funder had no direct involvement in the trial, that should be stated.

A randomised trial requires considerable funding, typically from pharmaceutical or device companies (industry funding); or from research councils or other scientific or private foundations, or governmental or non-governmental organisations (non-industry funding).^99^ One study of trials conducted between 2010 and 2015 estimated the median cost per phase 3 drug company trial at $21.4m (£17.21m; €20.7m),^100^ with substantial variation. The mean cost of clinical trials funded by the NIHR in the UK, reflecting differences in the research and care infrastructure already funded, was lower, but still sizeable—approximately £1.3m—with considerable variation.^101^ The various types of funders differ in their overall agenda, their reasons for funding a trial, and their propensity to influence the trial.

Funding of a trial typically involves direct monetary support, but financial support may also be provided indirectly in the form of free trial drugs, equipment, or services (eg, statistical analysis or use of medical writers).^102^ Among the most highly cited clinical trials published in 2019 to 2022, two thirds were funded by industry sponsors, many of whom also provided industry analysts and coauthors.^103^

Industry funding of trials is associated with conclusions that favour the experimental intervention. A systematic review of 75 methodological studies, comparing industry funded studies with non-industry funded studies (mostly randomised trials), reported that industry funded studies had favourable conclusions more often than non‐industry funded studies (risk ratio 1.34; 95% confidence interval (CI) 1.19 to 1.51).^104^ Industry funded trials may also report more favourable results (ie, larger estimates of intervention effects) than comparable trials that are funded by non-industry sources. One review of eight published meta-epidemiological studies reported that intervention effects (odds ratios) from industry funded trials were, on average, exaggerated by 5% (95% CI −6% to 15%), although the result was imprecise and consistent with chance findings. However, trials with a high risk of industry funder influence (eg, on trial design, conduct, analysis, and reporting) exaggerated effect estimates by 12% (95% CI 3% to 19%).^105^ Undue influence on trials from non-industry funders with a strong interest in a specific trial result has been described,^106^ but has been studied much less.

A review of 200 trials published in 2015^107^ found that 178 (89%) publications included a funding statement. However, in half of the publications, the role of funder was not reported; in the other half, the reporting was often unclear or incomplete; and undisclosed funding from a for-profit organisation was found in 26 of 54 trials reporting only not-for-profit funding. Another study surveyed authors of 200 trials fully funded by industry and found that funders had been involved in the design of 173 trials (87%), in the data analysis of 146 trials (73%), and in the reporting of 173 trials (87%).^102^ No clear consensus exists on a monetary threshold for when funding from a source with conflict of interest becomes problematic. It is also unclear whether commercial funding is less important than the degree and type of funder influence on trial design, conduct, analysis, and reporting.

### Item 5b: Financial and other conflicts of interest of the manuscript authors

#### Example

“SYR reports grants from Amgen, Astellas, Daiichi Sankyo, Eisai, Merck, Roche, Zymeworks, Indivumed, MSD, Ono/Bristol Myers Squibb, AstraZeneca, BI, Taiho, Lilly, SN Bioscience. SRF has received honoraria as an invited speaker for Lilly, Eisai, Daiichi Sankyo, MSD, and Ono/Bristol Myers Squibb; has participated on advisory boards for Amgen and Indivumed; and has served as an advisor for Astellas, Daiichi Sankyo, Eisai, LG Biochem, Merck Sharpe Dohme, Ono/Bristol Myers Squibb, and AstraZeneca. D-YO reports grants from AstraZeneca, Novartis, Array, Eli Lilly, Servier, BeiGene, Merck Sharpe Dohme, and Handok; and has participated on a data safety monitoring board or advisory board for AstraZeneca, Novartis, Genentech/Roche, Merck Serono, Bayer, Taiho, ASLAN, Halozyme, Zymeworks, Bristol Myers Squibb/Celgene, BeiGene, Basilea, Turning Point, Yuhan, Arcus Biosciences, IQVIA, and Merck Sharpe Dohme. M-HR reports research grants from AstraZeneca; consulting fees from Bristol Myers Squibb, Ono, Lilly, Merck Sharpe Dohme, Novartis, Daiichi Sankyo, AstraZeneca, Sanofi, and Astellas; and has received honoraria for lectures, presentations, speakers bureaus, or educational events from Bristol Myers Squibb, Ono, Lilly, Merck Sharpe Dohme, Novartis, Daiichi Sankyo, AstraZeneca, Sanofi, and Astellas . . .

“LY, SB and PB report full-time employment by Merck Sharp and Dohme, a subsidiary of Merck (Rahway, NJ, USA), and stock ownership in Merck. LSW reports consulting fees from Amgen; and has received honoraria for lectures, presentations, speakers bureaus, or educational events from Novartis, Bristol Myers Squibb, Merck Sharpe Dohme, Roche, and Amgen.

“PY, YB, JLee, MGF, JLi, MAL, TC, SQ, SL, and HP declare no competing interests.”^98^

#### Explanation

Disclosure of authors’ conflicts of interest provides important context for readers of a trial report when ascertaining the overall methodological rigor of a trial (eg, relevance of the type of comparator intervention and eligibility criteria for patients) and risk of bias (eg, selective reporting of favourable results). Conflicts of interest of all trial manuscript authors should be reported, along with any procedures to reduce the risk of conflicts of interest influencing the trial’s design, conduct, analysis, or reporting.

Conflicts of interest can be defined as “a set of circumstances that creates a risk that professional judgement or actions regarding a primary interest will be unduly influenced by a secondary interest.”^108^ In the context of authors of a trial report, conflicts of interest imply a risk that investigators’ personal interests and allegiances, or ties with companies or organisations, have undue influence on the design, conduct, analysis, or reporting of a trial. The concept implies a risk of influence and is not indicative of actual wrongdoing.

Conflicts of interest are most often associated with the drug and device industries. Types of financial ties include salary support or grants; ownership of stock or options; honorariums (eg, for advice, authorship, or public speaking); paid consultancy or service on advisory boards and medical education companies; and receipt of patents or patents pending. An analysis of 200 trials from 2015 reported that 57% of trials had at least one author declaring financial conflicts of interests.^109^

Conflicts of interest may also exist with support from or affiliation with government agencies, charities, and professional and civic organisations. Non-financial conflicts of interest include academic commitments; personal or professional relationships; and political, religious, or other affiliations with special interests or advocacy positions. An analysis of 200 trials found that 4% of trials had at least one author declaring non-financial conflicts of interest.^109^ There is ongoing discussion on the association between a problematic non-financial conflict of interest and a reasonable point of view.^110^

A cross sectional study of 190 randomised trials, published in core clinical journals, found that trials with authors’ conflicts of interest had more positive results than trials without. The presence of a financial tie was associated with a positive study outcome (odds ratio 3.23; 95% CI 1.7 to 6.1). This association was also present after adjustment for the study funding source (odds ratio 3.57; 95% CI 1.7 to 7.7).^111^

Although financial conflicts of interest are often declared in trials,^109^ the declarations are generally imprecise, and undisclosed conflicts are common.^112^ A systematic review of studies comparing financial conflicts of interest declared in medical publications or guidelines (not only randomised trials) with declarations in payment databases (eg, the Open Payments Database) found that the median percentage of authors with “non-concordant” disclosures was 81%.^112^ A study including only randomised trials found that 35 (30%) of 115 authors from non-industry funded trials had undisclosed conflicts of interest whereas that was the case for 102 (50%) of 203 authors from industry funded trials.^113^ For financial conflicts that cannot be tracked to public databases and for non-financial conflicts, the rate of non-disclosure is unknown but it is likely to be even higher.

## CONSORT 2025: Introduction

### Item 6: Scientific background and rationale

#### Example

“Most problems with shoulder pain are managed in primary care by physiotherapists and GPs [general practitioners] . . . Evidence from small, short-term trials suggests that physiotherapist-prescribed exercise is promising. However, a Cochrane review highlighted the insufficient evidence about the treatment's long-term clinical effectiveness and cost-effectiveness. Despite widespread provision, uncertainty exists about which types of exercise and levels of physiotherapy supervision are associated with the best outcomes. This evidence is limited by problems in study design and lack of comparator groups. Progressive resistance training to improve muscular strength, whether supervised or home based, has been identified as a core component of exercise for patients with rotator cuff disorders. Subacromial corticosteroid injections are commonly used to reduce local tissue inflammation and pain. Compared with placebo, corticosteroid injections have short-term benefit in the shoulder, although the longer-term benefits and harms are not known. Corticosteroid injections are being used increasingly in clinical practice alongside physiotherapy for the management of people with rotator cuff disorders; hence justification for investigating corticosteroid injection in the GRASP (Getting it Right: Addressing Shoulder Pain) trial alongside physiotherapist-prescribed exercise.”^114^

#### Explanation

Typically, the introduction of the trial report consists of free-flowing text, in which authors explain the scientific background and rationale for their trial, and its general outline. The rationale may be explanatory (eg, to assess the possible influence of a drug on renal function under tightly regulated conditions) or pragmatic (eg, to guide practice by comparing the benefits and harms of two treatments in a clinical setting). Authors should report any evidence of the benefits and harms of active interventions included in a trial and should suggest a plausible explanation for how the interventions might work, if this is not obvious. Understanding the rationale or theory underpinning an intervention helps readers to understand which aspects or components are likely to be essential to its efficacy.^23^ In addition, authors should justify the choice of comparator(s).^115^ ^116^ The choice of the comparator (active or placebo) will influence effect estimates. It could raise ethical concerns if patients could be allocated to a placebo or to a suboptimal treatment while an active treatment has been proven effective. Authors should justify the need for the trial they conducted and show that there was equipoise about the best treatment for the condition in the population being studied.

The Declaration of Helsinki states that biomedical research involving people should be based on a thorough knowledge of the scientific literature.^117^ It is unethical to expose humans unnecessarily to the risks of research. Some clinical trials have been shown to have been unnecessary because the question they addressed had been, or could have been, answered by a systematic review of the existing literature.^118^ ^119^ ^120^ Thus, the need for a new trial should be justified in the introduction. Ideally, this justification should include a reference to one or more systematic reviews of previous trials. In the absence of a published systematic review, authors should report and summarise the results of previous relevant trials or note their absence.^121^ The percentage of published trial reports that cite a systematic review of pre-existing evidence where one is available has increased over time, but over a quarter still fail to do so.^122^

### Item 7: Specific objectives related to benefits and harms

#### Example

“To evaluate whether a structured exercise programme improved functional and health related quality of life outcomes compared with usual care for women at high risk of upper limb disability after breast cancer surgery.”^123^

#### Explanation

Objectives are the questions that the trial was designed to answer. Adequate reporting of the research question is essential to allow readers to appraise and interpret the trial results. The PICO framework, which requires defining the patient population (P); the experimental intervention (I); the comparator intervention or condition (C); and the outcome or outcomes (O) of interest, has been proposed to help define the research question. PICO is sometimes styled as PICOTS, to include T (the timeframe) and/or S (the setting).^124^

Treatment decisions require an evaluation of the balance between benefit and harm; however, information about harms is frequently omitted or incompletely reported in published reports of trial results.^125^ ^126^ ^127^ ^128^ Trials whose primary objective is to evaluate benefits of an intervention may not be powered to detect harms, but authors should still report whether they considered harms outcomes when planning the trial.^20^

Authors should clarify whether the aim is to establish superiority of the experimental intervention, or non-inferiority or equivalence, as compared with the comparator intervention.^129^ Authors should also report whether the trial is intended to provide preliminary data (a pilot or feasibility trial),^130^ explore pharmacokinetic properties, or generate confirmatory results.

For multi-arm trials, authors should clarify which treatment group comparisons are of interest (eg, A *v* B; A *v* C). If authors planned to readjust the objective during the trial (eg, in some platform trials or basket trials^131^), this should be reported. Finally, trials can be designed to study the effect of the experimental intervention under different conditions, often described on a spectrum from ideal conditions (explanatory trial) to standard clinical care conditions (pragmatic trial).^132^

The objectives should be phrased using neutral wording (eg, “to compare the effect of treatment A versus treatment B on outcome X for persons with condition Y”) rather than in terms of a particular direction of effect.^133^ The trial objectives should align with what was specified in the trial registry and protocol; any changes to the trial objectives after it commenced should be reported with reasons (item 10).

Recently, some trials have been designed using the estimands framework to define the research question and trial objectives. While the terminology surrounding estimands may be new to some investigators, it is expected that the use of this framework will become more widespread. Box 1 provides more information about the estimands framework and how it is being used.

Box 1 **Estimands**

Concerns have been raised that the precise research questions that randomised trials set out to answer are often unclear.^134^ In particular, there is often ambiguity around how events occurring after randomisation (termed intercurrent events) are handled. Specifying the research question using an estimands framework is increasingly used to improve clarity. Despite calls for estimands to be included in the CONSORT 2025 statement,^134^ ^135^ their inclusion did not reach consensus. However, we provide a brief overview of estimands and introduce terminology, so they can be applied and reported if used. A more detailed primer on the estimand framework which provides practical guidance on estimands in studies of healthcare interventions can be found elsewhere.^135^

ICH E9(R1) defines an estimand as “a precise description of the treatment effect reflecting the clinical question posed by a given clinical trial objective.”^136^ The estimands framework provides a structured description of the objectives in an attempt to bring clarity in specifying the research question, which can be used to guide the study design, data collection, and statistical analysis methods. In brief, an estimand comprises five key attributes: population, treatment groups, endpoint, summary measure, and handling of intercurrent events (table 3). A separate estimand should be defined for each study outcome, and for some outcomes, more than one estimand may be defined.

**Table 3:** Five key attributes of the estimand framework^135^

Attribute	Definition
Population	Patients for whom researchers want to estimate the treatment effect
Treatment groups	Different intervention strategies being compared in the treatment effect definition
Endpoint	Outcome for each participant that is used in the treatment effect definition
Summary measure	Method used to summarise and compare the endpoint between treatment conditions (eg, risk ratio, odds ratio)
Handling of intercurrent events	Strategies used to handle each intercurrent event* in the treatment effect definition; different strategies could be used for different types of intercurrent events

* Intercurrent events are post-baseline events (or post-randomisation events in randomised trials) that affect the interpretation or existence of outcome data. These events frequently affect receipt of treatment (eg, treatment switching or treatment discontinuation) or preclude existence of the outcome (eg, death, if it is not defined as part of the outcome).

The ICH E9(R1) outlines five strategies for handling intercurrent events, which are at the core of the estimand framework (table 4).

**Table 4:** Strategies for handling intercurrent events

Strategy	Description
Treatment policy	The occurrence of the intercurrent event is considered irrelevant in defining the treatment effect of interest: the value for the outcome of interest is used regardless of whether the intercurrent event occurs
Hypothetical	The treatment effect in a scenario where the intercurrent event did not occur is of interest
Composite	The intercurrent event is incorporated into the outcome definition
While on treatment	The outcome before the occurrence of the intercurrent event is of interest
Principal stratum	The outcome in a subpopulation of patients who would not (or would) experience the intercurrent event is of interest.

Although the terminology surrounding estimands may be new to some investigators, it is expected that defining research questions using the estimands framework will become more widespread. A number of existing reporting guidelines have recently included estimands with the reporting recommendations.^137^ ^138^ ^139^ If the estimands framework has been used to design the trial or the data collection or to inform the statistical analysis (by guiding choice of appropriate methods), then this should be made clear in the manuscript and the methods and results should be reported within the framework.

## CONSORT 2025: Methods

### Item 8: Details of patient or public involvement in the design, conduct, and reporting of the trial

#### Examples

“A study patient advisory group advised on study design before funding, in study set-up, and during recruitment. They chose the term best current treatment and informed the design of clinic procedures (including how best to reduce the burden of intervention), questionnaire design, and participant information. This group informed protocol modifications in response to low recruitment, and guided interpretation of the findings. Two public contributors were members of the independent trial steering committee.”^140^

“The UK Musculoskeletal Trauma Patient and Public Involvement (PPI) Group co-designed this study. In particular, the group advised on the choice of outcome measures and the follow-up arrangements, which were designed to limit the number of face-to-face hospital visits needed. Subsequently, a patient member from this group became a member of the DRAFFT2 Trial Management Group, overseeing all elements of the set-up and delivery of the trial and the dissemination of the lay summary at completion. Another patient member of the group was also a member of the independent Trial Steering Committee.”^141^

“Office workers, workplace champions, and managers within the target organisation were involved in the study design during the grant application process and the study delivery phase. During the grant application phase, the purpose and design of the study as well as the suggested intervention strategies were presented to two large groups of council employees. As a result of these meetings, the study design included using finger prick blood testing rather than taking venous blood samples, participants receiving feedback on health measures, and incentives for attending follow-up. During the study set-up and delivery, a council employee advisory group met several times and provided advice on delivery of the interventions (feedback showed that workplace champions would not be comfortable delivering the initial education session because of the training and planning time required, so this session was delivered online instead), recruitment processes (feedback was provided on participant documents and recruitment messages and strategies within the council), installation of the height adjustable desk, and troubleshooting. Two council employees were also part of the trial steering committee, which met twice a year during the study”.^142^

“In the context of the pandemic and the need to design the study in a short period, no patients were involved in setting the research questions or the outcome measures, nor were they involved in developing plans for recruitment, design, or implementation of the study. No patients were asked to advise on interpretation or writing up of results.”^143^

#### Explanation

Patient and public involvement (PPI) has been shown to be particularly beneficial in clinical trials.^144^ It can help researchers to identify and prioritise research topics and questions; identify relevant outcome measures; boost recruitment and retention; improve trial design and tools; and improve the acceptability of trials.^144^ ^145^ PPI can also improve the communication and dissemination of the trial results to participants. Public involvement in other types of health research has been shown to help researchers to engage under-served populations and recruit diverse participant groups.^144^ Thus, transparent reporting of PPI is essential to allow readers to appraise the relevance and usefulness of findings to end users and to fully evaluate and understand a trial’s methodology and conduct. If patients and the public were not involved, authors should report this with the reasons why.

PPI in health research entails collaborating or partnering with patients and members of the public to design, conduct, report, interpret, or disseminate research: the research is done by or with patients and the public—rather than done for, at, or about them.^146^ ^147^ ^148^ Importantly, this is distinct from including patients or members of the public in a trial as participants. PPI contributors can be people with current or past experience of a health condition; their families, carers, and advocates; members of communities who are target users of an intervention or service; or members of the wider public with a broader perspective. The terminology used differs internationally: for example, such activity is most commonly known as “patient and public involvement” in the UK, whereas “patient engagement” is more common in mainland Europe and North America, 147 148, “community and public engagement” is commonly used in part of Africa, and “consumer involvement” is frequently used in Australia.^149^

The GRIPP (Guidance for Reporting Involvement of Patients and Public) checklist was developed in 2011 with the aim of improving the reporting of PPI activities in health research^150^; followed in 2017 by GRIPP2.^151^ The GRIPP2 checklist includes GRIPP2-SF, which is a short form of the GRIPP2 checklist where PPI is the secondary focus of the research.^151^ Examples of reporting of PPI involvement could include whether and how patients were involved in the trial objectives, whether patients advised on optimising patient recruitment and retention, and whether and how patients were included in selection of the trial outcomes.

Funding bodies are increasingly encouraging or requiring researchers to include PPI in grant applications,^146^ ^152^ ^153^ but mandating of PPI reporting by journals has remained uncommon. In 2014, *The BMJ* introduced a requirement for submitted manuscripts to include a PPI statement,^154^ and this was extended to other BMJ journals from 2018.^155^ ^156^ A 2023 study of trials addressing chronic conditions found that approximately 80% of trial reports published in these journals included a PPI subsection, and around 40% of these reported that PPI activities had been conducted.^157^ Few other journals have followed suit in mandating reporting of PPI activities.^158^ ^159^ ^160^ In the absence of an explicit requirement, reporting of PPI activities remains infrequent, appearing in an estimated 0-5% of published trial reports.^161^ ^162^ ^163^ There is limited evidence to indicate the extent to which this reflects a lack of PPI activity versus PPI activity being conducted but not reported. In a small survey of authors of trials published in high impact factor journals, only one of 29 respondents reported PPI activities having been included but not mentioned in the published article^163^; while in a survey of authors of pragmatic trials, 47% reported including PPI activities during a trial but less than a quarter of these reported the activity in the corresponding publication.^164^

### Item 9: Description of trial design including type of trial (eg, parallel group, crossover), allocation ratio, and framework (eg, superiority, equivalence, non-inferiority, exploratory)

#### Examples

“This was a multicenter, stratified (6 to 11 years and 12 to 17 years of age), with imbalanced randomization [2:1], double-blind, placebo-controlled, parallel-group study conducted in the United States (41 sites).”^165^

“This multicentre, pragmatic, superiority randomised trial compared three parallel groups for patients referred to secondary care for treatment of primary frozen shoulder, who were recruited from 35 hospital sites in the UK. Individual participants were randomly assigned with unequal allocation (2:2:1) to arthroscopic capsular release, manipulation under anaesthesia, or early structured physiotherapy, to allow for different effect sizes between groups.”^80^

#### Explanation

The word “design” is often used to refer to all aspects of how a trial is set up, but it also has a narrower interpretation. Many aspects of the broader trial design, including details of randomisation and blinding, are addressed elsewhere in the CONSORT checklist. This item refers to the type of trial (eg, parallel group or crossover) and the conceptual framework (eg, superiority, equivalence, or non-inferiority).

CONSORT 2025 focuses mainly on trials with participants individually randomised to one of two parallel groups. While many published trials have such a design, the main alternative designs are multi-arm parallel,^166^ crossover,^167^ cluster,^168^ and factorial designs,^138^ with each having their own specific CONSORT extensions. A detailed review of randomised trials published in PubMed in 2012 showed that of the 1122 trials, 953 (85%) were parallel group; the other main designs were crossover (n=98; 13%) and cluster (n=31; 3%). Of these 1122 trials, 892 (80%) had two groups, 146 (13%) had three groups, and 61 (5%) had four or more groups.^169^

Most trials are designed to identify the superiority of a new intervention, if it exists, but others are designed to assess non-inferiority or equivalence.^129^ It is important that researchers clearly describe the design of their trial, including the unit of randomisation (eg, patient, general practice, lesion). These details should also be included in the abstract (item 1b).

If a less common design is used, authors should explain their choice, especially as such designs may imply the need for a larger sample size or more complex analysis and interpretation. Although most trials use equal randomisation (eg, 1:1 for two groups), it is also helpful to provide the allocation ratio explicitly.

### Item 10: Important changes to the trial after it commenced, including any outcomes or analyses that were not prespecified, with reason

#### Examples

“The original primary endpoint was all-cause mortality, but, during a masked analysis, the data and safety monitoring board noted that overall mortality was lower than had been predicted and that the study could not be completed with the sample size and power originally planned. The steering committee therefore decided to adopt co-primary endpoints of all-cause mortality (the original primary endpoint), together with all-cause mortality or cardiovascular hospital admissions (the first prespecified secondary endpoint).”^170^

“As described in the published protocol paper, a protocol amendment was made to revise the sample size in response to new information on the minimal clinically important difference of the primary outcome measure, the Patient-Oriented Eczema Measure (POEM). Our original sample size used a POEM score for minimal clinically important difference of 3, which was based on research carried out in secondary care among people with moderate or severe eczema. Fresh evidence, however, suggested that a change in POEM score of 2.1 to 2.9 represents a change likely to be beyond measurement error. A protocol amendment was therefore made to change the target sample size for the trials based on seeking to detect a difference in POEM score of 2.5 points between groups, increasing the target sample size from 200 to 303 for each trial.”^171^

#### Explanation

A protocol for a randomised trial serves as the foundation for planning, conduct, reporting, and appraisal, specifying in detail how the trial will be conducted. There may be deviations from the original protocol, as it is impossible to predict every possible change in circumstances during the course of a trial. Some trials will therefore have important changes to the methods after trial commencement. There are many reasons for deviations from the initial study protocol; for example, changes could be due to external information becoming available from other studies, or internal financial difficulties, or lower than anticipated recruitment rates. In some trials, an independent data monitoring committee will have as part of its remit the possibility of recommending protocol changes based on seeing unblinded data. Authors should report the nature and timing of protocol changes because changes made at different times (eg, before or after breaking the blinding) might be associated with different risks of bias.

Authors should report all major changes to the trial after it commenced indicating the reason for the changes and when the changes occurred.^172^ Such changes might affect the trial methods, such as the randomisation ratio, eligibility criteria, interventions, outcomes, method of analysis or duration of follow-up; or might affect the trial conduct, such as dropping a trial site with poor data quality.^173^

Some trials are set up with a formal adaptive design, which allows pre-planned changes to an ongoing trial without compromising the validity of conclusions.^174^ It is therefore essential to distinguish pre-planned changes from unplanned changes that may also occur. Such adaptive trial design modifications are usually to the sample size and number of treatment groups, and can lead to decisions being made more quickly and with more efficient use of resources than would be possible with traditional, non-adaptive parallel group trials. Specific guidance has been developed for reporting trials with a formal adaptive design; authors could consult this for more detailed information.^174^

Most trials record multiple outcomes, with the risk that results will be reported for only a selected subset. Prespecification and reporting of completely defined primary and secondary outcomes for both benefits and harms (item 14) should remove such a risk. In some trials, however, circumstances require a change in the way an outcome is assessed, the designation of outcomes as primary or secondary or even, as in the example above, a switch to a different outcome. For example, there may be external evidence from other trials or systematic reviews suggesting the time point for the primary outcome might not be appropriate; or recruitment or the overall event rate in the trial may be lower than expected.^170^ Changing an endpoint based on unblinded data are much more problematic, although may be specified in the context of an adaptive trial design.^174^

Whether the modifications are explicitly part of the trial design or in response to changing circumstances, it is essential that they are fully reported and the reason for the change explained to help the reader interpret the results. Such information is not always reported. A comparison of protocols and publications of 102 randomised trials found that 62% of trial reports had at least one primary outcome that was changed, introduced, or omitted compared with the protocol.^51^ Primary outcomes also differed between protocols and publications for 40% of a cohort of 48 trials funded by the Canadian Institutes of Health Research.^175^ None of these subsequent 150 trial reports mentioned, let alone explained, changes from the protocol. Similar results from other studies have been reported in a systematic review of empirical studies, comparing trial registers or protocols to published trial reports.^176^

### Item 11: Settings (eg, community, hospital) and locations (eg, countries, sites) where the trial was conducted

#### Examples

“The study was conducted in paediatric diabetes services experienced in the use of CSII [continuous subcutaneous insulin infusion], in nine University and six local hospitals within the NHS in England and Wales.”^177^

“Setting: One large health board area with a materially deprived, inner city population in the west of Scotland, United Kingdom. Although described as a single centre, NHS Greater Glasgow and Clyde has the largest health board population (1.2m) in the United Kingdom. It is spread over a wide geographical area including severely materially deprived post-industrial areas as well as some more affluent communities. Maternity booking and antenatal care are provided in both hospital and local healthcare settings. Delivery (13 844 infants in 2013[reference]) takes place in three major maternity hospitals.”^178^

#### Explanation

Along with the eligibility criteria for participants (item 12a) and the description of the interventions (item 13), information on the settings and locations where the trial was conducted is crucial to enable readers to judge the applicability and generalisability of a trial. Were participants recruited from primary, secondary, or tertiary healthcare, or from the community? Healthcare institutions vary greatly in their organisation, experience, and resources and the baseline risk for the condition under investigation. Other aspects of the setting, including the social, economic, and cultural environment, might also affect a study’s external validity.

Authors should report the number and type of settings and describe the care providers involved. They should report the locations in which the study was carried out, including the country, city if applicable, and immediate environment (eg, community, general practice, hospital outpatient clinic, or inpatient unit). In particular, it should be reported whether the trial was carried out in one site (single centre trials) or several sites (multicentre trials). The description of the setting should provide enough information so that readers can judge whether the results of the trial could be relevant to their own setting. The environment in which the trial is conducted might differ considerably from the setting in which the trial’s results are later used to guide practice and policy.^179^ ^180^ Authors should also report any other information about the settings and locations that could have influenced the observed results.

### Item 12a: Eligibility criteria for participants

#### Example

“Patients aged 18 years or older were recruited from 20 UK National Health Service (NHS) trusts. Patients were eligible if they had a diagnosis of shoulder pain attributable to a rotator cuff disorder (eg, cuff tendonitis, impingement syndrome, tendinopathy, or rotator cuff tear) that had started within the past 6 months. We used the diagnostic criteria set out in the British Elbow and Shoulder Society (BESS) guidelines. Patients were excluded if they had a history of significant shoulder trauma (eg, dislocation, fracture, or full-thickness tear requiring surgery), neurological disease affecting the shoulder, other shoulder conditions (eg, inflammatory arthritis, frozen shoulder, or glenohumeral joint instability), received corticosteroid injection or physiotherapy for shoulder pain in the past 6 months, or were being considered for surgery. Detailed criteria are in the protocol.”^181^

#### Explanation

A comprehensive description of the eligibility criteria used to select the trial participants is needed to help readers interpret the study. In particular, all inclusion and exclusion criteria should be reported to judge to whom the results of a trial apply—that is, the trial’s generalisability (applicability) and relevance to clinical or public health practice (item 30).^179^ A description of the method of recruitment, such as by referral or self-selection (eg, through advertisements) is also important in this context. Because they are applied before randomisation, eligibility criteria do not affect the internal validity of a trial, but they are central to its external validity.

Typical and widely accepted selection criteria relate to the nature and stage of the condition or disease being studied, the exclusion of persons thought to be particularly vulnerable to harm from the study intervention, and to issues required to ensure that the study satisfies legal and ethical norms. The informed consent of study participants, for example, is typically required in intervention studies. Where relevant, it is important to describe whether sex and/or gender were taken into account in the design of the trial, including whether there was adequate representation of men and women (or diverse genders), and justify the reasons for any exclusion.^182^

Despite their importance, eligibility criteria are often not reported adequately. For example, in an analysis of 283 reports of trials published between 1994 and 2006 in high impact general medical journals, reporting of exclusion criteria was often poor and incomplete: 84% of published trials contained at least one poorly justified exclusion criteria, and in 61% more than a quarter of the trial’s exclusion criteria were poorly justified.^183^ Any differences in eligibility criteria between the trial protocol and final publication should also be highlighted and reasons for any discrepancies reported. A review of 52 protocols and 75 subsequent full publications submitted to a German medical ethics committee between 2000 and 2006 identified modifications to the eligibility criteria for 85% of trial publications, with 41% of final publications containing newly added criteria.^184^ Similar deficiencies have been found in other studies.^185^ ^186^

### Item 12b: If applicable, eligibility criteria for sites and for individuals delivering the interventions (eg, surgeons, physiotherapists)

#### Example

“All participating centres . . . were major neurosurgical centres, treating large numbers of patients after aneurysmal subarachnoid haemorrhage (SAH), each centre treating between 60 and 200 cases annually . . . Centres had to have expertise in both neurosurgical and endovascular management of ruptured aneurysms. Only accredited neurosurgeons with experience of aneurysm surgery were permitted to manage patients in the trial. Endovascular operators had to have done a minimum of 30 aneurysm treatment procedures, before they were permitted to treat patients in the trial”.^187^

#### Explanation

For all types of trials, it is important to define the eligibility criteria for clinical sites, and to consider the characteristics of the treatment providers who will provide both the experimental and comparator interventions.^22^ ^188^ Evidence suggests that patient outcomes can be associated with hospital and care provider volume.^188^ A systematic review of 135 trials found that 71% observed a positive association between hospital volume and outcomes, and 70% observed an association between care provider volume and outcomes.^189^ Different levels of expertise of care providers in each intervention group can bias treatment effect estimates.^190^ Furthermore, an intervention might be found to be safe and effective in a trial performed in high volume sites by high volume care providers but could have different results in low volume sites. For example, in an analysis of Medicare National Claim files of 167 208 patients who had undergone coronary stent surgery, patients treated by high volume physicians and at high volume sites experienced better outcomes than those treated by low volume physicians at low volume sites.^191^ Similar studies show that in most non-pharmacological trials, care provider expertise and site volume will influence treatment effects.^192^ ^193^ ^194^

Eligibility criteria for care providers and sites are often poorly reported. A systematic review of randomised trials in surgery found that the setting and the site volume of activity were reported in only 7% and 3% of articles, respectively. Eligibility criteria were reported for care providers in 41% of the articles, and the number of care providers performing the intervention was reported in 32%.^195^ A careful description of care providers involved in the trial, as well as details of the sites in which participants were treated, helps readers appraise the risk of bias and the applicability of the results.^22^ ^188^ Eligibility criteria for sites typically relate to site volume for the procedure under investigation or similar procedures. Eligibility criteria for care providers might include professional qualifications, years in practice, number of interventions performed, skill as assessed by level of complication when performing the intervention, and specific training before trial initiation. Exclusion criteria should be justified because they will influence the applicability of the trial results.^188^

### Item 13: Intervention and comparator with sufficient details to allow replication. If relevant, where additional materials describing the intervention and comparator (eg, intervention manual) can be accessed

#### Examples

“Each sulfadoxine–pyrimethamine course consisted of three tablets containing 500 mg of sulfadoxine and 25 mg of pyrimethamine (unbranded generic sulfadoxine–pyrimethamine, Medopharm, Chennai, India; quality controlled by Durbin, Hayes, UK) given as a single oral dose for 1 day (appendix p 2). Each dihydroartemisinin–piperaquine course was dosed according to the bodyweight of each participant and consisted of three to five tablets containing 40 mg of dihydroartemisinin and 320 mg of piperaquine (Alfasigma, Bologna, Italy), given orally once a day for 3 consecutive days. Each dose of azithromycin consisted of two tablets containing 500 mg (Universal Corporation, Nairobi, Kenya) given orally once daily for 2 consecutive days (cumulative dose of 2 g) at the same time as the first and second daily dose of dihydroartemisinin–piperaquine at enrolment. The placebo tablets were also provided by Universal Corporation and had the same appearance as active azithromycin (appendix p 2). The first daily dose was administered in the study clinic under the direct supervision of the study staff, combined with a slice of dry bread or a biscuit. The daily doses on the second and third days were self-administered at home at approximately the same time of the day and in a similar manner as the first dose taken under observation in the clinic.”^196^

“The experimental group received 6 sessions of standard OMT (osteopathic manipulative treatment), and the control group 6 sessions of sham OMT, each session at 2-week intervals. For both experimental and control groups, each session lasted 45 minutes and consisted of 3 periods: (1) interview focusing on pain location, (2) full osteopathic examination, and (3) intervention consisting of standard or sham OMT. Briefly, in both groups, practitioners assessed 7 anatomical regions for dysfunction (lumbar spine, root of mesentery, diaphragm, and atlantooccipital, sacroiliac, temporomandibular, and talocrural joints) and applied sham OMT to all areas or standard OMT to those that were considered dysfunctional. All health care providers were board-certified nonphysician, nonphysiotherapist osteopathic practitioners (Répertoire National de la Certification Professionnelle, niveau 1). They all received a 2-day training according to international standards to deliver both standard and sham OMT. Full descriptions of osteopathic practitioner training and interventions are provided in eAppendices 3 and 4 in Supplement 2. In both groups, pharmacological interventions, nonpharmacological interventions, and spinal surgery were allowed. Cointerventions were self-reported at 3, 6, and 12 months by use of a standardized checklist (eAppendix 5 in Supplement 2).”^197^

#### Explanation

Complete reporting of the intervention and comparator details is essential to enable readers to understand the study results and adequately translate them to clinical practice. Several studies have shown poor reporting of interventions and comparators in randomised trials.^195^ ^198^ ^199^ ^200^ ^201^ ^202^ ^203^ ^204^ ^205^ ^206^ Authors should describe each intervention thoroughly, including control interventions, or use of placebo procedure.^207^ ^208^ The description should provide sufficient detail to allow replication, such as to allow a clinician wanting to use the intervention to know exactly how to administer the intervention/comparator that was evaluated in the trial.^198^ Key information includes the different components of the intervention/comparator, how and when it should be administered, the intervention/comparator material (ie, any physical or informational materials used in the intervention/comparator, including those provided to participants or used in intervention/comparator delivery or in training of providers and where it can be accessed (eg, online appendix, URL)); the procedure for tailoring the intervention/comparator to individual participants, and how fidelity^209^ (ie, the extent to which the intervention/comparator were implemented as planned in the protocol by care providers) or adherence^209^ (ie, the extent to which trial participants implement the intervention/comparator as planned in the protocol) were assessed or enhanced (box 2).

Box 2 Examples of essential information to be reported for various types of interventions in randomised trials*^22^ ^23^

**Drug^23^**

- Generic name
- Manufacturer
- Dose
- Route of administration (eg, oral, intravenous)
- Timing
- Titration regimen if applicable
- Duration of administration
- Procedure for tailoring the intervention to individual participants
- Conditions under which interventions are withheld
- Whether and how adherence of patients to the intervention was assessed or enhanced
- Any physical or informational materials used in the intervention and where the materials can be accessed.

**Rehabilitation, behavioural treatment, education, and psychotherapy, etc^22^ ^23^**

- Qualitative information
  - Theory/rationale for essential intervention elements
  - Content of each session
  - Mode of delivery (individual/group, face to face/remote)
  - Whether the treatment is supervised
  - The content of the information exchanged with participants
  - The materials used to give information
  - Procedure for tailoring the intervention to individual participants
  - Whether and how the interventions were standardised
  - Background and expertise of individuals delivering the interventions
  - Whether the same care providers delivered interventions across trial groups
  - Whether and how adherence of individuals delivering the interventions to the protocol was assessed or enhanced
  - Whether and how adherence of patients to the intervention protocol was assessed and/or enhanced
  - Any physical or informational materials used in the intervention and where the materials can be accessed.
- Quantitative information
  - Intensity of the intervention where appropriate
  - Number of sessions
  - Session schedule
  - Session duration
  - Duration of each main component of each session
  - Overall duration of the intervention.

**Surgery, technical procedure, or implantable device^22^**

- Preoperative care relevant details
- Intraoperative care relevant details
- Configuration of any device
- Postoperative care relevant details
- Procedure for tailoring the intervention to individual participants
- Whether and how the interventions were standardised
- Background and expertise of individuals delivering the interventions
- Whether the same care providers delivered interventions across trial groups
- Whether and how adherence of individuals delivering the interventions to the protocol was assessed or enhanced
- Any physical or informational materials used in the intervention and where the materials can be accessed.

*This list is not intended to be exhaustive; it is a starting point for authors to consider when reporting the intervention.

Assessing fidelity and adherence can be complex and vary according to the intervention/comparator (eg, one-off, short term repeated, long term repeated). Various deviations to the protocol can occur. For example, participants might initiate the intervention but then discontinue the intervention completely and permanently after a specific period of time, discontinue the intervention temporarily, reduce the dose, or modify the schedule. If relevant, authors should provide the prespecified definition for classifying participants as being treated as planned or not.

In addition, authors should indicate whether criteria were used to guide intervention/comparator modifications and discontinuations and where applicable describe these criteria. This information could be particularly important to evaluate the risk of bias due to deviations from the intended interventions,^210^ ^211^ an important domain of the risk-of-bias tool developed by Cochrane.^210^ Assessing this domain requires a clear understanding of, and ability to distinguish between, deviations that occur as planned in the protocol and deviations that arise due to the experimental context.

The research question (ie, explanatory *v* pragmatic) will affect the standardisation of the intervention/comparator as well as how adherence or fidelity is assessed or enhanced. In explanatory trials, the aim is to estimate treatment effect under ideal circumstances. The intervention/comparator are usually highly standardised with close monitoring of fidelity and adherence to the intervention/comparator and strategies to increase them. In contrast, pragmatic trials aim to determine treatment effect in clinical conditions. The intervention and comparator are usually highly flexible, and measurement of fidelity and adherence are unobstructive with no strategies to maintain or improve them. Nevertheless, assessing fidelity and adherence to the intervention/comparator, or at least recording the most important components of the intervention/comparator, is necessary to understand what was actually administered to participants. This is particularly important for complex interventions where diversity in the implementation of the intervention is expected. For example, in a pragmatic trial assessing a surgical procedure where the procedure is left to surgeons’ choice, investigators should plan to systematically record key elements related to pre-operative care, anaesthesia, the surgical approach, and post-operative care. This information is essential to provide a relevant description of the intervention that was actually provided when the trial is completed.

If the control group or intervention group received a combination of interventions, the authors should provide a thorough description of each intervention, an explanation of the order in which the combination of interventions were planned to be introduced or withdrawn, and the triggers for their introduction if applicable. Some complex interventions will require the development of specific documentation (eg, training materials, intervention manuals). Authors should make these available and indicate where they can be accessed.

If the control group is to receive usual care, it is important to describe what that constitutes so that readers can assess whether the comparator differs substantially from usual care in their own setting.^212^ Various approaches could be used: standardising usual care to be in line with specific guidelines; or asking practitioners to treat control patients according to their own preference, which could result in heterogeneity of the care provided particularly between centres and over time.^213^ Usual care can vary substantially across sites and patients, as well as over the duration of the trial. Further, it is important to clarify if the experimental group also received usual care in addition to the experimental intervention, and what actually differed between the groups. Usual care is frequently incompletely reported. In a review of 214 paediatric trials, the descriptions of standard of care were more often incomplete than the description of the intervention arms within the same study as measured by the TIDieR checklist (mean 5.81 (standard deviation (SD) 2.13) *v* 8.45 (SD 1.39)).^205^

If the control group is to receive a placebo, specific considerations need to be accounted for. Some evidence showed that placebos are insufficiently described.^214^ Placebo could have several different aspects from pills to saline injections or more complex interventions such as sham interventions (eg, sham surgery) or attention control interventions. Authors should report the same level of details as required for the intervention—that is, content of the placebo or different component of the placebo, how and when it should be administered, material, procedure for tailoring the placebo to individual participants, and how fidelity and adherence were assessed or enhanced.^207^ Complete reporting of the placebo is needed to understand what intervention effect is measured in the trial.^215^ A network meta-analysis of osteoarthritis trials showed that different placebo interventions (oral, intra-articular, topical, oral and topical) had different effects and can impact the relative effect estimate of active treatments.^216^

Further, the trial groups could receive different concomitant care in addition to the assigned trial interventions. Concomitant care could impact trial outcomes and bias effect estimates. To facilitate interpretation of study results and risk-of-bias assessments, authors should report relevant concomitant care that was allowed or prohibited where relevant.

Specific guidance has been developed to improve the reporting of interventions, particularly TIDieR,^23^ TIDieR-Placebo for placebo and sham controls,^207^ and the CONSORT extensions for non-pharmacological treatments.^22^ Authors could consult these for more detailed information.

### Item 14: Prespecified primary and secondary outcomes, including the specific measurement variable (eg, systolic blood pressure), analysis metric (eg, change from baseline, final value, time to event), method of aggregation (eg, median, proportion), and time point for each outcome

#### Example

See table 5.^21^

**Table 5:** Five core elements of a defined outcome with examples

Element term	Definition used	Example 1	Example 2	Example 3
Domain	Title or concept to describe one or more outcomes	Blood pressure	Depression	Death
Measurement variable or specific measurement	Corresponds to the data collected directly from the trial participants; description includes the instrument used to assess the outcome domain	—	—	—
	• Descriptive name	Systolic blood pressure measured with Omran upper arm blood pressure monitor	MADRS	All cause mortality, per hospital database
	• If applicable, the total score or subscale that will be analysed	Not applicable	MADRS total score	Not applicable
Specific metric	Participant level unit of measurement (eg, change from baseline, final value or a value at a time point, time to event) for the analysis	Value at a time point	Change from baseline	Time to event
Method of aggregation	Procedure for estimating the treatment effect	—	—	—
	• If the outcome will be treated as continuous, categorical, or, time to event variable	Continuous variable	Binary variable	Time to event
	• For continuous variables, a measure of central tendency (eg, mean value); for categorical and time-to-event data variables, proportion with an event, and (if relevant) the specific cut-off values or categories compared	Mean value	Proportion of participants with ≥50% decrease	Incidence density and between group incidence density rate
Time point	Timing of follow-up measurements	—	—	—
	• When outcome measurements will be obtained	2, 4, and 12 weeks after randomisation	2, 4, 6, and 8 weeks after randomisation	Daily
	• Which of the outcome measurements will be analysed	12 weeks after randomisation	8 weeks after randomisation	End of follow-up

MADRS=Montgomery-Asberg Depression Rating Scale. Table adapted from Butcher et al.^21^

#### Explanation

All randomised trials assess outcomes, for which the groups are compared. Most trials have several outcomes, some of which are of more importance than others. The primary outcome is the prespecified outcome considered to be of greatest importance to relevant stakeholders (such as patients, policy makers, clinicians, and funders) and should be the one used in the sample size calculation (item 16). The primary outcome should be explicitly indicated as such in the report of a randomised trial. Other outcomes of interest are secondary outcomes.

It is important to explain the rationale and clinical relevance for chosen efficacy and harm outcomes, including whether they are part of a core outcome set.^217^ ^218^ A core outcome set is an agreed standardised set of outcomes that should be measured and reported, as a minimum, in all clinical trials in specific areas of health or health care. The COMET (Core Outcome Measures in Effectiveness Trials) initiative and COMET database facilitate access to core outcome sets (https://www.comet-initiative.org/).

Most trials have a single primary outcome. Having several primary outcomes can incur potential problems of interpretation associated with multiplicity of analyses (items 28 and 30). There are typically multiple secondary outcomes (ie, the outcomes prespecified in the trial protocol to assess any additional effects of the intervention). Secondary outcomes can include harms that may include unintended effects of the intervention (item 27).

The primary and secondary outcomes reported should be consistent with the outcomes prespecified in the trial protocol and the registry. Evidence shows important discrepancies between the outcome reported in the registry or protocol and outcomes reported in final publications, frequently in favour of statistically significant results. Any change in outcome(s) specified in the protocol should be reported, with reasons (item 10).^51^ ^175^ ^176^

All outcomes, whether primary or secondary, should be described and completely defined. This information is typically also detailed in the trial’s protocol and the trial registry. The principle here is that the information provided should be sufficient to allow others to use the same outcomes.^198^ For each outcome, it is important to detail: (1) the specific measurement variable, which corresponds to the data collected directly from trial participants (eg, Beck Depression Scale; all cause mortality) with definition where relevant (eg, major bleeding was defined as fatal bleeding or symptomatic bleeding in a critical area or organ; all cause mortality per hospital database); (2) the specific participant level analysis metric, which corresponds to the format of the outcome data that was used from each trial participant for analysis (eg, change from baseline; final value or value at a time point; time to event); (3) the method of aggregation, which refers to the summary measure format for each trial group (eg, mean; proportion of participants with score >2); and (4) the measurement time point of interest for analysis.^219^ For composite outcomes, all individual components of the composite outcome should be described as secondary outcomes.^21^ Only half of randomised trials published in PubMed indexed journals in 2000 and 2006 specified the primary outcome.^220^ ^221^ In recent samples of trials published in specific fields, reporting has improved but still two thirds did not provide a complete definition.^222^ ^223^

The use of previously developed and validated scales can help to enhance quality of measurement.^224^ ^225^ For example, assessment of health related quality of life using a validated instrument is critical to the integrity and applicability of the trial.^226^ Authors should report measurement properties of outcome measurement instruments to assist in interpretation and comparison with similar studies.^227^

In most trials, information on outcomes is set to be collected as part of the trial conduct. However, some trials may use existing data collecting structures (eg, national, healthcare or administrative registries). This should be clarified in the methods. There is empirical evidence that treatment effect estimates may be different in trials where outcomes are obtained from routinely collected data.^228^

### Item 15: How harms were defined and assessed (eg, systematically, non-systematically)

#### Examples

“Adverse events (AE) were assessed clinically and analytically at each monthly follow-up visit. The severity of AE were classified according to the National Cancer Institute Common Toxicity Criteria version 4.0. Following the onset of the first cases, the criteria for considering the presence of tenosynovitis were established as spontaneous pain that increased with movement in any tendon insertion with tenderness at that level and observation of localized inflammatory signs of at least 72 hours in duration . . . Patients were considered to have hepatotoxicity when they presented alanine transaminase, aspartate aminotransferase, or bilirubin elevations more than 2 times the upper limit of the normal range. Toxicity was considered severe, and therefore the drug was discontinued when symptomatic elevations were more than 3 times or asymptomatic elevations were more than 5 times the normal levels. All AE were recorded and additional information was required in case of serious adverse events.”^229^

“Immediate adverse events were assessed by monitoring participants 30 min after injection in the trial centre. All participants were required to report all local and systemic adverse reactions and adverse events after the injection using the trial’s mobile application. Solicited adverse reactions were defined as any events that occurred from day zero to day seven after each injection. Unsolicited adverse reactions were defined as any adverse reactions which occurred from day eight to day 28 after each injection. The severity of adverse reactions was defined using the Food and Drug Administration guidance for industry and toxicity grading scale for healthy adult and adolescent volunteers enrolled in preventive vaccine clinical trial.”^230^

#### Explanation

Evaluation and reporting of harms in randomised trials can be useful to inform decision makers on the benefit-risk balance of an intervention.^20^ ^36^ Randomised trials usually lack power and sufficient follow-up to adequately estimate harms^231^; nevertheless, they can provide data about harms that can be synthesised in meta-analyses if adequately reported.^20^ ^36^ For example, the Women’s Health Initiative trials on hormone therapy provided important data on the cardiovascular risk of hormone replacement therapy.^232^

Harm relates to the unwanted effects of an intervention. According to the specific context, a given event could be considered for assessing harm (eg, myocardial infarction in a trial assessing non-steroidal anti-inflammatory drugs in patients with osteoarthritis) or benefit (eg, myocardial infarction in a trial assessing aspirin in patients with cardiovascular risk factors) of an intervention.^232^ The use of the term “harms” is preferred over “safety” to better reflect the negative effect of interventions.^20^

Despite the importance of having access to data on harms, reporting of this information is poor.^127^ ^128^ ^233^ ^234^ A review of 184 drug trials published between 2015 and 2016 in four medical journals with high impact factors showed that 28% did not provide any details on how harm data were collected and 89% did not report who decided whether the harm was attributable to the study drug.^128^ An overview of 13 reviews assessing the reporting of harms in randomised trials using the 2004 CONSORT extension for harms showed that only 40% of the included trials addressed harms outcomes with definitions for each and 44% clarified how harms-related information was collected.^127^

How harms are defined and assessed will affect the results and effect estimates. Harm can be prespecified or not. They can be systematically assessed or rely on spontaneous declaration (ie, non-systematically assessed). To increase the study’s power, harm can be aggregated in a composite outcome (eg, cardiovascular diseases). Some trials implement a procedure to determine whether harms could be attributed to the intervention (ie, causality).

For each systematically assessed harm, authors should report the definition, the measurement variable (eg, name of a validated questionnaire), and where appropriate, the analysis metric for each participant (eg, time to event), the summary measure for each trial group (eg, proportion), and the time point of interest for analysis. They should describe the procedures for harm assessment including who did the assessment, whether they were blinded to the treatment allocated, the assessment time points, and the overall time period for recording harms. For non-systematically assessed harms, authors should report the mode of data collection with the time point and overall time period for recording harms. Nevertheless, non-systematically assessed harms can be difficult to analyse and interpretation of the results should be viewed with caution. To overcome this issue, trialists can code and group events into specific categories. Nevertheless, the lack of standardisation in data collection could result in selective and incomplete reporting.^231^ Access to individual participant data may be needed to adequately synthesise this information.^231^ ^235^ ^236^

Where appropriate, the process for coding each harm and grading its severity should be described including who did the coding and severity grading, and whether they were blinded to the allocated trial group.

If harms outcomes are aggregated (eg, cardiovascular events, serious events, severe events, withdrawals due to harms, harms imputed to treatment), authors should describe the process for classifying harms, including the grouping system used (eg, grading system to define severity), who did the grouping, and whether they were blinded to the treatment allocated.^237^ ^238^

Box 3 summarises the essential information to be reported related to harms. More detailed information can be found in the CONSORT extension for harms, which was updated in 2022.^20^

Box 3 **Reporting harms in randomised controlled trials**

**Systematically assessed harms**

- Definition and instrument used (eg, name of a validated questionnaire)
- Analysis metric for each participant (eg, time to event); summary measure for each trial group (eg, proportion); time point of interest for analysis, where appropriate.

**Procedures for harm assessment, including:**

- Who did the assessment
- Whether the assessors were blinded to the allocated trial group
- Assessment time points and the overall time period for recording harms

**Non-systematically assessed harms**

- How data were collected
- Assessment time points and overall time period for recording harms

**Coding harms and grading severity**

Where appropriate, process for coding each harm and grading its severity, including:

- Who did the coding and severity grading, and whether they were blinded to the allocated trial group
- Which coding and severity grading systems were used, if any
- Assessment time points and overall time period for recording harms

**Grouping of harms**

For grouping of harms by body system, seriousness, severity, withdrawals (due to harms), and causality:

- Definitions of grouping categories
- Who did the grouping, and whether they were blinded to the allocated trial group.

### Item 16a: How sample size was determined, including all assumptions supporting the sample size calculation

#### Examples

“We expected an improvement in PFS [progression free survival], in favor of avelumab, with a hazard ratio (HR) of 0.58. Considering a fixed design with a 2-sided α risk of 5% and a power of 80%, 106 events (progression or death) are needed to demonstrate this difference based on the Schoenfeld method. With an estimated recruitment rate of 3 patients per month, a follow-up period for each patient of 24 months, and a percentage of patients lost to follow-up or not evaluable of 15%, 132 patients had to be randomized, and we planned to enroll a total of 66 patients per group.”^239^

“The target sample size was 300 (150 per arm) over a 3-and-a-half-year recruitment period. This was based on an assumed proportion of individuals with clinically meaningful improvement in VA [visual acuity] (>10 letters) of 55% in the standard care arm and a 19% increase in the adjunct group to 75%, with approximately 7% loss to follow-up, at least 90% power and two-sided 5% type 1 error.”^240^

“In order to detect a minimum clinically important difference (MCID) in mean volume of daily PA [physical activity] of 2.1 m g [milligravity] at 12 months, and assuming a standard deviation (SD) of 5.3 m *g*, power of 80%, and a statistical significance level of 5%, a total of 202 participants were required. Allowing for 20% loss to follow-up and 20% non-compliance of accelerometer/intervention attendance meant that at least 338 participants were required (169 per group). The value of 2.1 m *g* was chosen as it represents an increase in PA that is equivalent to walking at the threshold between light intensity and moderate intensity (for example, 4 km per hour) for 30 min per day or 10–15 min of brisk walking per day.”^241^

“Sample size was based on the primary outcome measure, HOS ADL [hip outcome score activities of daily living subscale] at eight months post-randomisation, and was calculated using a minimum clinically important difference between groups of 9 points. We estimated the standard deviation to be 14 points; however, summaries presented at a planned interim data monitoring meeting found that the standard deviation was 18 points. A revised calculation (significance level 5%, power 90%, loss to follow-up 20%) gave a sample size of 214 (107 participants in each group). The data monitoring committee approved the sample size increase from 120 to 214 participants.”^242^

#### Explanation

Sample size calculations are a key design component for a trial and need careful planning. Sample size calculations need to balance ethical and logistical considerations alongside medical and statistical considerations so that the scientific question can be reliably and precisely answered in a timely manner without unnecessarily exposing individuals to ineffective or harmful interventions. They are generally based on one primary outcome. A trial should therefore be sufficiently large to have a high probability (power) of identifying a clinically important difference of a prespecified size that meets a criterion of statistical significance, if such a difference exists. The magnitude of the effect has an inverse relationship with the sample size required for its detection; that is, larger sample sizes are needed to detect smaller differences. Moreover, the inverse relationship is not linear: very small differences require enormous sample sizes to have good power to detect.

All details on how the sample size was determined should be reported to allow replication (in principle). Elements of the sample size calculation that need to be specified are the primary outcome (and time point) on which the calculation was based (item 14); the anticipated values for the outcome in each trial group (which implies the clinically important target difference between the intervention groups) at a specific time point with rationale or provenance of all quantities, including any relevant citations; or continuous outcomes, the standard deviation of the measurements^243^; the statistical test; the α (type I error) value and whether it is two sided; the statistical power (or the β (type II error) value); and the resulting target sample size per trial group (box 4). Details should be given of any inflation of the sample size made for attrition or non-adherence during the study. Reference to any formulas or software packages used for the sample size calculation should all be reported. The reporting will have additional considerations for crossover trials,^167^ factorial trials,^138^ cluster trials,^168^ multi-arm trials,^166^ within-person trials,^245^ and non-inferiority and equivalence trials.^129^

Box 4 DELTA^2^ recommended reporting items for the sample size calculation of a randomised controlled trial with a superiority question*^244^

**Core items**

1. Primary outcome (and any other outcome on which the calculation is based). If a primary outcome is not used as the basis for the sample size calculation, state why
2. Statistical significance level and power
3. Express the target difference according to outcome type
  - Binary—state the target difference as an absolute or relative effect (or both), along with the intervention and control group proportions. If both an absolute and a relative difference are provided, clarify if either takes primacy in terms of the sample size calculation
  - Continuous—state the target mean difference on the natural scale, common standard deviation, and standardised effect size (mean difference divided by the standard deviation)
  - Time to event—state the target difference as an absolute or relative difference (or both); provide the control group event proportion, planned length of follow-up, intervention and control group survival distributions, and accrual time (if assumptions regarding these values are made). If both an absolute and relative difference are provided for a particular time point, clarify if either takes primacy in terms of the sample size calculation
4. Allocation ratio. If an unequal ratio is used, the reason for this should be stated
5. Sample size based on the assumptions as per above
  - Reference the formula/sample size calculation approach, if standard binary, continuous, or survival outcome formulas are not used. For a time-to-event outcome, the number of events required should be stated
  - If any adjustments (eg, allowance for loss to follow-up, multiple testing) that alter the required sample size are incorporated, they should also be specified, referenced, and justified along with the final sample size
  - For alternative designs, additional input should be stated and justified
  - Provide details of any assessment of the sensitivity of the sample size to the inputs used

**Additional items for grant application and trial protocol**

1. Underlying basis used for specifying the target difference (an important or realistic difference)
2. Explain the choice of target difference—specify and reference any formal method used or relevant previous research

**Additional item for trial results paper**

1. Reference the trial protocol.

*Taken from Cook et al.^244^

Transparency in the sample size reveals the power of the trial to readers and gives them a measure by which to assess whether the trial attained its planned size. Any differences in the planned sample size described in the trial registration (item 2), study protocol (item 3), or statistical analysis plan should be explained.

Interim analyses are used in some trials to help decide whether to stop early or to continue recruiting sometimes beyond the planned trial end (item 16b). If the actual sample size differed from the originally intended sample size for some other reason (eg, because of poor recruitment or revision of the target sample size), an explanation should be given alongside details of the revised sample size. Many reviews have found that few authors report how they determined the sample size.^222^ ^246^ ^247^ ^248^ ^249^ ^250^ ^251^ ^252^

There is no value in conducting and reporting a post hoc calculation of statistical power using the results of a trial, for example, as a pretext to explain non-significant findings; this may even mislead and confuse readers.^253^ ^254^

### Item 16b: Explanation of any interim analyses and stopping guidelines

#### Examples

“Interim analyses of effectiveness and safety endpoints were performed on behalf of the data monitoring committee on an approximately annual basis during the period of recruitment. These analyses were done with the use of the Haybittle–Peto principle and hence no adjustment was made in the final p values to determine significance.”^255^

“One interim analysis of the primary endpoint and safety data was planned for when approximately 50% of the participants had completed D28 [day 28]. Statistical significance and futility boundaries were estimated for the interim and final analysis based on 50,000 simulations from the PASS® software (NCSS, Kaysville, Utah) by simulating a group sequential test for two means assuming normality testing. At the interim analysis, the two-sided significance boundary for clinical efficacy was 0.00312 and for futility of detecting μAUT00063 > μplacebo, the one-sided O’Brien-Fleming boundary was 0.39,141. Hence, at the final analysis, the two-sided significance boundary for clinical efficacy would be 0.04761. The Independent Data Monitoring Committee (IDMC) was advised to consider making recommendations for early termination only where there was a clear demonstration of futility.”^256^

“Three planned analyses (two interim analyses and one final analysis) were performed when the observed number of events were 25, 47, and 84, respectively. Data were released by DSMC [data and safety monitoring committee] after final analysis. Efficacy stopping boundaries were based on the O’Brien-Fleming spending function. Futility boundaries were based on testing the alternative hypothesis at the 0.039 level.”^257^

“Two interim analyses to be performed using the Haybittle-Peto approach were scheduled, after enrolment of 1000 and 2000 patients, respectively. The significance level associated with both interim analyses was 0.001 and the significance level associated with the final analysis was 0.049. With this method, the overall risk of type 1 error was 5%.”^258^

#### Explanation

Numerous randomised trials enrol participants over extended periods of time. If an intervention demonstrates exceptional efficacy, the study might require early termination on ethical grounds. To mitigate this concern, assessing results as data accumulates is advisable, ideally through an independent data monitoring committee (DMC), sometimes referred to as a data and safety monitoring board (DSMB).^259^ However, conducting multiple statistical evaluations on accruing data without proper adjustment may result in misleading conclusions. For instance, examining data from a trial at five interim analyses using a P value of 0.05 would elevate the overall false-positive rate closer to 19% rather than the expected 5%. Further to stopping early for efficacy, interim analyses can be used to evaluate (1) futility, to assess whether a trial is likely to meet its objectives; or (2) safety, to assess whether there is evidence for increased risk of harms (in the intervention group relative to the comparator group).^259^ Interim analyses can also be used to reassess the sample size, using updated information from interim trial data (eg, through an internal pilot), to ensure adequate power of the trial.

Various group sequential statistical approaches exist to adjust for multiple looks (ie, analyses) at the data, and these should be predetermined in the trial protocol (see item 27a of the SPIRIT 2025 statement^78^). Using these methods, data are compared at each interim analysis, where a P value below the specified critical value by the chosen group sequential method signifies statistical significance. Some researchers view group sequential methods as a tool for decision making, while others regard them as a definitive stopping point, intending to halt the trial if the observed P value falls below the critical threshold.

Authors should disclose whether they or the DMC/DSMB performed multiple looks at the data (interim analyses). If such multiple looks occurred, it is important to specify the frequency; the triggers prompting them; the statistical methods applied (including any formal stopping rules); and whether these procedures were planned and documented in the trial protocol before the trial commenced, before the DMC examined any interim data, or at a later stage. Authors should also report the time point at which any interim analyses where conducted (and by whom); and state who decided to continue, stop or modify the trial, and whether they were blinded to the treatment allocation. Unfortunately, the reporting of interim analyses and stopping rules is frequently inadequate in published trial reports,^260^ even in cases where trials indeed halted earlier than originally planned.

### Item 17a: Who generated the random allocation sequence and the method used

#### Examples

“Randomization was done using computer-generated random numbers (Stat Trek software) by trained staff at the Soltan Mirahmad Clinic (Kashan, Iran).”^261^

“The randomization was conducted by two independent researchers who were not involved in the study using a computer random sequence generator.”^262^

#### Explanation

Randomisation eliminates selection bias at trial entry and is the crucial component of high quality randomised trials (box 5).^271^ Successful randomisation hinges on two steps: generation of an unpredictable allocation sequence and concealment of this sequence from the investigators enrolling participants (item 18).^268^ ^269^

Box 5 **Treatment allocation in trials**

The method used to assign interventions to trial participants is a crucial aspect of clinical trial design. Random assignment is the preferred method; it has been successfully used regularly in trials for more than 75 years.^263^ Randomisation has three major advantages.^264^ Firstly, when properly implemented, it eliminates selection bias, balancing both known and unknown prognostic factors, in the assignment of treatments. Without randomisation, treatment comparisons may be prejudiced, whether consciously or not, by selection of participants of a particular kind to receive a particular treatment. Secondly, random assignment permits the use of probability theory to express the likelihood that any difference in outcome between intervention groups reflects mere chance.^265^ Thirdly, random allocation, in some situations, facilitates blinding the identity of treatments to the investigators, participants, and evaluators, possibly by use of a placebo, which reduces bias after assignment of treatments.^266^ Of these three advantages, reducing selection bias at trial entry is usually the most important.^267^

Successful randomisation in practice depends on two inter-related aspects: adequate generation of an unpredictable allocation sequence and concealment of that sequence until assignment occurs.^268^ ^269^ A key issue is whether the sequence is known or predictable by the people involved in allocating participants to the comparison groups.^270^ The treatment allocation system should thus be set up so that the person enrolling participants does not know in advance which treatment the next person will get, a process termed allocation concealment.^268^ ^269^ Proper allocation concealment shields knowledge of forthcoming assignments, whereas proper random sequences prevent correct anticipation of future assignments based on knowledge of past assignments.

Who generated the random allocation sequence is important mainly for two reasons. Firstly, someone, or some group, should take responsibility for this critical trial function. Secondly, providing information on the generator might help readers to evaluate whether anyone had access to the allocation sequence during implementation. Investigators should strive for complete separation, independence, between the trial staff involved with generation of the allocation sequence and those staff who implement assignments (see explanation for item 19).

Participants should be assigned to comparison groups in the trial on the basis of a chance (random) process characterised by unpredictability (box 5). Successful randomisation in practice depends on two inter-related aspects: adequate generation of an unpredictable allocation sequence and concealment of that sequence until assignment occurs. A key issue is whether the sequence is known or predictable by the people involved in allocating participants to the comparison groups. The treatment allocation system should thus be set up so that the person enrolling participants does not know in advance which treatment the next person will receive, a process termed allocation concealment (item 18). Proper allocation concealment shields knowledge of forthcoming assignments, whereas proper random sequences (item 17) prevent correct anticipation of future assignments based on knowledge of past assignments (box 5).

Authors should provide sufficient information such that the reader can assess the methods used to generate the random allocation sequence and the likelihood of bias in group assignment. Any software used for random sequence generation should also be reported. It is important that information on the process of randomisation is included in the body of the main article and not as a separate supplementary file, where it can be missed by the reader.

The term “random” has a precise technical meaning. With random allocation, each participant has a known probability of receiving each intervention before one is assigned, and the assigned intervention is determined by a chance process and cannot be predicted. However, “random” is sometimes used inappropriately in the literature to describe trials in which non-random, “deterministic” allocation methods were used, such as alternation, hospital numbers, or date of birth. When investigators use such non-random methods, they should describe them precisely and should not use the term “random” or any variation of it. Even the term “quasi-random” is unacceptable for describing such trials. Trials based on non-random methods generally yield biased results^4^ ^268^ ^272^ ^273^ ^274^ ^275^ ^276^ ^277^; bias presumably arises from the inability to adequately conceal these more predictable, non-random sequence generation systems.

Many methods of sequence generation are adequate. However, readers cannot judge adequacy from such terms as “random allocation,” “randomisation,” or “random” without further elaboration. Authors should specify the method of sequence generation, such as a random-number table or a computerised random number generator. The sequence may be generated by the process of minimisation, a non-random but generally acceptable method.

In some trials, participants are intentionally allocated in unequal numbers to each intervention: for example, to gain more experience with a new procedure or to limit costs of the trial. In such cases, authors should report the randomisation ratio (eg, 2:1, or two treatment participants per control participant; item 9).

In a representative sample of PubMed indexed trials in 2000, only 21% reported an adequate approach to random sequence generation 220; this increased to 34% for a similar cohort of PubMed indexed trials in 2006.^221^ Two more recent studies showed further small increases to about 40%,^278^ ^279^ but another reported a stubbornly similar level of 32%.^277^ When authors report an adequate approach to random sequence generation, in over 90% of cases they report using a random number generator on a computer or a random number table.^221^ ^279^

### Item 17b: Type of randomisation and details of any restriction (eg, stratification, blocking, and block size)

#### Examples

“Treatment assignment was generated using a simple randomization scheme . . . given the open-label nature of the intervention to limit the potential bias due to predictable treatment assignment.”^280^

“Randomization (1:1) was performed by an independent researcher using computer generated random table numbers, with a block size of 20 and stratified for the indication of the IUI [intrauterine insemination] (mild male factor or unexplained subfertility).”^281^

“Participants were randomized at an individual-level (1:1 ratio) and were stratified by recruitment location (VU [Vrije University] and UvA [University of Amsterdam]). Block randomization was applied with randomly varied block sizes (6–12 allocations per block).”^262^

“Randomization was stratified by treatment centre, clinical severity (<4 vs >4 on a Western Ontario and McMaster Universities Osteoarthritis Index (WOMAC) pain subscale standardized to range from 0 to 10), and by whether patients had previously received TENS [transcutaneous electrical nerve stimulation] with randomly varied block sizes of 2, 4, and 6.”^282^

“Randomization sequence was created using Stata 9.0 (StataCorp., College Station, TX) statistical software and was stratified by center with a 1:1 allocation using random block sizes of 2,4, and 6.”^283^

#### Explanation

In trials of several hundred participants or more, simple randomisation can usually be trusted to generate similar numbers in the two trial groups 284 and to generate groups that are roughly comparable in terms of known and unknown prognostic variables.^285^ For smaller trials of fewer than around 200 participants,^286^ which are common, some form of restricted randomisation procedure to help achieve balance between groups in size or characteristics may be useful (box 6). However, larger trials of greater than approximately 200 participants may also benefit from registration. For example, they may stop before reaching their target size, they may need more power at interim analyses, or they may benefit from stratification with restriction.

Box 6 **Randomisation and minimisation**

**Simple randomisation**

Pure randomisation based on a single allocation ratio is known as simple randomisation. Simple randomisation with a 1:1 allocation ratio is analogous to a coin toss, although we do not advocate coin tossing for randomisation in a randomised trial. The term “simple” is somewhat of a misnomer. While other randomisation schemes sound complex and more sophisticated, in reality, simple randomisation is elegantly sophisticated in that it is more unpredictable and could surpass the bias prevention levels of all other alternatives.

**Restricted randomisation**

Restricted randomisation specifies any randomised approach that is not simple randomisation. Blocked randomisation is the most common form. Other means of restricted randomisation include replacement, biased coin, and urn randomisation, although these are used much less frequently.^286^

**Blocked randomisation**

Blocking can be used to ensure close balance of the numbers in each group at any time during the trial. After a block of every eight participants was assigned, for example, four would be allocated to each arm of the trial.^287^ Improved balance comes at the cost of reducing the unpredictability of the sequence. Although the order of interventions varies randomly within each block, a person running the trial could deduce some of the next treatment allocations if they discovered the block size.^288^ Blinding the interventions, using larger block sizes, and randomly varying the block size can ameliorate this problem.

**Stratified randomisation**

Stratification is used to ensure a good balance of participant characteristics in each group. By chance, particularly in small trials, trial groups may not be well matched for baseline characteristics, such as age and stage of disease. This weakens the trial’s credibility.^289^ Such imbalances can be avoided without sacrificing the advantages of randomisation. Stratification ensures that the numbers of participants receiving each intervention are closely balanced within each stratum. Stratified randomisation is achieved by performing a separate randomisation procedure within each of two or more subsets of participants (eg, those defining each centre, age, or disease severity). Stratification by centre is common in multicentre trials. Stratification requires some form of restriction, such as blocking within strata. Stratification without some form of restriction is ineffective.

**Minimisation**

Minimisation improves balance between intervention groups for several selected patient factors (eg, age).^271^ ^290^ The first patient is truly randomly allocated; for each subsequent participant, the treatment allocation that minimises the imbalance on the selected factors between groups at that time is identified. That allocation may then be used, or a choice may be made at random with a heavy weighting in favour of the intervention that would minimise imbalance (eg, with a probability of 0.8). The use of a random component is generally preferable. Minimisation has the advantage of creating small groups closely similar in terms of measurable participant characteristics at all stages of the trial.

Minimisation offers the only acceptable alternative to randomisation, and some have argued that it is superior.^291^ Conversely, minimisation lacks the theoretical basis for eliminating bias on all known and unknown factors. Nevertheless, in general, trials that use minimisation are considered methodologically equivalent to randomised trials, even when a random element is not incorporated.

It is important to indicate whether no restriction was used by stating such or by stating that simple randomisation was done. Otherwise, the methods used to restrict the randomisation, along with the method used for random selection, should be specified. For blocked randomisation, authors should provide details on how the blocks were generated (eg, by using a permuted block design with a computer random number generator), the block size or sizes, and whether the block size was fixed or randomly varied. If the trialists became aware of the block size(s), that information should also be reported as such knowledge could lead to them correctly deciphering future treatment assignments. Authors should specify whether stratification was used and, if so, which factors (eg, recruitment site, sex, disease stage) were involved; the categorisation cut-off thresholds within stratums; and the method used for restriction. Although stratification is a useful technique, especially for smaller trials, it can be complicated to implement and may not perform as well as expected if many stratifying factors are used. If minimisation (box 6) was used, it should be explicitly identified, as should the variables incorporated into the scheme; whether a random element was used should also be stated.

With blocking, although the order of interventions varies randomly within each block, individuals running the trial could deduce some of the future treatment allocations if they discovered the block size.^288^ Discovering block sizes is much more likely in unblinded trials, where treatment allocations become known after assignment (box 6). Certain techniques, such as large block sizes and randomly varying block sizes, can help prevent the deciphering of future treatment allocations. Unfortunately, particularly with unblinded trials, a review “found that very few trials used techniques that would eliminate the risk of selection bias,” and that “These findings indicate that a substantial proportion of unblinded trials are at risk of selection bias.”^292^ Indeed, in a recent study of 179 open, unblinded randomised trials, small block sizes were associated with subversion.^293^

Only 9% of 206 reports of trials in specialty journals^269^ and 39% of 80 trials in general medical journals reported use of stratification.^294^ In each case, only about half of the reports mentioned the use of restricted randomisation. Those studies and that of Adetugbo and Williams^295^ found that the sizes of the treatment groups in many trials were very often the same or quite similar, yet blocking or stratification had not been mentioned. One of a few possible causes of this close balance in numbers is under-reporting of the use of restricted randomisation, although non-random manipulation of treatment assignments is also suspected. A more recent study of 298 reports of trials in general medical journals found 69% reported the use of a stratified block method.^296^

### Item 18: Mechanism used to implement the random allocation sequence (eg, central computer/telephone; sequentially numbered, opaque, sealed containers), describing any steps to conceal the sequence until interventions were assigned

#### Examples

“Participants were centrally assigned to randomised study treatment using an interactive web response system (IWRS) . . . Block randomisation schedules were computer generated by a vendor with a block size of 6 in a randomisation ratio of 2:1 and distributed to the IWRS vendor (endpointClinical) for participant randomisation.”^297^

“For allocation concealment, numbered containers were used. The interventions were sealed in sequentially numbered identical opaque containers according to the allocation sequence.”^298^

“Furthermore, we employed syringes sequentially numbered and packaged in opaque and sealed containers. Specifically, syringes containing esmolol or placebo were centrally prepared, pre-coded based on the randomization list, and sent sequentially to the operating room immediately before administration.”^299^

“Allocation was concealed using sequentially numbered, opaque, sealed envelopes (SNOSE) prior to making the incision.”^300^

“Allocation concealment was done using sequentially numbered, sealed, opaque packages.”^261^

“The allocation sequence was concealed from the researcher (JR) enrolling and assessing participants in sequentially numbered, opaque, sealed and stapled envelopes. Aluminium foil inside the envelope was used to render the envelope impermeable to intense light. To prevent subversion of the allocation sequence, the name and date of birth of the participant was written on the envelope and a video tape made of the sealed envelope with participant details visible. Carbon paper inside the envelope transferred the information onto the allocation card inside the envelope and a second researcher (CC) later viewed video tapes to ensure envelopes were still sealed when participants' names were written on them. Corresponding envelopes were opened only after the enrolled participants completed all baseline assessments and it was time to allocate the intervention.”^301^

#### Explanation

Item 17 discussed generation of an unpredictable sequence of assignments. Of considerable importance is how this sequence is applied when participants are enrolled into the trial (box 5). A generated allocation sequence should be implemented by using allocation concealment,^269^ a critical mechanism that prevents foreknowledge of treatment assignment and thus shields those who enrol participants from being influenced by this knowledge. The decision to accept or reject a participant should be made, and informed consent should be obtained from the participant, in ignorance of the next assignment in the sequence.^302^ In summary, adequate allocation concealment safeguards knowledge of forthcoming assignments, whereas proper random sequences (item 17) prevent correct anticipation of future assignments based on knowledge of past assignments.

Allocation concealment should not be confused with blinding (item 20). Allocation concealment seeks to prevent selection bias (box 5), protects the assignment sequence before and until allocation, and can always be successfully implemented.^268^ In contrast, blinding seeks to prevent ascertainment bias, protects the sequence after allocation, and cannot always be implemented.^269^ Without adequate allocation concealment, however, even random, unpredictable assignment sequences can be subverted.^268^ ^303^

Centralised or third party assignment is especially desirable. Many good allocation concealment mechanisms incorporate external involvement. Use of a pharmacy or central computer or telephone randomisation system are common techniques. Automated assignment systems are likely to become more common.^221^ When external involvement is not feasible, an excellent method of allocation concealment is the use of numbered containers. The interventions (often medicines) are sealed in sequentially numbered identical containers according to the allocation sequence.^304^ Enclosing assignments in sequentially numbered, opaque, sealed envelopes can be a good allocation concealment mechanism if it is developed and monitored diligently.^288^ ^305^ This method can be corrupted, however, particularly if it is poorly executed. Investigators should ensure that the envelopes are opaque when held to the light, and are opened sequentially and only after the participant’s name and other details are written on the appropriate sequentially numbered sealed envelope.^288^ ^305^ ^306^

A number of methodological studies provide empirical evidence to support these precautions.^4^ ^268^ ^275^ ^276^ ^277^ ^307^ ^308^ ^309^ Trials in which the allocation sequence had been inadequately or unclearly concealed yielded larger estimates of treatment effects than did trials in which authors reported adequate allocation concealment. These findings provide strong empirical evidence that inadequate allocation concealment contributes to bias in estimating treatment effects.

Despite the importance of the mechanism of allocation concealment, published reports frequently omit such details. Among older studies, the mechanism used to allocate interventions was omitted in reports of 89% of trials on rheumatoid arthritis,^310^ 48% of trials in obstetrics and gynaecology journals,^269^ and 44% of trials in general medical journals.^294^ In a more broadly representative sample of all PubMed indexed randomised trials, only 18% reported any allocation concealment mechanism and some of those reported mechanisms were inadequate.^220^ At the same time, some trials where there is no reporting of allocation concealment may have been properly concealed, as demonstrated by inspection of their protocols.^311^

Newer studies further illuminate poor reporting of allocation concealment. Unclear reporting (ie, the authors did not provide sufficient information in the paper to allow judgment to be made on the adequacy of method of allocation concealment) was found in 78%^312^ and 85%^277^ of trials. Moreover, those two studies determined that only 27%^312^ and 14%^277^ used an adequate allocation concealment mechanism, while another found a similar level of 12%.^278^ A review of trials in journals of traditional Chinese medicine found that only 7% used adequate allocation concealment.^313^

Fortunately, reporting and conduct may be improving in recent years, for example, after the CONSORT 2010 guidelines were published.^314^ Another study found that reporting on allocation concealment and sequence generation was much better in journals that endorsed the CONSORT 2010 guidelines than in non-endorsing journals.^305^ Moreover, that study found that 57% of trials in the sample had used an adequate allocation concealment mechanism.^305^ However, other empirical studies show only modest improvements, for example, an evaluation of over 176 000 trials^315^ found that allocation concealment reporting increased from 5.1% in 1966-90 to 19.3% in 2010-18. While any improvement is encouraging, more efforts to improve conduct and reporting remain necessary.

### Item 19: Whether the personnel who enrolled and those who assigned participants to the interventions had access to the random allocation sequence

#### Examples

“Sequential randomisation codes were computer generated using permuted blocks . . . An independent study statistician generated the randomisation codes . . . Good Clinical Practice (GCP) trained research nurses or First Nations health practitioners allocate the study medicine to each mother–infant pair by selecting the next sequentially labelled (prerandomised) study medication from the appropriate stratification group. The allocation ﻿sequence number is recorded by the research team on the data collection form (DCF), the database and in the participant’s medical record.”^316^

“LabCorp Drug Development (a subcontractor to the IWRS [interactive web response system] vendor) generated the live randomisation schedules. Site personnel enrolled participants in the IWRS. The IWRS assigned participants to the trial groups per live randomisation schedules. LabCorp Drug Development didn’t have any involvement in the rest of the trial . . . Site personnel were involved in participant care and performing trial procedures throughout the trial; however, they were masked to treatment assignment.”^297^

“All participants were screened by a masked trial doctor who also obtained informed consent . . . The masked trial doctor then provided all participants with guideline-recommended care and a prescription for the trial medicine kit . . . The randomisation sequence was created using randomly permuted blocks by an independent statistician (who had no involvement in the rest of the trial).”^317^

“The details of sequence generation and group allocation were unavailable to research team members bar one unblinded research assistant. The unblinded research team member that created the randomisation sequence had no contact with participants and was not involved with data collection or analysis.”^318^

“Block randomisation was by a computer generated random number list prepared by an investigator with no clinical involvement in the trial . . . After the research nurse had obtained the patient’s consent, she telephoned a contact who was independent of the recruitment process for allocation consignment.”^319^

#### Explanation

As noted in item 18, concealment of the allocated intervention at the time of enrolment is especially important. Thus, in addition to knowing the methods used, it is also important to understand how the random sequence was implemented; specifically, whether the personnel who enrolled and those who assigned participants to the interventions had access to the random allocation sequence.

In practice, the process of randomising participants into a trial has three different steps: sequence generation, allocation concealment mechanism, and implementation (table 6). Although the same individuals may carry out more than one process under each heading, investigators should strive for complete separation of the people involved with generation and allocation concealment from the people who implement assignments. Thus, if someone is involved in the sequence generation or allocation concealment steps, ideally, they should not be involved in the implementation step. When this separation is not possible, importantly the investigators should ensure that the assignment schedule is unpredictable and locked away from even the person who generated it.

**Table 6:** Steps in a typical randomisation process

Sequence generation	Allocation concealment	Implementation
Generate allocation sequence by a random procedure	Develop allocation concealment mechanism (eg, numbered, identical bottles or sequentially numbered, sealed, opaque envelopes)	Enrol participants: • assess eligibility • discuss the trial • obtain informed consent • enrol participant in trial
	Prepare the allocation concealment mechanism using the allocation sequence from the sequence generation step	Ascertain intervention assignment (eg, opening next envelope) and assign participant to appropriate intervention
		Administer intervention

Even with flawless sequence generation and allocation concealment, failure to separate creation and concealment of the allocation sequence from assignment to trial group may introduce bias. For example, the person who was enrolling and assigning participants and who also generated an allocation sequence would likely have access to the sequence list and could consult it when interviewing potential participants for a trial. Thus, that person could bias the enrolment or assignment process, regardless of the unpredictability of the assignment sequence and the impenetrability of the allocation concealment mechanism. Investigators must therefore ensure that the assignment schedule is unpredictable and locked away (eg, in a safe deposit box in a building inaccessible to the enrolment location) from even the person who generated it. In that instance, the report of the trial should specify where the investigators stored the allocation list.

Thus, for full assessment of randomisation in a trial report, authors should confirm that personnel who enrolled and those who assigned participants to the interventions did not have access to the random allocation sequence. At minimum, authors should confirm complete separation of the people involved with generation and allocation concealment from the people involved in the implementation of assignments. If complete separation did not occur, then authors should describe how the people involved in the implementation were prevented from accessing the sequence (eg, specifying that the allocation sequence was locked in a secure location).

Sometimes those who enrol and those who assign are different people, but frequently the same individuals do both. These individuals may be termed differently by authors (eg, recruiters), but the functions remain the same.

Full assessment of implementation is not always possible from trial reports. In a sample of 199 medical journals, 63% of trial reports did not provide sufficient information to assess whether the person who generated the allocation sequence was not also the person who allocated participants to treatment groups.^306^ Only 31% of trial reports provided sufficient details ﻿on who recruited participants and who generated the allocation sequence.^306^

### Item 20a: Who was blinded after assignment to interventions (eg, participants, care providers, outcome assessors, data analysts)

#### Examples

“Whereas patients and physicians allocated to the intervention group were aware of the allocated arm, outcome assessors and data analysts were kept blinded to the allocation.”^320^

“Blinding and equipoise were strictly maintained by emphasizing to intervention staff and participants that each diet adheres to healthy principles, and each is advocated by certain experts to be superior for long-term weight-loss. Except for the interventionists (dieticians and behavioural psychologists), investigators and staff were kept blind to diet assignment of the participants. The trial adhered to established procedures to maintain separation between staff that take outcome measurements and staff that deliver the intervention. Staff members who obtained outcome measurements were not informed of the diet group assignment. Intervention staff, dieticians and behavioural psychologists who delivered the intervention did not take outcome measurements. All investigators, staff, and participants were kept masked to outcome measurements and trial results.”^321^

“This was a double-blind study with limited access to the randomisation code . . . The treatment each patient received was not disclosed to the investigator, study site staff, patient, sponsor personnel involved with the conduct of the study (with the exception of the clinical supply staff and designated safety staff), or study vendors.”^297^

“Physicians, patients, nurses responsible for referring the patients, the statistician, also the investigators who rated the patients and administered the drugs, were all blinded to the allocation.”^261^

#### Explanation

The term “blinding” (masking) refers to withholding information about the assigned interventions from people involved in the trial who may potentially be influenced by this knowledge. Blinding is an important safeguard against bias, particularly when assessing subjective outcomes.^308^

Benjamin Franklin has been credited as being the first to use blinding in a scientific experiment.^322^ He blindfolded participants so they would not know when he was applying mesmerism (a popular healing technique of the 18th century) and in so doing demonstrated that mesmerism was a sham. Since then, the scientific community has widely recognised the power of blinding to reduce bias, and it has remained a commonly used strategy in scientific experiments.

Box 7 on blinding terminology defines the groups of individuals (ie, participants, healthcare providers, data collectors, outcome assessors, and data analysts) that can potentially introduce bias into a trial through knowledge of the treatment assignments. Participants may respond differently if they are aware of their treatment assignment (eg, respond more favourably when they receive the new treatment).^308^ Lack of blinding may also influence adherence with the intervention, use of co-interventions, and risk of dropping out of the trial.

Box 7 **Blinding terminology**

For a technical term to be useful, its use and interpretation must be consistent. Authors of trials commonly use the term “double blind,” and less commonly the terms “single blind” or “triple blind.” A problem with this lexicon is that there is great variability in clinician interpretations and epidemiological textbook definitions of these terms.^323^ Moreover, a study of 200 randomised trials reported as double blind demonstrated 18 different combinations of groups actually blinded when the authors of these trials were surveyed, and approximately one in every five of these trials—reported as double blind—did not blind participants, healthcare providers, or data collectors.^324^

This research demonstrates that terms are ambiguous and, as such, authors and editors should abandon their usage in isolation without defining them. Authors should instead explicitly report the blinding status of the people involved for whom blinding may influence the validity of a trial.

The healthcare providers include all personnel (eg, physicians, chiropractors, physiotherapists, nurses) who care for the participants during the trial. Data collectors are the individuals who collect data on the trial outcomes. Outcome assessors are the individuals who determine whether a participant did experience the outcomes of interest.

Some researchers have also advocated blinding and reporting the blinding status of the data monitoring committee and the manuscript writers.^325^ Blinding of these groups is uncommon and the value of blinding them is debated.^326^

Sometimes one group of individuals (eg, the healthcare providers) is also the same individuals fulfilling another role in a trial (eg, the data collectors). Even if this is the case, the authors should state the blinding status of these groups to allow readers to judge the validity of the trial.

Unblinded healthcare providers may introduce similar biases; and unblinded data collectors may differentially assess outcomes (eg, frequency or timing), repeat measurements of abnormal findings, or provide encouragement during performance testing. Unblinded outcome assessors may differentially assess subjective outcomes, and unblinded data analysts may introduce bias through the choice of analytical strategies, such as the selection of favourable time points or outcomes and by decisions to remove patients from the analyses. These biases have been well documented.^32^ ^308^ ^325^ ^327^ ^328^ ^329^

Blinding, unlike allocation concealment (item 18), may not always be appropriate or possible. In pragmatic trials (trials that try to make the experience as close as real life so as to understand real world effectiveness), blinding of participants and healthcare providers would decrease the pragmatism of the trials, since patients in real life are not blinded.^330^ An example where blinding is impossible is a trial comparing levels of pain associated with sampling blood from the ear or thumb.^331^ However, in randomised trials for which blinding is possible, lack of blinding has usually been associated with empirical evidence of exaggeration in treatment effect estimates.^276^ ^308^ ^332^ ^333^ ^334^ ^335^ ^336^ Blinding is particularly important when outcome measures involve some subjectivity, such as assessment of pain. Yet, blinding may not be as important in certain fields or with certain outcomes. For example, blinding of data collectors and outcome assessors is unlikely to matter for objective outcomes, such as death from any cause. Indeed, some methodological investigations have not found that lack of blinding is associated with empirical evidence of bias in treatment effect estimates.^337^ ^338^ ^339^ ^340^ ^341^ ^342^ ^343^ Even then, however, lack of participant or healthcare provider blinding can lead to other problems, such as differential attrition.^344^ In certain trials, especially surgical trials, blinding of participants and healthcare providers is often difficult or impossible, but blinding of data collectors and outcome assessors for both benefits and harms is often achievable and recommended. For example, lesions can be photographed before and after treatment and assessed by an external observer.^345^ Regardless of whether blinding is possible, authors can and should always state who was blinded (ie, participants, healthcare providers, data collectors, data analysts, and/or outcome assessors).^346^ ^347^

However, authors frequently do not report whether blinding was used.^348^ ^349^ For example, reports of 51% of 506 trials in cystic fibrosis,^350^ 33% of 196 trials in rheumatoid arthritis,^310^ and 38% of 68 trials in dermatology^295^ did not state whether blinding was used. Similarly, a more recent review found that the reports of 38% of 622 trials in ﻿high impact anaesthesiology journals did not explicitly describe the trial as blinded or non-blinded.^351^ Moreover, when describing some form of blinding, the most used term was the ambiguous “double blind.”^351^ Authors should explicitly state who was blinded, but only 14% of 622 trials explicitly reported whether the three key groups of individuals—that is, the participants, healthcare providers, and data collectors—were blinded or not.^351^ The rate did improve from 10% to 26% over the years of that review, but more improvement is needed. Until authors of trials improve their reporting of blinding, readers will have difficulty in judging its adequacy.

The term “masking” is sometimes used in preference to “blinding” to avoid confusion with the medical condition of being without sight. However, “blinding” in its methodological sense appears to be more universally understood worldwide and to be generally preferred for reporting clinical trials.^344^ ^345^ ^352^

### Item 20b: If blinded, how blinding was achieved and description of the similarity of interventions

#### Examples

“Jamieson Laboratories Inc. provided 500-mg immediate release niacin in a white, oblong, bisect caplet. We independently confirmed caplet content using high performance liquid chromatography . . . The placebo was matched to the study drug for taste, color, and size, and contained microcrystalline cellulose, silicon dioxide, dicalcium phosphate, magnesium stearate, and stearic acid.”^353^

“Placebo tablets were identical to NAC [N‐acetylcysteine] tablets in color, shape, size, and odor. They were all kept in identical containers and were administered by an investigational drug pharmacist.”^261^

“The study treatment and placebo tablets and bottles were identical in physical appearance . . . The IWRS [interactive web response system] housed treatment codes and bottle numbers for study treatment. In case of an emergency, the investigator had the sole responsibility for determining if unmasking of a participant’s treatment assignment was warranted to provide appropriate medical care. Participant safety was always the first consideration in making such a determination. The IWRS was programmed with blind-breaking instructions to guide the investigator on how to obtain treatment assignment in the event of an emergency unmasking. The investigator was requested to contact the medical monitor promptly in case of any treatment unmasking. If a participant’s treatment assignment was unmasked, the sponsor was to be notified within 24 h after unmasking. The date and reason for the unmasking were recorded in the source documentation and electronic case report form, as applicable. Investigators broke the masking for four participants: one in ELEVATE UC 12 (on etrasimod) and three in ELEVATE UC 52 (on etrasimod).”^297^

#### Explanation

Blinding of participants, healthcare providers, data collectors, and outcome assessors in a trial requires adequate procedures to both achieve and maintain blinding.^346^ ^347^ Just as we seek evidence of adequate allocation concealment to assure us that assignment was truly random, we seek evidence on the method of blinding.

If researchers contend that the trial investigators, participants, and assessors were blinded, then they should provide information about the mechanism used to establish blinding (eg, placebo identical to the experimental intervention, sham intervention, sham surgery).^346^ ^347^ They should describe the similarity of treatment characteristics (eg, route of administration, appearance, smell, taste) and where relevant methods used to mask some characteristics of the treatments (eg, use of special flavours to mask a distinctive taste, opaque coverage to conceal intravenous treatments with different appearances, double-dummy procedures).^346^ ^347^

Blinding can be difficult to maintain over time because of dosage adaptation over time or the occurrence of specific side effects. Specific procedures to maintain blinding can be implemented (eg, centralised assessment of side effects, centralised adapted dosage, or provision of sham results of complementary investigations).

Even if blinding of participants and healthcare providers is not possible, blinding data collectors and outcome assessors could still be implemented to limit ascertainment bias. This could be achieved, for example, through centralised assessment of complementary investigation (eg, anonymised radiography), physician mediated data (eg, video, photography, audiotape), and clinical events (eg, adjudication of clinical events from extract of the case report form).

Details of how blinding was achieved are important because slight, but discernible, differences between interventions can lead to large problems in bias. Notably, inadequate matching related to discernible differences in colour and taste seem particularly problematic.^354^ It is important that authors report any known compromises in blinding. For example, authors should report if it was necessary to unblind any participants at any point during the conduct of the trial. Moreover, authors should report the risk of unblinding but, unfortunately, such reporting is rare. In a random sample of 300 publications describing blinded randomised clinical trials indexed in PubMed, only 8% reported on risk of unblinding.^355^ It is also important to report any procedures, pretrial or concurrent, that are intended to reduce or evaluate risk of compromised blinding.^354^ ^355^ ^356^ Indeed, some pretrial assessments of unblinding may be helpful in reducing the risk of unblinding in the eventual randomised trial. Thus, authors of randomised trial articles should report procedures to avoid, document, and address cases of overt unblinding.^354^ ^355^

Where appropriate, authors should also describe any procedures used for emergency unblinding (ie, disclosing the assigned intervention of a trial participant for specific reasons such as harms). They should indicate whether they used fixed code to indicate group assignment (eg, A=group 1; B=group 2) or a unique code for each participant. Use of a fixed code will increase the risk of unblinding because unblinding a participant could result in unblinding several or all trial participants.^6^ ^286^ ^357^

Some people have advocated testing for blinding by asking participants or healthcare providers at the end of a trial whether they think the participant received the experimental or control intervention.^358^ Because participants and healthcare providers will frequently know whether the participant has experienced the primary outcome, this makes it difficult to determine whether their responses reflect failure of blinding or accurate assumptions about the efficacy of the intervention.^359^ Thus, given the uncertainty this type of information provides, the usefulness of tests of blinding has been questioned.^354^ ^355^ ^360^ Testing for blinding was not an included item in CONSORT 2010, and still is not in CONSORT 2025. Nevertheless, if investigators decide to conduct tests of blinding, we encourage them to completely report their findings with appropriate limitations.^356^

### Item 21a: Statistical methods used to compare groups for primary and secondary outcomes, including harms

#### Examples

“The primary outcome was analysed using a mixed effects log-binomial model to generate an adjusted risk ratio (RR) and an adjusted risk difference (using an identity link function), including centre as a random effect. Statistical significance of the treatment group parameter was determined (p value generated) through examination of the associated χ2 statistic (obtained from the log-binomial model which produced the RR). Binary secondary outcomes were analysed as per the primary outcome. Time to hCG [human chorionic gonadotrophin] resolution was considered in a competing risk framework to account for participants who had surgical intervention for their ectopic pregnancy. A cumulative incidence function was used to estimate the probability of occurrence (hCG resolution) over time. A Fine and Gray model was then used to estimate a subdistribution adjusted hazard ratio (HR) directly from the cumulative incidence function. In addition, a further Cox proportional hazard model was fitted and applied to the cause-specific (non-surgical resolution) hazard function and used to generate an adjusted HR. Return to menses was analysed using a Cox regression model. Number of hospital visits associated with treatment was analysed using a Poisson regression model, including centre as a random effect to generate an adjusted incidence ratio.”^255^

“For the primary continuous outcome and secondary outcomes, linear mixed-effect models were used, with outcome measurement (at the two follow-up timepoints) as the dependent variable. The models included fixed effects for timepoint, treatment, timepoint by treatment interactions, the baseline measure of the outcome, and therapist, assuming a linear relationship between baseline and outcome. The dichotomous outcome of recovery in the delusion was analysed using a logistic mixed-effect model. Persecutory delusion conviction was analysed as a continuous and also as a dichotomous (recovery) variable. The models included a random intercept for participant, an unstructured correlation matrix for the residuals, and were fitted using restricted maximum likelihood estimation . . . For each outcome and timepoint, we report the treatment effect estimate as the adjusted mean difference between groups, its SE [standard error], 95% CIs [confidence intervals], and p value. In addition, we report estimates for Cohen’s d effect sizes as the adjusted mean difference of the outcome (between the groups) divided by the sample SD [standard deviation] of the outcome at baseline.”^361^

“Analyses followed a prespecified statistical analysis plan. The primary outcome (ODQ [Oswestry Disability Questionnaire] score at 18 weeks after randomisation) was compared between groups with a linear regression model, adjusted for baseline ODQ, with centre as a random effect. ODQ score, visual analogue scores (VAS) for back pain, VAS for leg pain, MRM [modified Roland-Morris] outcome score, and COMI [Core Outcome Measures Index] score at all follow-up visits were analysed with a repeated measures mixed-effects model adjusting for baseline outcome measure, treatment group, time (as a continuous variable), and a time-treatment arm interaction (if significant). Centre and participant were random effects in the repeated measures models. A second model adjusted for other prespecified variables, age, sex, duration of symptoms, body-mass index, and size of disc prolapse (as a percentage of the diameter of the spinal canal, categorised as <25%, 25–50%, or >50%).”^362^

“We analysed the primary outcome (between-group difference in the SPPB [short physical performance battery] at 12 months) using linear mixed models, adjusted for baseline measurements, minimisation variables (age, sex and CKD [chronic kidney disease] category) and a random effect variable for recruitment site. We analysed secondary outcomes using repeated measures mixed models, including all participants and including data from all available timepoints. Models were adjusted for baseline values and the minimisation variables. We conducted time-to-event analyses (time to death, time to commencing renal replacement therapy) using Cox proportional hazards models adjusted for minimisation variables. All participants were included in these analyses, with participants censored at the point of dropout or truncation of follow-up for those not reaching the analysis endpoint before 24 months. For all analyses, we took a two-sided p value of < 0.05 as significant with no adjustment for multiple testing.”^363^

#### Explanation

Various methods can be used to analyse data, and it is crucial to ensure that the chosen approach is suitable for the specific context. Specifying the statistical procedures and software used for each analysis is essential, and additional clarification may be required in the results section of the report. Authors should describe the statistical methods insufficient detail to allow a knowledgeable reader with access to the original data to verify the reported results, as emphasised by the ICMJE (https://www.icmje.org/). It is also important to elaborate on specific aspects of the statistical analysis, such as the intention-to-treat approach.

Details of all statistical analyses are frequently prespecified in a statistical analysis plan, a document that accompanies the trial protocol. In the report of the trial results, authors should detail and justify any deviation from the statistical analysis plan or from the protocol if no statistical analysis plan was developed. They should clarify which analyses were prespecified and which were post hoc.

Most analysis approaches provide an estimate of the treatment effect, representing the difference in outcomes between comparison groups, and authors should also indicate the effect measure (eg, absolute risk) considered. Authors should accompany this with a CI for the estimated effect, delineating a central range of uncertainty regarding the actual treatment effect. The CI may be interpreted as the range of values for the treatment effect that is compatible with the observed data. Typically, a 95% CI is presented, signifying the range anticipated to encompass the true value in 95 of 100 similar studies.

Study findings can also be assessed in terms of their statistical significance. The P value represents the probability that the observed data (or a more extreme result) could have arisen by chance when the interventions did not truly differ. The statistical significance level that will be used should be reported. In the results section, actual P values (for example, P=0.001) are strongly preferable to imprecise threshold reports such as P<0.05.^364^ ^365^

Some trials may use bayesian methods.^366^ ^367^ ^368^ ^369^ In this case, the choices of priors, computational decisions, and any modelling methods used should be described. Most bayesian trials so far have been for early phases of drug development, but this approach can be applicable to any phase. Typically, results are presented as treatment effects along with credible intervals.

Where an analysis lacks statistical power (eg, harms outcomes), authors may prefer descriptive approaches over formal statistical analysis.

While the necessity for covariate adjustments is generally reduced in randomised trials compared with epidemiological studies, considering an adjusted analysis can have value in terms of increased power and precision, particularly if there is an indication that one or more variables may have prognostic value.^370^ It is preferable for adjusted analyses to be explicitly outlined in the study protocol (item 3). For instance, it is often advisable to make adjustments for stratification variables,^371^ in keeping with the principle that the analysis strategy should align with the study design. In the context of randomised trials, the decision to make adjustments should not be based on whether there are baseline covariates that are statistically significantly different between randomised groups. The testing of baseline imbalance in covariates should be avoided,^370^ as if randomisation is properly conducted, then by definition, any differences in baseline covariates between treatment arms are random. The rationale for any adjusted analyses and the statistical methods used should be specified, along with clarifying the choice of covariates that were adjusted for, indicating how continuous variables were handled (eg, linear, modelled with splines),^372^ and specifying whether the analysis was planned or post hoc. Reviews of published studies show that reporting of adjusted analyses is inadequate with regard to all of these aspects.^373^ ^374^

Multiplicity issues are prevalent in trials and merit special consideration, especially in cases involving multiple primary outcomes, multiple time points stemming from repeated assessments of an outcome, multiple planned analyses for an outcome (such as interim or subgroup analyses (item 21d)), or analyses of numerous secondary outcomes (see CONSORT outcomes extension for more details).^21^ Any methods used to mitigate or account for multiplicity should be described. If no methods have been used to account for multiplicity (eg, not applicable, or not considered), then this should also be reported, particularly when a large number of analyses has been carried out.

### Item 21b: Definition of who is included in each analysis (eg, all randomised participants), and in which group

#### Examples

“The primary statistical analyses were performed according to the treatment to which the participants were randomly assigned. The analyses of the efficacy and safety outcomes (other than adverse events) included all available data from all randomized participants who contributed at least 1 value after baseline for the outcome of interest. The data that were obtained after a participant enrolled in another trial of an investigational treatment were excluded from the analyses. However, the participant was included in the analyses if that participant contributed at least 1 value after baseline for the outcome of interest prior to enrolling in another trial.”^375^

“Efficacy outcomes were assessed using intention-to-treat analysis (ie, the full set of all randomly assigned patients). Safety outcomes were assessed using the safety analysis set of all randomly allocated patients exposed to at least one dose of randomised intervention.”^376^

“Efficacy analyses and other exploratory analyses were performed in the full analysis set (defined as all patients randomly assigned to the study, including those who did not receive a dose of study treatment). Safety analyses were performed in the safety analysis set (defined as patients who received at least one dose of study treatment). The per protocol set was defined as all patients in the full analysis set who complied with the protocol in terms of exposure to study treatment, availability of tumour assessments, and absence of major protocol deviations likely to affect efficacy outcomes. Sensitivity analyses of the primary endpoint were performed on the per protocol analysis set.”^377^

“The primary analysis population was defined as all participants who completed baseline and 36-week assessments. The primary analysis of the primary outcome, AMCA [amended motor club assessment] score at 36 weeks, followed a modified intention-to-treat approach, regardless of compliance to the intervention, but did exclude patients who were deemed ineligible after randomisation, those who withdrew from the trial and were unwilling for their previously collected data to be used, or those who did not provide baseline and week 36 measurements.”^378^

#### Explanation

A key strength of a randomised trial design is the avoidance of bias when randomly allocating trial participants to interventions. To preserve the benefits of randomisation, all randomised participants are included in the analysis and retained in the group to which they were allocated. Meeting these two conditions defines an intention-to-treat analysis—which is widely recommended as the preferred analysis strategy.^379^ ^380^ ^381^ However, strict adherence to an intention-to-treat analysis is often difficult to achieve owing to missing outcomes for some trial participants (item 21c) or non-adherence to the trial intervention protocol. While imputation of missing outcomes would allow an intention-to-treat analysis, it does not guarantee an avoidance of bias except under strong assumptions about the missing data which may be unknown.

Various strategies for performing intention-to-treat analyses in the presence of missing outcome data are available.^382^ When the number of missing outcomes is not large, the analysis population could be all randomised participants with outcome observed (known as an “available case” population) under a plausible missing data mechanism, and sensitivity analyses could be performed exploring departures from this assumption (thereby using all randomised participants at least in sensitivity analyses).^382^ Concerns may arise when the frequency or the causes of dropping out differ between the intervention groups. Striving for intention-to-treat analysis by imputing values for participants with missing outcomes may lead to use of inadequate methods such as last observation carried forward.^382^ ^383^ ^384^ ^385^

Regardless of whether all randomised participants (completely observed outcomes or imputed outcomes) or a subset of randomised participants with observed outcomes are included in the primary analysis, the analysis population should be described. Authors often describe performing analyses on a “modified intention-to-treat” population to cover departures from a strict intention-to-treat that excludes participants who did not adequately adhere to the protocol such that they did not receive some minimum amount of the intervention—in such cases, what defines the minimum amount of the intervention should be explained (eg, those participants receiving at least one dose of the medication). It is also common to include analyses based on a per protocol population, which includes participants completing the study with no major protocol deviations. Excluding participants may compromise the randomisation and lead to biased estimates of treatment effects if appropriate methods are not used. Other analysis populations are possible (eg, a safety population), and their rationale and definition should be explained. Thus, authors should clearly define which participants are included in each analysis and in which intervention group and avoid terms such as “modified intention-to-treat” or “per protocol” analysis.

### Item 21c: How missing data were handled in the analysis

#### Examples

“Regarding the multiple imputation procedure, briefly, for each outcome, the analysis model used was a linear regression with treatment arm, baseline outcome, and ethnicity (randomization stratifier) as explanatory variables. The imputation models contained all the variables of the analysis model(s) as well as factors associated with missingness: age (identified empirically to predict missingness, P = .03) and adherence (number of doses taken of either vitamin D or placebo, P < .001).”^386^

“To consider the potential impact of missing data on trial conclusions, we used multiple imputation (data missing at random) and sensitivity analysis (data not missing at random). Multiple imputation by chained equations was performed using the “mi impute chained” command in Stata. We used a linear regression model to impute missing outcomes for the HOS ADL [activities of daily living subscale of the hip outcome score] at eight months post-randomisation. Variables in the imputation model included all covariates in the analysis model (baseline HOS ADL (continuous), age (continuous), and sex). In addition, we included other variables that were thought to be predictive of the outcome (lateral centre-edge angle, maximum α angle, Kellgren-Lawrence grade, and baseline HADS score). Imputations were run separately by treatment arm and based on a predictive mean matching approach, choosing at random one of the five HOS ADL values with the closest predicted scores. Missing data in the covariates that were included in the multiple imputation model were imputed simultaneously (multiple imputation by chained equation approach). Sensitivity analysis was performed using the “rctmiss” command in Stata, and we considered scenarios where participants with missing data in each arm were assumed to have outcomes that were up to 9 points worse than when data were missing at random.”^242^

“Analyses for the 2 primary outcomes compared each treatment with usual care using multiple imputation to handle missing data and a Bonferroni-corrected 2-tailed type I error of .025. We performed 20 imputations with a fully conditional specification using Proc MI in SAS. Imputation was performed with the following prespecified variables: age, study group, study site, clinic, sex, race and ethnicity, body mass index, exercise frequency at baseline, education, employment status, smoking status, other medical conditions at baseline, number of medications used for spine pain at baseline, duration of pain at baseline, number of previous pain episodes, STarT Back score, baseline ODI, baseline self-efficacy, baseline EQ-5D-5L, and scores for patient-reported outcomes at every follow-up point (ODI [Oswestry Disability Index], cost, Lorig et al self-efficacy scale, and EQ-5D-5L [EuroQol 5-dimensional 5-level questionnaire]). Each imputed data set was analyzed separately using Proc GENMOD in SAS (with an identity link and normally distributed errors for ODI and a log link and Poisson-distributed errors for spine-related spending).”^387^

“Missing peak V̇o2 [oxygen consumption] data at week 20, regardless of the type of intercurrent event, was imputed using multiple imputation methodology under the missing at random assumption for the primary analysis. Sensitivity analyses were performed by exploring a missing not at random assumption in the imputation of peak V̇o2. The imputation model used a regression multiple imputation, which includes treatment group, baseline respiratory exchange ratio, persistent atrial fibrillation (yes or no), age, sex, baseline peak V̇o2, baseline hemoglobin level, baseline estimated glomerular filtration rate, baseline body weight, baseline KCCQ [Kansas City cardiomyopathy questionnaire] total symptom score, baseline NYHA [New York Heart Association] class, and baseline average daily activity units (refers to 10 hours of wearing during the awake time for ≥7 days unless otherwise specified). Treatment group, persistent atrial fibrillation (yes or no), baseline NYHA class, and sex were treated as categorical variables. Fifty imputed data sets were generated. Each of the imputed data sets was analyzed using the analysis of covariance model of the primary analysis. Least square mean (LSM) treatment difference and the standard error were combined using Rubin’s rules to produce an LSM estimate of the treatment difference, its 95% CI [confidence interval], and P value for the test of null hypothesis of no treatment effect.”^388^

“Multiple imputation was preplanned for the primary outcome measure in the case of missing data; however, because there were no missing data relating to ventilator-free days, imputation was not required.”^389^

#### Explanation

Missing data are common when conducting medical research. Collecting data on all study participants can be challenging even in a trial that has mechanisms to maximise data capture. Missing values can occur in either the outcome or in one or more covariates, or usually both. There are many reasons why missing values occur in the outcome. Patients may stop participating in the trial, withdraw consent for further data collection, or fail to attend follow-up visits; all of which could be related to the treatment allocation, specific (prognostic) factors, or experiencing a specific health outcome.^390^ Missing values could also occur in baseline variables, such that all the necessary data needed to conduct the trial have been only partially recorded. Despite the ubiquity of missing data in medical research, the reporting of missing data and how they are handled in the analyses is poor.^391^ ^392^ ^393^ ^394^ ^395^ ^396^ ^397^ ^398^ ^399^

Many trialists exclude patients without an observed outcome. Once any randomised participants are excluded, the analysis is not strictly an intention-to-treat analysis. Most randomised trials have some missing observations. Trialists effectively must choose between omitting the participants without final outcome data, imputing their missing outcome data, or using model based approaches such as fitting a linear mixed model to repeated measures data.^371^ A complete case (or available case) analysis includes only those participants whose outcome is known. While a few missing outcomes will not cause a problem, many trials have more than 10% of randomised patients with missing outcomes.^391^ ^392^ ^393^ ^394^ ^395^ ^396^ ^397^ ^398^ This common situation will result in loss of power by reducing the sample size, and bias may well be introduced if being lost to follow-up is related to a participant’s response to treatment. There should be concern when the frequency or the causes of dropping out differ between the intervention groups.

Participants with missing outcomes can be included in the analysis if their outcomes are imputed (ie, their outcomes are estimated from other information that was collected) or if using a model based approach. Imputing the values of missing data allows the analysis to potentially conform to intention-to-treat analysis but requires strong assumptions, which may be hard to justify. Simple imputation methods are appealing, but their use may be inadvisable as they fail to account for uncertainty introduced by missing data and may lead to invalid inferences (eg, estimated standard errors for the treatment effect will be too small).^400^ For randomised trials with missing data within repeated measures data, model based approaches such as fitting a linear mixed model can be used to estimate the treatment effect at the final time point which is valid under a missing-at-random assumption. A model is fit at a (limited) number of time points following randomisation, by including fixed effects for time and randomised group and their interaction.^371^

Another approach that is sometimes used is known as “last observation carried forward,” in which missing final values of the outcome variable are replaced by the last known value before the participant was lost to follow-up. Although this method might appear appealing through its simplicity, the underlying assumption will rarely be valid, so the method may introduce bias, and makes no allowance for the uncertainty of imputation. The approach of last observation carried forward has been severely criticised.^383^ ^384^ ^401^ Sensitivity analyses should be reported to understand the extent to which the results of the trial depend on the missing data assumptions and subsequent analysis (item 21d).^402^ When the findings from the sensitivity analyses are consistent with the results from the primary analysis (eg, complete case for the primary analysis and multiple imputation for a sensitivity analysis), trialists can be reassured that the missing data assumptions and associated methods had little impact on the trial results.^403^

Regardless of what data are missing, how such data are to be analysed and reported needs to be carefully planned. Authors should provide a description on how missing data were handled in sufficient detail to allow for the analysis to be reproduced (in principle; box 8).

Box 8 **Guidance for reporting analytical approaches to handle missing data (adapted from Hussain et al^404^)**

**Methods**

1. Report any strategies used to reduce missing data throughout the trial process.
2. Report if and/or how the original sample size calculation accounted for missing data (item 16a) and the justification for these decisions. Report if and/or how the sample size was reassessed during the course of the trial (item 16b).
3. Report the assumption about the missing data mechanism for the primary analysis and the justification for this choice, for all outcomes. For multiple imputation methods, report^405^:
  - What variables were included in the imputation procedure?
  - How were non-normally distributed and binary/categorical variables dealt with?
  - If statistical interactions were included in the final analyses (item 21a), were they also included in imputation models?
  - Was imputation done separately or by randomised group?
  - How many imputed datasets were created?
  - How were results from different imputed datasets combined?
4. Report the method used to handle missing data for the primary analysis (eg, complete case, multiple imputation) and the justification for the methods chosen, for all outcomes. Include whether or which auxiliary variables were collected and used.
5. Report the assumptions about the missing data mechanism (eg, missing at random) and methods used to conduct the missing data sensitivity analyses for all outcomes, and the justification for the assumptions and methods chosen.
6. Report how data that were truncated due to death or other causes were handled with a justification for the method(s) (if relevant).

**Results**

1. Report the numbers and proportions of missing data in each trial arm.
2. Report the reasons for missing data in each trial arm.
3. Report a comparison of the characteristics of those with observed and missing data.
4. Report the primary analysis based on the primary assumption about the missing data mechanism, for all outcomes.
5. Report results of the missing data sensitivity analyses for all outcomes. As a minimum, a summary of the missing data sensitivity analyses should be reported in the main paper with the full results in the supplementary material.

**Discussion**

1. Discuss the impact of missing data on the interpretation of findings, considering both internal and external validity. For multiple imputation, include whether the variables included in the imputation model make the missing-at-random assumption plausible.

### Item 21d: Methods for any additional analyses (eg, subgroup and sensitivity analyses), distinguishing prespecified from post hoc

#### Examples

“We conducted prespecified sensitivity analyses to examine the effect of our assumption that participants who withdrew or were lost to follow-up returned to smoking: (1) a complete case analysis and (2) multiple imputation to impute missing smoking abstinence and reduction data. Multiple imputation was performed using the fully conditional specification approach with 5 imputed data sets and results combined using the Rubin rules (eMethods in Supplement 2). Other prespecified sensitivity analyses examined the effect of imbalances in baseline participant characteristics using multiple logistic regression models to estimate odds ratios and 95% CIs [confidence intervals] for point prevalence abstinence at 12 and 24 weeks, adjusting for characteristics for which the absolute value of the standardized difference was 0.1 or greater. We conducted additional post hoc analyses: (1) to examine potential clustering by site using generalized linear mixed models with a random effect for site to estimate odds ratios and 95% CIs for point prevalence abstinence at 12 and 24 weeks, and (2) to compare the baseline characteristics of participants with self-reported smoking data at 12 weeks (primary end point) with those of participants without self-reported smoking data. Statistical analyses were performed using SAS statistical software (version 9.4; SAS Institute).”^406^

“Several prespecified sensitivity analyses were done. First, assessment of the effect of missing data on the primary outcome was done using multiple imputation by chained equations method (MICE). This imputation model included all the variables in the primary ITT [intention to treat] analysis, secondary outcomes (from each timepoint), and baseline variables associated with the missingness of the primary outcome. 20 imputed datasets were generated and combined using Rubin’s rules, and the primary analysis model was then repeated using the imputed data. We specified a priori the following potential exploratory analyses to assess effect modification on the primary outcome: baseline hypertension, baseline MMSE [Mini-Mental State Examination], baseline age, time since Alzheimer’s disease diagnosis, baseline brain volume, and change in systolic blood pressure. A post-hoc analysis was also done to investigate for differences between aggregated and disaggregated MRI [magnetic resonance imaging] data (according to MRI scanner modality) for the primary outcomes.”^407^

“Four sensitivity analyses were done examining the primary outcome: restricted to women who had not received antibiotics in the 7 days before delivery, to examine whether any masking of a prophylactic effect was occurring by inclusion of pretreated women; excluding women prescribed antibiotics (other than the trial intervention) within the first 24 h after delivery, and who might therefore already have had an infection at the time of administration of the intervention; restricted to women whose primary outcome was obtained between weeks 6 and 10 after delivery to exclude any biases by over-reporting of outcomes from data returned at a later timepoint or under-reporting of outcomes in data returned at an earlier timepoint; and including centre as a random effect. No subgroup analyses were planned; however, we did a post-hoc subgroup analysis of the primary outcome according to mode of birth (forceps or vacuum extraction). More stringent 99% CIs [confidence intervals] are presented for the estimate of RR [risk ratio] for this post-hoc subgroup analysis.”^408^

“A prespecified subgroup analysis for the primary outcomes, testing for an interaction for baseline anxiety, depression, and opioid use, defined using their median values was completed. Prespecified sensitivity analyses for the primary outcome, excluding participants included in process evaluation interviews, adjusting for the imbalance of death, and split by baseline pain disorders were also completed. Because of the potential for type I error due to multiple comparisons, findings for analyses of secondary end points should be interpreted as exploratory. Statistical analyses were conducted using Stata version 16.1 (StataCorp).”^409^

#### Explanation

Sensitivity analyses can be important additional analyses to examine the robustness of the primary trial results under a range of assumptions about the data, methods, and models that differ from those of the primary analysis. When the findings from a sensitivity analysis are consistent with the primary trial findings, trialists can be confident that any assumptions in the primary analysis had little impact—strengthening the trial results. Morris and colleagues provide a principled approach to guide any sensitivity analyses by posing three questions to trialists: does the proposed sensitivity analysis address the same question as the primary analysis; is it possible for the proposed sensitivity analysis to return a different result to the primary analysis; and if the results do differ, is there any uncertainty as to which will be believed.^402^ ^410^

Subgroup analyses are another set of additional analyses that are widely carried out and reported.^411^ ^412^ ^413^ ^414^ Here, the focus is on those analyses that look for evidence of a difference in treatment effect in complementary subgroups (eg, older and younger participants), a comparison known as a test of interaction.^415^ Empirical analyses of subgroup difference claims for factors such as age, sex, race, ethnicity, and other factors show selective reporting, frequent lack of proper statistical support, and poor independent corroboration.^416^ ^417^ ^418^

A common but misleading approach is to compare P values for separate analyses of the treatment effect in each group. Categorising continuous variables to create subgroups is often done for simplicity and because it is perceived as easier to understand and communicate. Major limitations of the approach include the splitting of a continuous variable into discrete subgroups by arbitrarily chosen cut-off points that lack clinical or biological plausibility, which loses information, and thus reduces statistical power.^419^ Choosing cut-off points based on achieving statistical significance should be avoided. It is incorrect to infer a subgroup effect (interaction) from one significant (in one subgroup) and one non-significant P value (in another subgroup). The rationale for any subgroups should be outlined (including how they are defined), along with whether the subgroups were specified a priori in the protocol or statistical analysis plan or were done post hoc. Because of the high risk for spurious findings, subgroup analyses are often discouraged. Post hoc subgroup comparisons (analyses done after looking at the data) are especially likely not to be confirmed by further studies. Most of these analyses do not have substantial credibility.

An alternative and stronger approach, which avoids the need to specify cut-off points to assess the interaction between a continuous variable (eg, age) and treatment effect would be to fit a regression model, which can be presented graphically to examine how the estimated treatment effects varies with the level of the variable.^420^ These analyses are more complex, requiring model assumptions to capture the relationship (linear or non-linear) between the variable and the treatment effect. Authors should clearly describe the statistical methods used to explore the treatment-covariate interaction.

## CONSORT 2025: Results

### Item 22a: For each group, the numbers of participants who were randomly assigned, received intended intervention, and were analysed for the primary outcome

#### Examples

**Fig 1:**
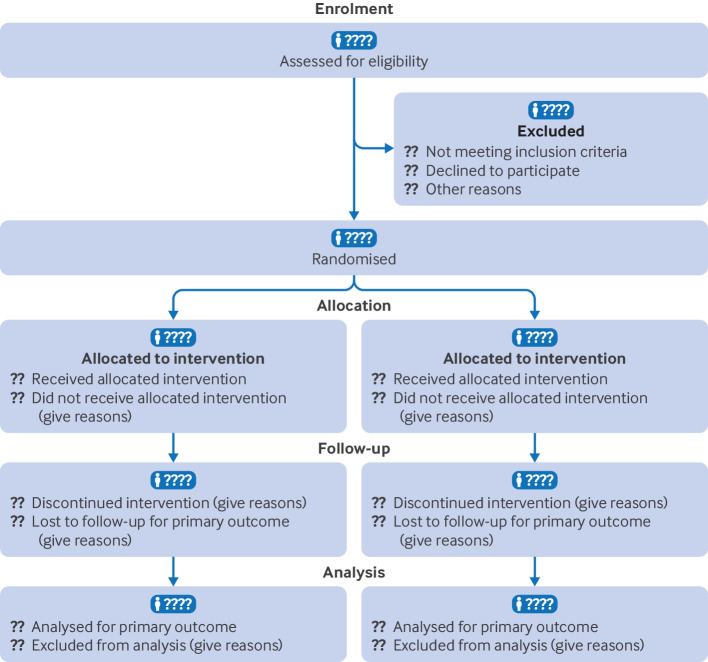
CONSORT 2025 flow diagram. Flow diagram of participant progress through the phases of a two group, parallel randomised trial (ie, enrolment, intervention allocation, follow-up, and data analysis). CONSORT=Consolidated Standards of Reporting Trials

**Fig 2:**
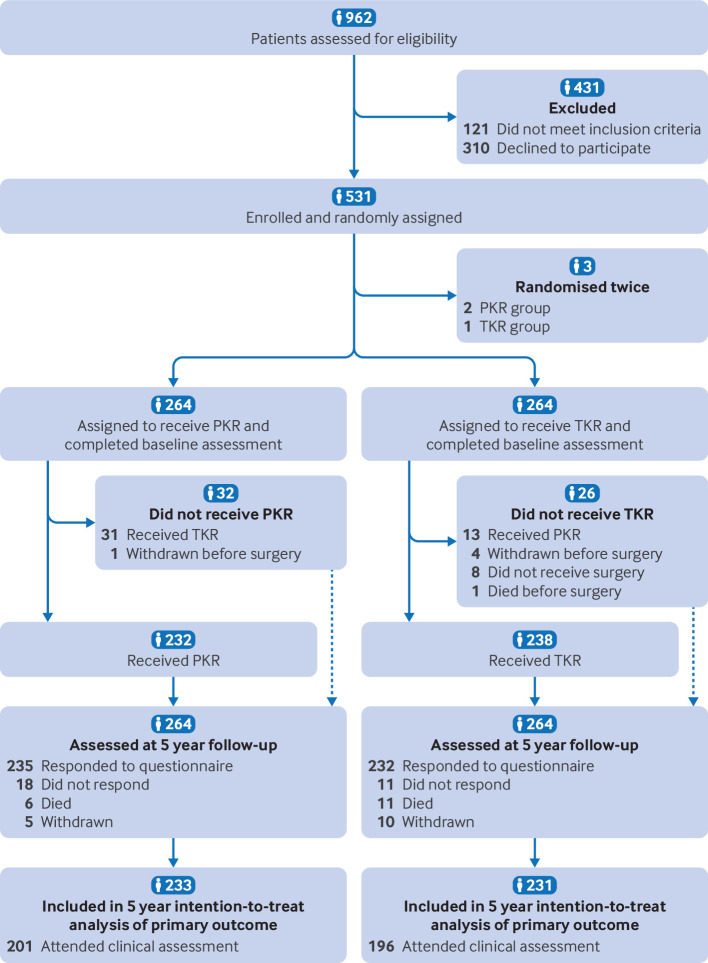
Flow diagram of a multicentre trial of total (TKR) versus partial (PKR) knee replacement^421^

**Fig 3:**
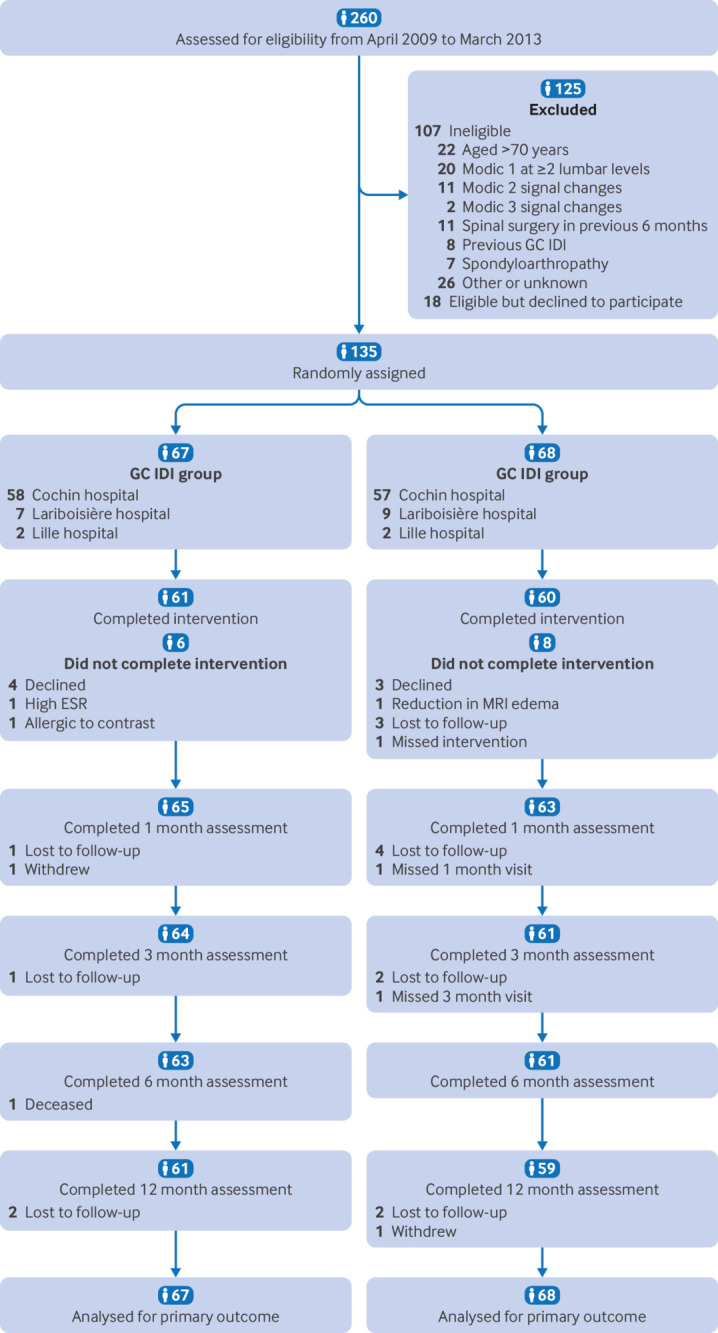
Flow diagram of a multicentre trial of glucocorticoid intradiscal injection in patients with chronic low back pain.^422^ ESR=erythrocyte sedimentation rate; GC IDI=glucocorticoid intradiscal injection; MRI=magnetic resonance imaging

See figure 1, figure 2, and figure 3.

#### Explanation

The design and conduct of some randomised trials are straightforward, and the flow of participants, particularly where there are no losses to follow-up or exclusions, through each phase of the study can be described relatively easily. For other trials, it can be difficult for readers to discern whether and why some participants did not receive the treatment as allocated, were lost to follow-up, or were excluded from the analysis.^423^ This information is crucial for several reasons. Participants who were excluded after allocation are unlikely to be representative of all participants in the study. For example, participants may not be available for follow-up evaluation because they experienced an acute exacerbation of their illness or harms of treatment.^271^ ^424^

Attrition as a result of loss to follow-up, which is often unavoidable, needs to be distinguished from investigator-determined exclusion for such reasons as ineligibility, withdrawal from treatment, and poor adherence to the trial protocol. Erroneous conclusions can be reached if participants are excluded from analysis, and imbalances in such omissions between groups may be especially indicative of bias.^424^ ^425^ ^426^ Information about whether the investigators included in the analysis all participants who underwent randomisation, in the groups to which they were originally allocated (item 21b), is therefore of particular importance. Knowing the number of participants who did not receive the intervention as allocated or did not complete treatment permits the reader to assess to what extent the estimated efficacy of therapy might be underestimated in comparison with ideal circumstances.

If available, the number of people assessed for eligibility, and reason for exclusion, should also be reported. Although this number is relevant to external validity only and is arguably less important than the other counts,^427^ it is a useful indicator of whether trial participants were likely to be representative of all eligible participants.

A suggested template for reporting the number of participants who were randomly assigned, received intended treatment, were lost to follow-up, and were analysed for the primary outcome is shown in figure 1, and the counts required are described in detail in table 7. A review of randomised trials published in general medical journals found that reporting of what happened to participants and their data was considerably more thorough in articles that included a diagram of the flow of participants through a trial than in those that did not.^423^

**Table 7:** Information required to document the flow of participants through each stage of a randomised trial for the primary outcome

Stage	No of people included	No of people not included or excluded	Rationale
Enrolment	People evaluated for potential enrolment	People who did not meet the inclusion criteria or met the inclusion criteria but declined to be enrolled	These counts indicate whether trial participants were likely to be representative of all patients seen; they are relevant to assessment of external validity only, and they are often not available
Randomisation	Participants randomly assigned	—	Crucial count for defining trial size and assessing whether a trial has been analysed by intention-to-treat
Treatment allocation	Participants who received intervention as allocated, by trial group	Participants who did not receive intervention as allocated, by trial group	Important counts for assessment of internal validity and interpretation of results; reasons for not receiving intervention as allocated should be given
Follow-up	Participants who completed intervention as allocated, by trial group Participants who completed follow-up as planned, by trial group	Participants who did not complete intervention as allocated, by trial group Participants who did not complete follow-up as planned, by trial group	Important counts for assessment of internal validity and interpretation of results; reasons for not completing intervention or follow-up should be given
Analysis	Participants included in main analysis for the primary outcome, by trial group	Participants excluded from main analysis for the primary outcome, by trial group	Crucial count for assessing whether a trial has been analysed by intention-to-treat; reasons for excluding participants should be given

Some information, such as the number of individuals assessed for eligibility, may not always be known,^428^ and depending on the nature of a trial, some counts may be more relevant than others. It will sometimes be useful or necessary to adapt the structure of the flow diagram to a particular trial. In some situations, other information may usefully be added. For example, for trials of non-pharmacological interventions it may be important to report the number of care providers or centres performing the intervention in each group and the number of participants treated by each care provider or in each centre.^22^

The exact form and content of the flow diagram may be varied according to specific features of a trial. For example, many trials of surgery or vaccination do not include the possibility of discontinuation. Although CONSORT strongly recommends using a flow diagram to communicate participant flow throughout the study, there is no specific, prescribed format.

### Item 22b: For each group, losses and exclusions after randomisation, together with reasons

#### Example

See figure 2.^421^

#### Explanation

Some protocol deviations may be reported in the flow diagram (item 22a), for example, participants who did not receive the intended intervention. If participants were excluded after randomisation (contrary to the intention-to-treat principle) because they were found not to meet eligibility criteria, they should be included in the flow diagram. Use of the term “protocol deviation” in published articles is not sufficient to justify exclusion of participants after randomisation. The nature of the protocol deviation and the exact reason for excluding participants after randomisation should always be reported. Similarly, if participants did not complete the treatment intervention as allocated (ie, discontinued treatment), this should be reported by trial group, with reasons.

While attrition as a result of loss to follow-up is often unavoidable, it is important to report the number of participants who do not complete follow-up as planned; this should be reported by trial group along with reasons. For the primary outcome, loss to follow-up can be reported in the flow diagram.

### Item 23a: Dates defining the periods of recruitment and follow-up for outcomes of benefits and harms

#### Examples

“Age-eligible participants were recruited . . . from February 1993 to September 1994 . . . Participants attended clinic visits at the time of randomization (baseline) and at 6-month intervals for 3 years.”^429^

“The trial involved five visits: Visit 1 on day 1 (screening, randomization and initial dosing), Visit 2 on day 2 (assessment of the primary endpoint), Visit 3 on day 4 (assessment of efficacy and safety parameters), Visit 4 on day 6 (end-of-treatment visit) and Visit 5 on day 8 to day 10 (follow up by telephone interview). Patients were asked to return all unused trial medication and their diaries at each visit.”^430^ In this example, the term “safety” is used as a reference to harm outcomes; we recommend against the use of “safety”; preferable terms are “harms” or “adverse events.”

#### Explanation

Knowing when a study took place and over what period participants were recruited places the study in historical context. Medical and surgical treatments, including concurrent treatments, evolve continuously and may affect the routine care given to participants during a trial. Thus, it is important to report the periods of recruitment into the trial. Knowing the rate at which participants were recruited may also be useful, especially to other investigators.

The length of follow-up is not always a fixed period after randomisation. In many randomised trials in which the outcome is time to an event, follow-up of all participants is ended on a specific date. This date should be given, and it is also useful to report the minimum, maximum, and median duration of follow-up.^431^ ^432^ A review of reports in oncology journals that used survival analysis, most of which were not randomised trials,^432^ found that nearly 80% (104 of 132 reports) included the starting and ending dates for accrual of patients, but only 24% (32 of 132 reports) also reported the date on which follow-up ended.

Information on the periods of recruitment and follow-up may be different for outcomes of benefits and harm.^20^ For example, the assessment of harms might be planned to take place during the entire study through non-systematic assessment, might occur during only part of the study duration, might occur at specific time points using systematic or non-systematic assessment, or might continue after the completion of follow-up for the main efficacy outcome. Reporting the periods of recruitment and follow-up for benefits and harms is important to allow comprehensive and accurate interpretation of the trial’s results.^20^

### Item 23b: If relevant, why the trial ended or was stopped

#### Examples

“At the time of the interim analysis, the total follow-up included an estimated 63% of the total number of patient-years that would have been collected at the end of the study, leading to a threshold value of 0.0095, as determined by the Lan-DeMets alpha-spending function method . . . At the interim analysis, the RR [risk ratio] was 0.37 in the intervention group, as compared with the control group, with a p value of 0.00073, below the threshold value. The Data and Safety Monitoring Board advised the investigators to interrupt the trial and offer circumcision to the control group, who were then asked to come to the investigation centre, where MC (medical circumcision) was advised and proposed . . . Because the study was interrupted, some participants did not have a full follow-up on that date, and their visits that were not yet completed are described as ‘planned’ in this article.”^433^

“In January 2000, problems with vaccine supply necessitated the temporary nationwide replacement of the whole cell component of the combined DPT/Hib vaccine with acellular pertussis vaccine. As this vaccine has a different local reactogenicity profile, we decided to stop the trial early.”^434^

#### Explanation

Arguably, trialists who arbitrarily conduct unplanned interim analyses after very few events accrue using no statistical guidelines run a high risk of catching the data at a random extreme, which likely represents a large overestimate of treatment benefit.^435^

Readers will likely draw weaker inferences from a trial that was truncated in a data driven manner versus one that reports its findings after reaching a results-independent goal (box 9). Where relevant, authors should report the reason for stopping the trial before completion as planned (eg, result of an interim analysis, lack of funding, poor recruitment of participants, intervention no longer available, or the question becoming no longer relevant after publication of another study). Authors should also disclose factors extrinsic to the trial that affected the decision to stop the trial, and who made the decision to stop the trial, including reporting the role the funding agency had in the deliberations and in the decision to stop the trial.^436^

Box 9 **Early stopping of randomised trials**

Randomised trials can end when they reach their sample size goal, their event count goal, their length of follow-up goal, or their scheduled date of closure. In these situations, the trial will stop in a manner independent of its results and stopping is unlikely to introduce bias in the results. Alternatively, randomised trials can stop earlier than planned because of the result of an interim analysis showing larger than expected benefit or harm of the experimental intervention. Randomised trials can also stop earlier than planned when investigators find evidence of no important difference between experimental and control interventions (ie, stopping for futility). In addition, trials may stop early because the trial becomes unviable: funding vanishes, researchers cannot access eligible patients or study interventions, or the results of other studies make the research question irrelevant.

Full reporting of why a trial ended is important for evidence based decision making (item 23b). Researchers^436^ examining why 143 trials stopped early for benefit found that many failed to report key methodological information regarding how the decision to stop was reached: the planned sample size (n=28), interim analysis after which the trial was stopped (n=45), or whether a stopping rule informed the decision (n=48). Item 16b of the CONSORT checklist requires the reporting of timing of interim analyses, what triggered them, how many took place, whether these were planned or ad hoc, and whether there were statistical guidelines and stopping rules in place a priori. Furthermore, it is helpful to know whether an independent data monitoring committee participated in the analyses (and who composed it, with particular attention to the role of the funding source), and who made the decision to stop. Often the data monitoring committee make recommendations and the funders (sponsors) or the investigators make the decision to stop.

Trials that stop early for reasons apparently independent of trial findings, and trials that reach their planned termination, are unlikely to introduce bias by stopping.^437^ In these cases, the authors should report whether interim analyses took place and whether these results were available to the funder.

The push for trials that change the intervention in response to interim results, thus enabling a faster evaluation of promising interventions for rapidly evolving and fatal conditions, will require even more careful reporting of the process and decision to stop trials early.^174^ ^438^

CONSORT=Consolidated Standards of Reporting Trials.

A systematic review of 143 randomised trials that were stopped earlier than planned for benefit found that these trials reported stopping after accruing a median of 66 events. The review estimated a median relative risk of 0.47 and a strong association between the number of events accrued and the magnitude of the effect, with smaller trials with fewer events yielding the largest treatment effects (odds ratio 31, 95% CI 12 to 82).^436^ While an increasing number of trials published in high impact medical journals report stopping early, many still do not report how the decision to stop the trial was made. In a systematic review of 110 paediatric trials that reported on the presence of a data monitoring committee, interim analysis, or early stopping, 32 were terminated early. Of these 32 trials, 22 (69%) did not report predefined stopping guidelines and 15 (47%) did not provide information on statistical monitoring methods.^439^

### Item 24a: Intervention and comparator as they were actually administered (eg, where appropriate, who delivered the intervention/comparator, whether participants adhered, whether they were delivered as intended (fidelity))

#### Examples

“Patients were randomly assigned to the P2Y12 inhibitor monotherapy group (aspirin plus a P2Y12 inhibitor for 3 months and thereafter a P2Y12 inhibitor alone) or to the DAPT group (aspirin plus a P2Y12 inhibitor for at least 12 months) in a 1:1 ratio . . . Overall adherence to the study protocol was 79.3% in the P2Y12 inhibitor monotherapy group and 95.2% in the DAPT group . . . The rates of P2Y12 inhibitor use were similar in both groups: 96.4% at 6 months and 95.0% at 12 months in the P2Y12 inhibitor monotherapy group and 98.1% at 6 months and 96.6% at 12 months in the DAPT group. The median duration of aspirin was 96 days (interquartile range, 88-118 days) in the P2Y12 inhibitor monotherapy group and 365 days (interquartile range, 363-365) in the DAPT group. The proportion of patients receiving aspirin beyond 3 months in the P2Y12 inhibitor monotherapy group was 14.4% at 6 months and 8.9% at 12 months.”^440^

“Most participants received treatment as allocated [table 8]. Across intervention groups, high protocol adherence was achieved in terms of the delivery, type, and content for the injection, progressive exercise, and best practice advice interventions. 53 physiotherapists delivered corticosteroid injections to 329 (97%) participants and three doctors to ten (3%) participants. Progressive exercise was delivered by 104 physiotherapists to 339 participants and best practice advice was delivered by 83 physiotherapists to 324 participants. Two physiotherapists swapped groups during the trial because of staffing issues and delivered both interventions. We found no difference in attendance rates between those receiving progressive exercise or best practice advice and those who received the intervention in conjunction with corticosteroid injection [table 8].”^114^

**Table 8:** Example of good reporting: Intervention received by treatment group

	Best practice advice (n=174)	Injection and best practice advice (n=178)	Progressive exercise (n=174)	Injection and progressive exercise (n=182)
Injection received	—	168 (94)	—	171 (94)
Injection not received with reasons	—	10 (6)	—	11 (6)
Received extra injection	—	0	—	2 (1)
Completed exercise treatment	162 (93)	162 (91)	138 (79)	139 (76)
Partial exercise completion	—	—	29 (17)	33 (18)
Median (IQR) number of sessions	1 (1-1)	1 (1-1)	4 (3-6)	4 (3-5)
Completed session 1	162 (93)	162 (91)	167 (96)	172 (95)
Completed session 2	—	—	161 (93)	160 (88)
Participants who received additional sessions	3 (2)	5 (3)	3 (2)	2 (1)

Data are number (%) or participants unless stated otherwise. Adapted from Hopewell et al, 2021^114^

IQR=interquartile range.

#### Explanation

This new item has been added to the CONSORT 2025 checklist to address the poor reporting of the intervention and comparator in randomised trials.^195^ ^198^ ^199^ ^200^ ^201^ ^214^ For example, in a review of 102 randomised trials evaluating bariatric surgery, only 14% reported the intervention as implemented.^441^ A review of 192 randomised trials assessing pharmacological treatments in six major chronic diseases published in journals with high impact factors showed that adherence to medication was reported in only one third of the publications.^442^ A review of 100 randomised trial reports published in general medical journals with high impact factors highlighted that only 11% of the trials assessing long term interventions and 38% of those assessing short term interventions adequately reported treatment initiation and completeness of treatment.^209^ A review of 111 randomised trials reports showed that only 46% reported adherence results.^443^ A review of 94 placebo/sham controlled randomised trials published in high impact journals showed that only 54% reported actual adherence or fidelity.^214^

There is frequently a gap between the intervention/comparator as planned and described in the trial protocol and how the intervention/comparator were actually administered. This gap could be related to poor fidelity, which can be driven by the extent to which the intervention/comparator are implemented as planned in the protocol by practitioners, and/or poor adherence to treatment, defined as the extent to which participants comply with the care providers’ recommendations (eg, taking a drug, placebo, behavioural change, doing exercises).^209^ ^444^ This gap could also be related to the expected diversity in the implementation of the intervention/comparator in clinical practice particularly for complex interventions.

The gap between the intervention/comparator as planned and as delivered also depends on how the trial was planned. In explanatory trials, the aim is to estimate treatment effect under ideal circumstances. The intervention/comparator are usually highly standardised with close monitoring of fidelity and adherence to interventions and strategies to increase them. Intensive efforts to maximise fidelity and adherence in early phase trials or explanatory trials may lead to unrealistic, inflated estimates of treatment benefit that cannot be reproduced under real life circumstances.^445^ ^446^ Reporting the results of this monitoring is essential to allow readers to interpret the study results.

In contrast, pragmatic trials aim to determine treatment effect in clinical conditions. The intervention and comparator are usually highly flexible, and measurement of fidelity and adherence is unobstructive with no strategies to maintain or improve them.

Reporting how the intervention/comparator were actually administered is nevertheless crucial to allow readers to accurately interpret the trial results. For example, in a large international randomised trial comparing endarterectomy to medical management for patients with symptomatic carotid artery stenosis, there were important differences in the delay in receiving the surgical procedure which impacted the outcomes.^179^

Authors should provide details on who actually delivered the intervention/comparator (number and expertise), how the intervention/comparator were delivered, what was actually administered, participants’ adherence to treatment, and the caregiver’s fidelity to the intervention/comparator protocol where appropriate. Reporting fidelity and adherence can be complex and vary according to the type of intervention or comparator (eg, one-off, short term repeated, long term repeated). Various deviations to the protocol can occur. Participants might initiate the intervention/comparator but then discontinue the intervention/comparator permanently and completely after a specific period of time, discontinue temporarily, reduce the dose, or modify the schedule. 

More detailed information is available in TIDieR^23^ and the CONSORT extension for non-pharmacological treatments^22^ (item 13).

### Item 24b: Concomitant care received during the trial for each group

#### Example

“The principal investigators invited hospitals with the capability to provide the current standard of care for covid-19 to participate in the study. Minimum requirements for the standard of care included the provision of intravenous fluids, supplemental oxygen, regular laboratory testing, SARS-CoV-2 testing, haemodynamic monitoring, and intensive care, as well as the ability to deliver concomitant medications . . .

“90 (60%) patients received concomitant drug treatment before randomisation. Among these, 52 (35%) patients received antiviral treatment [table 9] . . . Concomitant treatments, including antiviral agents, antibiotics, and systemic glucocorticoid therapy, were similar in the two groups [table 9].”^447^

**Table 9:** Example of good reporting: Treatments after randomisation in patients in an intention-to-treat population

Drug treatment after randomisation	Standard of care plus hydroxychloroquine (n=75)	Standard of care (n=75)	Total (n=150)
Antiviral agents	47 (63)	48 (64)	95 (63)
Arbidol	37 (49)	33 (44)	70 (47)
Virazole	13 (17)	15 (20)	28 (19)
Lopinavir-ritonavir	13 (17)	12 (16)	25 (17)
Oseltamivir	8 (11)	9 (12)	17 (11)
Entecavir	1 (1)	1 (1)	2 (1)
Antibiotics	32 (43)	27 (36)	59 (39)
Systemic glucocorticoid treatment	6 (8)	4 (5)	10 (7)

Data are number (%). Adapted from Tang et al.^447^

#### Explanation

Concomitant care refers to any additional treatments, interventions, or medications that participants may have received during the trial period, in addition to the study interventions. Relevant concomitant care refers to interventions that could have affected the outcome. Transparently reporting this information is essential for readers to understand the context in which the trial was conducted and be able to assess the potential influence of concomitant care on the trial’s results. Particularly, readers should be aware of any unequal use of concomitant care that might affect the outcome between the intervention and comparator groups.^448^ This is particularly important when the trials are not fully blinded.^449^ This information could be particularly important for evaluating the risk of bias due to deviations from the intended interventions, an important domain of the risk-of-bias tool developed by Cochrane.^210^ ^211^

Nevertheless, this information is poorly reported. A review of 164 cardiovascular clinical trials published in five influential medical journals from 2011 to 2021 showed that cointerventions were inadequately reported in 71% of the trials 450 and that trials with deficient reporting had larger treatment effect estimates on average.^451^ In rheumatology, an assessment of 109 trials in leading journals from 2018 to 2020 found that only 57% of randomised trials provided the number of patients on concomitant medications at baseline, and only 5% reported the cumulative or mean exposure data for concomitant medications.^452^

Authors should report the number and percentage of participants receiving the different relevant concomitant care in each arm and, where relevant, the cumulative or average for each concomitant intervention taken over the trial period for each group.

### Item 25: A table showing baseline demographic and clinical characteristics for each group

#### Example

See table 10.^453^

**Table 10:** Example of good reporting: Baseline demographic and clinical characteristics between study groups

Characteristic	Usual care (n=242)	Exercise (n=246)
Mean (SD) age (years)	63.5 (11)	61.3 (12)
Female sex	186 (76)	188 (76)
Ethnic origin		
White	235 (98)	238 (97)
Indian	2 (1)	3 (1)
Pakistani	1 (<1)	—
Mixed	1 (<1)	3 (1)
Other	1 (<1)	2 (1)
Dominant in right hand	215 (90)	226 (92)
Median (IQR) No of years since rheumatoid arthritis diagnosis, estimated by participant	10 (4-22)	10 (4-21)
Median (IQR) baseline ESR	16 (8-28)	15 (7-28)
Median (IQR) baseline CRP	6 (3-12)	5 (3-12)
Drug treatment		
Biological DMARD	52 (22)	51 (21)
Combination non-biological DMARD	53 (22)	72 (29)
Single non-biological DMARD	118 (49)	103 (42)
Other drugs	19 (8)	19 (8)
Mean (SD) MHQ		
Overall hand function (both)	52.1 (16.4)	52.1 (15.2)
Activities of daily living (both)	54.1 (25.0)	54.5 (24.5)
Work	48.4 (22.0)	48.2 (22.0)
Pain	51.4 (19.9)	51.9 (21.9)
Aesthetics (both)	58.6 (22.1)	56.9 (22.0)
Satisfaction (both)	43.5 (22.3)	43.9 (19.7)
Overall score	50.9 (16.9)	50.6 (16.4)
Mean (SD) SF-12 score		
Aggregate physical scale (PCS)	34.5 (9.5)	33.8 (9.8)
Aggregate mental scale (MCS)	48.9 (11.0)	48.1 (10.7)

Data are number (%) of participants unless stated otherwise. DMARD=disease-modifying drugs; ESR=erythrocyte sedimentation rate; IQR=interquartile range; MHQ=Michigan Hand Outcome Questionnaire; PCS=physical component score; MCS=mental component score; SD=standard deviation. Adapted from Lamb et al.^453^

#### Explanation

Although the eligibility criteria (item 12a) indicate who was eligible for the trial, it is also important to know the characteristics of the participants who were actually included. This information allows readers, especially clinicians, to judge how relevant the results of a trial might be to an individual patient. Participant baseline demographics may include characteristics such as age, sex and/or gender,^182^ place of residence, race and/or ethnicity, culture and/or religion, language, occupation, education, or socioeconomic status. Baseline clinical characteristics include those which are identical, or closely related, to the trial outcomes.^454^

Randomised trials aim to compare groups of participants that differ only with respect to the intervention (treatment). Although proper random assignment prevents selection bias, it does not guarantee similarity of the groups at baseline. Any differences in baseline characteristics are, however, the result of chance rather than bias.^294^ Important demographic and clinical characteristics should be presented so that readers can assess how similar the groups were at baseline. Baseline data are especially valuable for outcomes that can also be measured at the start of the trial (eg, blood pressure).

Baseline information is most efficiently presented in a table. For continuous variables, such as weight or blood pressure, the variability of the data should be reported, along with average values. Continuous variables can be summarised for each group by the mean and standard deviation. When continuous data have an asymmetrical distribution, a preferable approach may be to quote the median and percentile values (eg, the 25th and 75th percentiles).^365^ Standard errors and CIs are not appropriate for describing variability—they are inferential rather than descriptive statistics. Variables with a small number of ordered categories (such as stages of disease I to IV) should not be treated as continuous variables; instead, numbers and proportions should be reported for each category.^364^ ^365^

Significance testing of baseline differences is not recommended and should not be reported.^269^ ^294^ ^455^ Such significance tests assess the probability that observed baseline differences could have occurred by chance; however, providing the randomisation has not been subverted or comprised, any differences are caused by chance. Unfortunately, such significance tests are still relatively common.^456^ ^457^ ^458^ Such hypothesis testing is superfluous and can mislead investigators and their readers.^459^ Rather, comparisons at baseline should be based on consideration of the prognostic strength of the variables measured and the magnitude of any chance imbalances that have occurred.^459^

### Item 26: Items for each primary and secondary outcome

For each primary and secondary outcome, by group:

- the number of participants included in the analysis
- the number of participants with available data at the outcome time point
- result for each group, and the estimated effect size and its precision (such as 95% CI)
- for binary outcomes, presentation of both absolute and relative effect size.

#### Examples

“All principal analyses were based on the intention-to treat (ITT) principle, analysing participants in the groups to which they were randomly assigned irrespective of compliance with treatment allocation.”^460^

See table 11,^123^ table 12,^123^ and table 13.^461^

**Table 11:** Example of good reporting: Summary results for each trial group (binary outcomes). Secondary outcomes of arm symptoms, and lymphoedema by treatment group. Data are number (%) unless stated otherwise

Outcome	Usual care	Exercise	Adjusted odds ratio (95% CI)
Acute and chronic postoperative pain*			
Moderate to severe, 6 weeks	46/150 (31)	28/153 (18)	1.90 (1.02 to 3.52)
Moderate to severe, 6 months	30/133 (23)	25/145 (17)	1.42 (0.72 to 2.84)
Moderate to severe, 12 months	43/139 (31)	22/135 (16)	2.41 (1.24 to 4.70)
Neuropathic pain, DN4 positive			
6 weeks	21/150 (14)	24/153 (16)	0.73 (0.22 to 2.45)
6 months	29/133 (22)	26/145 (18)	1.64 (0.63 to 4.23)
12 months	32/139 (23)	22/135 (16)	1.29 (0.45 to 3.69)
Lymphoedema, LBCQ			
6 weeks	20/150 (13)	22/153 (14)	1.07 (0.52 to 2.24)
6 months	32/133 (24)	29/145 (20)	0.82 (0.43 to 1.56)
12 months	36/139 (26)	33/135 (24)	1.17 (0.62 to 2.23)

DN4=Douleur Neuropathique-4 (positive neuropathic pain=score >3); LBCQ=Lymphoedema and Breast Cancer Questionnaire (positive symptoms=arm both heavy and swollen). Adapted from Bruce et al.^123^

* Numerical rating scale: acute and chronic post-operative pain. Moderate to severe pain=4-10.

**Table 12:** Example of good reporting: Summary results for each trial group (continuous outcomes). Disability of Arm, Shoulder, Hand (DASH) scores by treatment group

Time point, analysis	Usual care			Exercise			Difference between groups (95% CI)*	
	No	Mean (SD)		No	Mean (SD)		Unadjusted	Adjusted
6 months, ITT	125	20.8 (20.1)		134	18.0 (17.1)		2.76 (−1.79 to 7.31)	4.60 (0.30 to 8.90)
12 months, ITT (primary outcome)	138	23.7 (22.9)		132	16.3 (17.6)		7.34 (2.44 to 12.23)	7.81 (3.17 to 12.44)

Scores adjusted for age, baseline DASH, breast surgery, axillary surgery, radiotherapy, and chemotherapy. Mean differences in upper limb function favour exercise intervention. Adapted from Bruce et al.^123^

CI=confidence interval; ITT=intention to treat; SD=standard deviation.

* Absolute mean difference between treatment groups.

**Table 13:** Example of good reporting: Absolute and relative effect sizes

Primary outcome	Early administration (%) (n=1344)	Delayed selective administration (%) (n=1346)	Risk ratio (95% CI)	Risk difference (95% CI)
Death or oxygen dependence at “expected date of delivery”	31.9 (429)	38.2 (514)	0.84 (0.75 to 0.93)	−6.3 (−9.9 to −2.7)

Data are % (number) of participants, unless stated otherwise. Adapted from Sola et al.^461^ “The risk of oxygen dependence or death was reduced by 16% (95% CI 25% to 7%). The absolute difference was −6.3% (95% CI −9.9% to −2.7%); early administration to an estimated 16 babies would therefore prevent 1 baby dying or being long-term dependent on oxygen.”^461^

CI=confidence interval.

#### Explanation

For each primary and secondary outcome, the number of participants included in each group is an essential element of the analyses. Although the flow diagram (item 22a) should indicate the numbers of participants included in the analysis of the primary outcome, the number of participants with available data will often vary for different outcomes and at different time points.

Missing data can introduce potential bias through different types of participants being included in each treatment group. It can also reduce, through loss of information, the power to detect a difference between treatment groups if one exists (item 21c) and reduce the generalisability of the trial findings.^404^ It is therefore important to report the number of participants with available data for each primary and secondary outcome and at each timepoint. Where possible, it is also important to report the reason data were not available, for example, if the participant did not attend follow-up appointments, or if data were truncated because the participant died.^404^ The extent and causes of missing data can vary. For example, a systematic review of palliative care trials estimated that 23% of primary outcome data were not available^462^; this compares to a recent review of trials published in four top general medical journals where the median percentage of participants with a missing outcome was around 9%.^392^

Trial results are often more clearly displayed in a table rather than in the text, as shown in table 11 and table 12. For each outcome, results should be reported as a summary of the outcome in each group (eg, the number of participants included in the analysis with or without the event and the denominators, or the mean and standard deviation of measurements), together with the contrast between the groups, known as the effect size. For binary outcomes, the effect size could be the risk ratio (relative risk), odds ratio, or risk difference; for survival time data, it could be the hazard ratio or difference in median survival time; and for continuous data, it is usually the difference in means.

For all outcomes, authors should provide a CI to indicate the precision (uncertainty) of the estimated effect size.^364^ ^463^ A 95% CI is conventional, but occasionally other levels are used. Most journals require or strongly encourage the use of CIs.^464^ They are especially valuable in relation to differences that do not meet conventional statistical significance, for which they often indicate that the result does not rule out an important clinical difference. The use of CIs has increased markedly in recent years, although not in all medical specialties.^465^ A common error is the presentation of separate CIs for the outcome in each group rather than for the treatment effect.^465^ Although P values may be provided in addition to CIs, results should not be reported solely as P values.^466^ ^467^ Results should be reported for all planned primary and secondary outcomes and at each time point, not just for analyses that were statistically significant or thought to be interesting. Selective reporting within studies is a widespread and serious problem.^51^ ^468^

When the primary outcome is binary, both the relative effect (risk ratio (relative risk) or odds ratio), and the absolute effect (risk difference) should be reported (with CIs) (table 13), as neither the relative measure nor the absolute measure alone gives a complete picture of the effect and its implications. Different audiences may prefer either relative or absolute risk, but both clinicians and lay people tend to overestimate the effect when it is presented solely in terms of relative risk.^469^ ^470^ ^471^ The magnitude of the risk difference is less generalisable to other populations than the relative risk since it depends on the baseline risk in the unexposed group, which tends to vary across populations. For diseases where the outcome is common, a relative risk near unity might nonetheless indicate clinically important differences in public health terms. In contrast, a large relative risk when the outcome is rare may not be so important for public health (although it may be important to an individual in a high risk category). For both binary and survival time data, expressing the results also as the number needed to treat for benefit or harm can be helpful.^472^ ^473^

### Item 27: All harms or unintended events in each group

#### Example

“Few women vomited after drug administration. 12 (0.2%) of 6685 sulfadoxine–pyrimethamine, 19 (0.3%) of 7014 dihydroartemisinin–piperaquine, and 23 (0.3%) of 6849 dihydroartemisinin–piperaquine plus azithromycin treatment courses were vomited within 30 min [table 14]. One (0.1%) of 1552 women in the sulfadoxine–pyrimethamine group, two (0.1%) of 1558 in the dihydroartemisinin–piperaquine group, and four (0.3%) of 1556 in the dihydroartemisinin–piperaquine plus azithromycin group vomited after their first course of treatment (the only course when azithromycin was coadministered with dihydroartemisinin–piperaquine; table 14). All three regimens were well tolerated ([table 14] . . . ), but vomiting, nausea, and dizziness were more common in the first 3 days after dihydroartemisinin–piperaquine (13 [3.2%], 14 [3.4%], and 15 [3.7%] of 410 women visited at home, respectively) than sulfadoxine–pyrimethamine (one [0.3%], one [0.3%], and zero [0%] of 384 women visited at home, respectively; appendix pp 20–21). The addition of azithromycin to dihydroartemisinin–piperaquine was associated with significantly more vomiting than with dihydroartemisinin–piperaquine alone (p=0.0033; [table 14]).”^196^

**Table 14:** Example of good reporting: Safety and tolerability endpoints (incidence measures, events (incidence per person year at risk) or prevalence. Data are number (%) unless stated otherwise

Endpoint	Treatment		
	Sulfadoxine-pyrimethamine	Dihydroartemisinin-piperaquine	Dihydroartemisinin-piperaquine plus azithromycin
**Adverse events**			
Dizziness	4 (0.8)	51 (9.5)	38 (7.2)
Vomiting	4 (0.8)	41 (7.7)	71 (13.5)
Nausea	2 (0.4)	44 (8.2)	35 (6.7)
Abdominal pain	6 (1.1)	11 (2.1)	14 (2.7)
Diarrhoea	2 (0.4)	5 (0.9)	6 (1.1)
Headache	12 (2.3)	17 (3.2)	18 (3.4)
Rash	0 (0.0)	2 (0.4)	0 (0.0)
**Serious adverse events and grade 3-4 adverse events (in pregnant women)**			
Any	95 (17.7)	79 (14.8)	92 (16.9)
Maternal mortality*	1/1553 (0.1)	2/1561 (0.1)	3/1557 (0.2)
**By system organ class**			
Blood and lymphatic system disorders	2 (0.4)	0 (0.0)	2 (0.4)

Adapted from Madanitsa et al.^196^

* Number (%)/total number.

#### Explanation

Readers need information about the harms as well as the benefits of interventions to make rational and balanced decisions. Randomised trials offer an excellent opportunity for providing harms data, although they cannot detect differences in uncommon or rare harms between treatment groups. The existence and nature of adverse effects can have a major impact on whether a particular intervention will be deemed acceptable and useful. Not all reported adverse events observed during a trial are necessarily a consequence of the intervention; some may be a consequence of the condition being treated. Nevertheless, they all need to be reported.

Many reports of randomised trials provide inadequate information on harms. A comparison between harm data submitted to the trials database of the National Cancer Institute, which sponsored the trials, and the information reported in journal articles found that low grade adverse events were under-reported in journal articles. High grade events (Common Toxicity Criteria grades 3 to 5) were reported inconsistently in the articles and the information regarding attribution to investigational drugs was incomplete.^474^ Moreover, a review of trials published in six general medical journals in 2006 to 2007 found that while 89% of 133 reports mentioned adverse events, no information on severe adverse events and withdrawal of patients owing to an adverse event was given in 27% and 48% of articles, respectively.^475^ In a later review of 196 randomised trials of invasive pain treatments published in six major journals, 76% provided the denominators for analyses on harms and 85% reported the absolute risk per arm and per adverse event type, grade, and seriousness, and presented appropriate metrics.^476^

For non-systematically assessed harms, reporting can be more complex as the information is not standardised. A common approach is to code the event declared by participants. Authors should report the coding system used, whether coding was prespecified in the protocol, in the statistical analysis plan, or post hoc, and whether coding was performed by researchers blinded to the treatment allocated. In addition, there is a risk of under-reporting and selective non-reporting of harms particularly for non-systematically assessed harms. A reanalysis of individual participant data from six randomised trials of gabapentin found evidence of important harms that were not disclosed in the published reports but identified after data sharing and reanalysis.^477^ Sharing of de-identified individual participant data may be needed to be able to adequately synthesise this information, for example for inclusion in a systematic review.^231^ ^235^ ^236^

Authors should report for each group, the number of participants at risk, the number of deaths, the number of participants withdrawn due to harms, the number of participants with at least one harm event, and the number of events, if appropriate. Where appropriate, the estimated effect size with its precision (such as 95% CIs) should be reported including both absolute and relative effects for binary outcomes. It is important to separate the reporting of systematically and non-systematically assessed harms. Systematically assessed harms should be reported even if zero events were identified. It should also be clear whether the authors are reporting the number of participants with at least one harm event or the number of events per unit of time at risk and whether recurrent events were included. The number of participants withdrawn because of harms should also be reported for each group. Finally, results should be reported for all harms. We strongly discourage the use of thresholds or criteria to select which harms should be reported. All harms could be detailed in supplementary materials.

We recommend reporting the results in a table with the results for each trial arm.^478^ More detailed information can be found in the CONSORT statement extension for harms, which was updated in 2022.^20^

### Item 28: Any other analyses performed, including subgroup and sensitivity analyses, distinguishing prespecified from post hoc

#### Examples

“In a [prespecified] sensitivity analysis to support the primary binary endpoint, the NRS [numerical rating score] pain score at 1 month was also analyzed using the constrained longitudinal data analysis model . . . Primary Outcome: At 1 month after the intervention, the percentage of responders (Low Back Pain intensity <40) was higher in the glucocorticoid intradiscal injection (GCIDI) group (36 of 65 [55.4%]) than the control group (21of 63 [33.3%]) (absolute risk difference, 22.1 percent-age points [CI, 5.5 to 38.7 percentage points]; P=0.009 [after multiple imputation]) . . . In the sensitivity analysis, the mean reduction in LBP [low back pain] intensity from baseline to 1 month was greater in the GCIDI group (−32.5 [CI,-38.2 to −26.8]) than the control group (−17.5 [CI, −23.3 to −11.7]) (absolute difference, -15.0 [CI,-22.9 to −7.1]; P< 0.001).”^422^

“Owing to the later inclusion of parent cosmetic appearance assessments (to assist with trial conduct), it was decided to perform a post hoc subgroup analysis to determine whether the scores given by the assessors and parents differed between treatment groups [table 15] . . . The assessor scores did not indicate a difference between the nail-replaced and nail-discarded groups. However, the scores given by the parents suggested that there was a statistically significant difference in favour of the nail-discarded group. The treatment by subgroup interaction term was statistically significant (OR [odds ratio] 0.24, 95% CI [confidence interval] 0.06 to 0.96. P= 0.044).”

**Table 15:** Example of good reporting: Main, secondary, and subgroup analyses of Oxford Finger Nail Appearance Score cosmetic outcome^479^

	Nail replaced	Nail discarded	Effect size*
Main analysis			
OFNAS, median (IQR)	5 (4-5)	5 (4-5)	0.55 (0.49 to 0.60)†
Subgroup analyses			
Assessor (parent *v* child)			0.24, (0.06 to 0.96)‡
Preoperative antibiotic use			1.11 (0.62 to 2.31)‡

Adapted from Jain et al.^479^

IQR=interquartile range; OFNAS=Oxford Finger Nail Appearance Score.

* Values in parentheses are 95% confidence intervals; effect sizes are shown as odds ratios, except †probability that OFNAS in discard arm is greater than that in replace arm from Mann-Whitney U test.

‡ Adjusted model allowed for intrasite correlation using cluster-robust standard errors.

#### Explanation

Multiple analyses of the same data create a risk for false-positive findings.^480^ Authors should especially resist the temptation to perform many subgroup analyses.^481^ ^482^ ^483^ Analyses that were prespecified in the trial protocol (item 3) are much more reliable than those suggested by the data, and therefore authors should report which analyses were prespecified. If subgroup analyses were undertaken, authors should report which subgroups were examined, why, whether they were prespecified, and how many were prespecified. Selective reporting of subgroup analyses could lead to bias.^484^ When evaluating a subgroup, the question is not whether the subgroup demonstrates a statistically significant result but whether the subgroup treatment effects are significantly different from each other. To determine this, a test of interaction is helpful, although the power for such tests is typically low. If formal evaluations of interaction are undertaken (item 21d) they should be reported as the estimated difference in the intervention effect in each subgroup (with a CI), not just as P values.

In one survey,^481^ 35 of 50 trial reports included subgroup analyses, of which only 42% used tests of interaction. It was often difficult to determine whether subgroup analyses had been specified in the protocol. In another survey of surgical trials published in high impact journals, 27 of 72 trials reported 54 subgroup analyses of which 91% were post hoc and only 6% of subgroup analyses used a test of interaction to assess whether a subgroup effect existed.^485^

## CONSORT 2025: Discussion

### Item 29: Interpretation consistent with results, balancing benefits and harms, and considering other relevant evidence

#### Example

“In this trial, which compared standard of care with monthly IPTp [intermittent preventive treatment in pregnancy] with sulfadoxine–pyrimethamine with monthly IPTp with dihydroartemisinin–piperaquine and monthly IPTp with dihydroartemisinin–piperaquine plus azithromycin, the use of dihydroartemisinin– piperaquine was associated with reductions in clinical malaria, malaria infection detected by microscopy during pregnancy, and placental malaria at the time of birth. However, despite these reductions in malaria, the risk of adverse pregnancy outcomes (composite primary endpoint) was significantly lower in the sulfadoxine– pyrimethamine group than in both dihydroartemisinin–piperaquine groups . . .

“In a previous meta-analysis a modest, but nonsignificant, reduction of 17% in the number of adverse pregnancy outcomes favouring dihydroartemisinin– piperaquine versus sulfadoxine–pyrimethamine was reported . . . These findings led WHO [World Health Organization] to recommend larger definitive studies to determine whether IPTp with dihydroartemisinin–piperaquine improves adverse pregnancy outcomes compared with sulfadoxine– pyrimethamine in areas with high sulfadoxine–pyrimethamine resistance. Our results are consistent with those from a third trial in eastern Uganda, which showed that monthly IPTp with dihydroartemisinin– piperaquine did not decrease the number of adverse pregnancy outcomes among livebirths or reduce fetal loss compared with monthly IPTp with sulfadoxine–pyrimethamine . . . All of these trials, including ours, show that dihydroartemisinin– piperaquine is the superior antimalarial, resulting in 40–90% fewer malaria infections during pregnancy.”^196^

#### Explanation

A good discussion section of a completed randomised trial should start with a brief summary of the trial results, balancing both benefits and harms of the intervention. The discussion sections of scientific reports are often filled with rhetoric supporting the authors’ findings^486^ and provide little measured argument of the pros and cons of the study and its results. Indeed, some discussion sections can be overly optimistic when discussing the trial findings (interpretation bias; spin).^33^ ^487^ ^488^ ^489^ Authors need to guard against such behaviours, as they diminish the rigour of the scientific effort and may result in a loss of trust by readers.

Readers will also want to know how the trial results relate to those of other randomised trials. This can best be achieved by including a published systematic review in the results or discussion section of the report.^490^ ^491^ This might be an easy ask for some authors as a systematic review might have been part of the rationale for conducting the trial. However, for others, such synthesis may be impractical, and quoting and discussing any existing systematic reviews of similar trials may be more appropriate. One recent estimate suggests that nearly 80 systematic reviews are published daily.^492^ Discussing trial findings in the context of results from any systematic reviews will help readers assess whether the results of the randomised trial are similar to those of other trials in the same topic area and whether participants are similar across studies. Reports of randomised trials have often not dealt adequately with these points.^490^ Several methods to address the issue of setting new trial findings within the context of previous research have been proposed.^491^ Where conducting a new updated systematic review is not practical, adding the new trial result to the previous systematic review is a much simpler alternative.

We recommend that at a minimum, the discussion should be as systematic and objective as possible and be based on a comprehensive search, rather than being limited to studies that support the results of the current trial.^493^ ^494^

### Item 30: Trial limitations, addressing sources of potential bias, imprecision, generalisability, and, if relevant, multiplicity of analyses

#### Examples

“The preponderance of male patients (85%) is a limitation of our study . . . We used bare-metal stents, since drug-eluting stents were not available until late during accrual. Although the latter factor may be perceived as a limitation, published data indicate no benefit (either short-term or long-term) with respect to death and myocardial infarction in patients with stable coronary artery disease who receive drug-eluting stents, as compared with those who receive bare-metal stents.”^495^

“Our study had several limitations. The early changes to the protocol to accommodate patients with a shorter injury history (but still not acute) to improve recruitment altered the characteristics of the study population. Overall, patients had less long-standing injury than was originally planned. Moreover, the study addressed a deliberately specific population of patients who continued to have ACL injury-related symptoms of instability and had not undergone any previous formal treatment. Another potential limitation is the proportion of patients who did not undergo surgical reconstruction, despite allocation to that group. The true benefit of surgical reconstruction could be somewhat greater than the ITT analysis suggests. The 18-month follow-up period ideally could have been longer but was constrained by various factors including funding. Notwithstanding, most patients had established their level of instability at this timepoint since being included in the trial. The trial design and analysis accounted for delayed surgery in both groups.”^460^

#### Explanation

An essential part of a good discussion section is summarising the limitations of the trial. Limitations are frequently omitted from research reports^496^; identification and discussion of the weaknesses of a study have particular importance.

Some journals have attempted to remedy this problem by encouraging more structure to authors’ discussion of their results.^497^ ^498^ For example, *The BMJ* recommends that authors structure the discussion section by presenting (1) a statement of the principal findings; (2) the strengths and weaknesses of the study; (3) the strengths and weaknesses in relation to other studies, discussing important differences in results; (4) the meaning of the study, its possible explanations and implications for clinicians and policymakers; and (5) any unanswered questions and future research.^499^ We recommend that authors follow these sensible suggestions, perhaps also using suitable subheadings in the discussion section.

Authors should also discuss any imprecision of the results. Imprecision may arise in connection with several aspects of a study, including measurement of a primary outcome (item 14) or diagnosis (item 12a). Perhaps the scale used was validated on an adult population but used in a paediatric one, or the assessor was not trained in how to administer the instrument.

The difference between statistical significance and clinical importance should always be borne in mind. Authors should particularly avoid the common error of interpreting a non-significant result as indicating equivalence of interventions. The CI (item 26) provides valuable insight into whether the trial result is compatible with a clinically important effect, regardless of the P value.^500^

Authors should exercise special care when evaluating the results of trials with multiple comparisons. Such multiplicity arises from several interventions, outcome measures, time points, subgroup analyses, and other factors. In such circumstances, some statistically significant findings are likely to result from chance alone.

Authors should also consider the extent to which the results of a study can be generalised to other circumstances; also known as external validity. For example, can the results be generalised to an individual participant or groups that differ from those enrolled in the trial with regard to age, sex, severity of disease, and comorbid conditions? Are the results applicable to other drugs within a class of similar drugs, to a different dose, timing, and route of administration? Can similar results be expected in different healthcare settings? What about the effect on related outcomes that were not assessed in the trial, and the importance of length of follow-up and duration of treatment, especially with respect to harms?^501^ Internal validity, the extent to which the design and conduct of the trial eliminates the possibility of bias, is a prerequisite for external validity: the results of a flawed trial are invalid and the question of its external validity becomes irrelevant. External validity is a matter of judgement and depends on the characteristics of the participants included in the trial, the trial setting, the treatment regimens tested, and the outcomes assessed.

## Discussion

It is critical that every randomised trial has a complete and transparent report of the trial methods and findings, to enable readers to judge its reliability and validity or to extract information for inclusion systematic reviews. The aim of this explanation and elaboration document is to assist authors in using CONSORT 2025 and explain in general terms the importance of adequate reporting of trials, along with providing examples of good reporting. The CONSORT 2025 statement can also help guide peer reviewers and journal editors in their evaluation of manuscripts. Indeed, we encourage peer reviewers and editors to use the CONSORT 2025 checklist to assess whether authors have reported on these items. Such assessments are likely to improve the clarity and transparency of published trials.^3^ ^502^

CONSORT is a minimum set of essential items that should be included in reports of randomised trials, published in the form of the CONSORT 2025 statement,^24^ this explanation and elaboration document, and a methods paper describing the development process.^17^

Key strengths of CONSORT 2025 include its systematic and transparent development methods, the participation of a wide range of key stakeholders (including via a large international Delphi survey), the use of empirical evidence to support its recommendations, and the availability of detailed guidance including recent examples of good reporting from published trials. Developing CONSORT 2025 and SPIRIT 2025 together was also an opportunity to align both checklists and to provide users with consistent guidance in the reporting of trial design, conduct, and analysis, from trial protocol to final publication.

A challenge with CONSORT 2025, as with any reporting guideline, is to achieve a reasonable balance between brevity versus comprehensiveness. The CONSORT 2025 checklist is intended to specify the minimum items to address in reports of randomised trials, and we have tried to keep the checklist as short as possible. We have developed an expanded checklist (appendix 1) incorporating the core content of the explanation and elaboration paper in short format, which authors may be helpful when writing their trial publication. This CONSORT 2025 explanation and elaboration paper, including its comprehensive list of references, serves to fulfil the needs of those seeking more detail and context. A joint SPIRIT-CONSORT website (https://www.consort-spirit.org/) has been established to provide more information about the CONSORT and SPIRIT statements, including additional resources and training materials aimed at researchers, research trainees, journal editors, and peer reviewers. It also includes resources aimed at patients and the public that explain the importance of transparent reporting of randomised trials and their importance in the delivery of evidence based healthcare.

The CONSORT statement is an evolving document, which requires a dynamic process of continual assessment, refinement, and, if necessary, change, which is why we have updated the checklist and this explanation and elaboration article. As new evidence and critical comments accumulate, we will evaluate the need for future updates.

## Web extra

Extra material supplied by authors

Web appendix 1: CONSORT 2025 expanded checklist of detailed information to include when reporting a randomised trial

## Notes

Contributors: SH, IB, A-WC, AH, KFS, and DM were responsible for the concept; SH, IB, A-WC, GSC, AH, KFS, and DM were responsible for the funding. SH, IB, A-WC, GSC, AH, KFS, RT, and DM drafted specific sections of the manuscript. All authors then critically revised the manuscript for important intellectual content and approved the final version. SH is the guarantor of this work. The corresponding author attests that all listed authors meet authorship criteria and that no others meeting the criteria have been omitted.

Patient and public involvement: The CONSORT 2025 checklist items and the explanations here were developed using input from an international Delphi survey and consensus meeting. The Delphi survey was advertised via established patient and public involvement (PPI) networks, and 17 respondents self-identified as a “patient or public representative” and completed the Delphi survey. In addition, three of the participants in the expert consensus meeting were patient or public representatives who were leaders in advancing PPI.

Dissemination to participants and related patient and public communities: CONSORT 2025 will be disseminated via a new website, consort-spirit.org, which will include materials designed for patients and the public.

Provenance and peer review: Not commissioned; externally peer reviewed.
